# Notes on the hosts of *Trissolcus* Ashmead (Hymenoptera: Scelionidae) from China

**DOI:** 10.3897/BDJ.8.e53786

**Published:** 2020-06-11

**Authors:** Huayan Chen, Elijah J Talamas, Hong Pang

**Affiliations:** 1 State Key Laboratory of Biocontrol, School of Life Sciences / School of Ecology, Sun Yat-sen University, Guangzhou, China State Key Laboratory of Biocontrol, School of Life Sciences / School of Ecology, Sun Yat-sen University Guangzhou China; 2 Florida State Collection of Arthropods, Gainesville, FL, United States of America Florida State Collection of Arthropods Gainesville, FL United States of America; 3 Systematic Entomology Laboratory, Washington, DC, United States of America Systematic Entomology Laboratory Washington, DC United States of America; 4 State Key Laboratory of Biocontrol, School of Ecology, Sun Yat-sen University, Guangzhou, China State Key Laboratory of Biocontrol, School of Ecology, Sun Yat-sen University Guangzhou China

**Keywords:** Biological control, egg parasitoid, Platygastroidea, stink bug

## Abstract

**Background:**

*Trissolcus* Ashmead (Hymenoptera: Scelionidae) is a cosmopolitan genus of egg-parasitoid wasps associated with stink bugs (Pentatomidae, Scutelleridae, Urostylididae), many of which are important insect pests. Documentation of host associations for these wasps, which we here provide via museum specimens, can support their use as biological control agents of invasive stink bugs.

**New information:**

The hosts of seven *Trissolcus* species are reported from China: *Trissolcus cultratus* (Mayr) (hosts: *Hippotiscus dorsalis* Stål, Pentatomidae; *Urochela luteovaria* Distant, Urostylididae), *Trissolcus elasmuchae* (Watanabe) (host: *Niphe elongata* (Dallas), Pentatomidae), *Trissolcus japonicus* (Ashmead) (hosts: *Erthesina fullo* (Thunberg), Pentatomidae; *Rhaphigaster nebulosa* (Poda), Pentatomidae), *Trissolcus latisulcus* (Crawford) (host: *Poecilocoris latus* Dallas, Scutelleridae), *Trissolcus mitsukurii* (Ashmead) (host: Pentatomidae), *Trissolcus semistriatus* (Nees von Esenbeck) (host: *Eurydema* sp., Scutelleridae), *Trissolcus yamagishii* Ryu (host: *Niphe elongata* (Dallas), Pentatomidae).

## Introduction

Species of *Trissolcus* are egg parasitoids of stink bugs, many of which are important agricultural pests. During the past decade, the Asian fauna of *Trissolcus* has received increased attention, driven largely by the search for biological control agents to manage two invasive stink bugs of global significance: *Halyomorpha halys* (Stål) and *Bagrada hilaris* (Burmeister) (Pentatomidae). The research required to develop effective biological control programs against these pests is multifaceted and includes taxonomy, behavior, ecology, molecular diagnostics and assessments of host specificity for candidate biocontrol agents (Qiu et al. 2007, Yang et al. 2009, Matsuo et al. 2014, Talamas et al. 2017, Gariepy et al. 2018, Tortorici et al. 2019, Konopka et al. 2016, Santacruz et al. 2017, Zhang et al. 2017, Boyle et al. 2019). Host range testing can be accelerated if information about parasitoid-host interactions in the native range of a pest is already available. For example, if a stink bug is known to be attacked by very few parasitoid species, this provides a shortlist for additional testing. Similarly, if a parasitoid is known to attack many species in its native range, it is unlikely to be considered sufficiently specific to be introduced in the invaded range of the pest as a biocontrol agent.

Zhejiang Univerity in Hangzhou is a leading institution in building insect collections in China, with emphasis on parasitic Hymenoptera (Chen et al. 2014). Importantly, many parasitoid wasps in this collection were reared from a variety of hosts, dating back to the 1930s and thus it contains a wealth of historical and biological information on parasitoid-host associations. However, many of these specimens, especially in the family Scelionidae, have not been identified to the species level. The current study focuses on the genus *Trissolcus* reared from field-collected eggs and is the first effort to fully investigate the parasitoid-host data of these invaluable specimens.

## Materials and methods

This work is based on specimens deposited in the Institute of Insect Sciences, Zhejiang University, Hangzhou, China (ZJU). The label data of all specimens have been georeferenced and recorded in the Hymenoptera Online database. Specimens were identified to species using the keys of Talamas et al. (2017) and Tortorici et al. (2019). Images and measurements were made using a Nikon SMZ25 microscope with a Nikon DS-Ri 2 digital camera system. Images were post-processed with Adobe Photoshop CS6 Extended.

## Taxon treatments

### Trissolcus cultratus

(Mayr) 1879

5FBD9CA1-9B37-5719-8D5C-2B8ADBBEBDD7

#### Materials

- **Type status:** Other material. **Occurrence:** catalogNumber: ZJU 878859-6; recordedBy: Qian Tan; individualCount: 1; sex: female; lifeStage: adult; associatedOccurrences: Pentatomidae; **Taxon:** scientificName: Trissolcus cultratus; order: Hymenoptera; family: Scelionidae; genus: Trissolcus; specificEpithet: cultratus; **Location:** country: China; stateProvince: Sichuan; county: Qionglai; locationRemarks: label transliteration: "Sichuan, Qionglai County, ex. from eggs of Pentatomidae, 10.vi.1986, Qian Tan"; [四川，邛崃，1986.vi.10, 谭茜；寄主：蝽卵]; **Identification:** identifiedBy: Elijah J. Talamas, Huayan Chen; dateIdentified: 2019; **Event:** samplingProtocol: none specified; **Record Level:** language: en
- **Type status:** Other material. **Occurrence:** catalogNumber: ZJU 816258-1; recordedBy: Min Huang; individualCount: 1; sex: male; lifeStage: adult; associatedOccurrences: Pentatomidae; **Taxon:** scientificName: Trissolcus cultratus; order: Hymenoptera; family: Scelionidae; genus: Trissolcus; specificEpithet: cultratus; **Location:** country: China; stateProvince: Jiangsu; county: Rugao; locationRemarks: label transliteration: "Jiangsu, Rugao, ex. from eggs of Pentatomidae, viii.1980, Min Huang"; [江苏，如皋，1980.viii，黄敏；寄主：蝽蟓卵]; **Identification:** identifiedBy: Elijah J. Talamas, Huayan Chen; dateIdentified: 2019; **Event:** samplingProtocol: none specified; eventDate: 1980-08; **Record Level:** modified: 04/29/2020; language: en
- **Type status:** Other material. **Occurrence:** catalogNumber: ZJU 816258-2; recordedBy: Min Huang; individualCount: 1; sex: male; lifeStage: adult; associatedOccurrences: Pentatomidae; **Taxon:** scientificName: Trissolcus cultratus; order: Hymenoptera; family: Scelionidae; genus: Trissolcus; specificEpithet: cultratus; **Location:** country: China; stateProvince: Jiangsu; county: Rugao; locationRemarks: label transliteration: "Jiangsu, Rugao, ex. from eggs of Pentatomidae, viii.1980, Min Huang"; [江苏，如皋，1980.viii，黄敏；寄主：蝽蟓卵]; **Identification:** identifiedBy: Elijah J. Talamas, Huayan Chen; dateIdentified: 2019; **Event:** samplingProtocol: none specified; eventDate: 1980-09; **Record Level:** modified: 04/29/2020; language: en
- **Type status:** Other material. **Occurrence:** catalogNumber: ZJU 816258-3; recordedBy: Min Huang; individualCount: 1; sex: male; lifeStage: adult; associatedOccurrences: Pentatomidae; **Taxon:** scientificName: Trissolcus cultratus; order: Hymenoptera; family: Scelionidae; genus: Trissolcus; specificEpithet: cultratus; **Location:** country: China; stateProvince: Jiangsu; county: Rugao; locationRemarks: label transliteration: "Jiangsu, Rugao, ex. from eggs of Pentatomidae, viii.1980, Min Huang"; [江苏，如皋，1980.viii，黄敏；寄主：蝽蟓卵]; **Identification:** identifiedBy: Elijah J. Talamas, Huayan Chen; dateIdentified: 2019; **Event:** samplingProtocol: none specified; eventDate: 1980-10; **Record Level:** modified: 04/29/2020; language: en
- **Type status:** Other material. **Occurrence:** catalogNumber: ZJU 816258-4; recordedBy: Min Huang; individualCount: 1; sex: male; lifeStage: adult; associatedOccurrences: Pentatomidae; **Taxon:** scientificName: Trissolcus cultratus; order: Hymenoptera; family: Scelionidae; genus: Trissolcus; specificEpithet: cultratus; **Location:** country: China; stateProvince: Jiangsu; county: Rugao; locationRemarks: label transliteration: "Jiangsu, Rugao, ex. from eggs of Pentatomidae, viii.1980, Min Huang"; [江苏，如皋，1980.viii，黄敏；寄主：蝽蟓卵]; **Identification:** identifiedBy: Elijah J. Talamas, Huayan Chen; dateIdentified: 2019; **Event:** samplingProtocol: none specified; eventDate: 1980-11; **Record Level:** modified: 04/29/2020; language: en
- **Type status:** Other material. **Occurrence:** catalogNumber: ZJU 816258-5; recordedBy: Min Huang; individualCount: 1; sex: male; lifeStage: adult; associatedOccurrences: Pentatomidae; **Taxon:** scientificName: Trissolcus cultratus; order: Hymenoptera; family: Scelionidae; genus: Trissolcus; specificEpithet: cultratus; **Location:** country: China; stateProvince: Jiangsu; county: Rugao; locationRemarks: label transliteration: "Jiangsu, Rugao, ex. from eggs of Pentatomidae, viii.1980, Min Huang"; [江苏，如皋，1980.viii，黄敏；寄主：蝽蟓卵]; **Identification:** identifiedBy: Elijah J. Talamas, Huayan Chen; dateIdentified: 2019; **Event:** samplingProtocol: none specified; eventDate: 1980-12; **Record Level:** modified: 04/29/2020; language: en
- **Type status:** Other material. **Occurrence:** catalogNumber: ZJU 816259-1; recordedBy: Min Huang; individualCount: 1; sex: female; lifeStage: adult; associatedOccurrences: Pentatomidae; **Taxon:** scientificName: Trissolcus cultratus; order: Hymenoptera; family: Scelionidae; genus: Trissolcus; specificEpithet: cultratus; **Location:** country: China; stateProvince: Jiangsu; county: Rugao; locationRemarks: label transliteration: "Jiangsu, Rugao, ex. from eggs of Pentatomidae, viii.1980, Min Huang"; [江苏，如皋，1980.viii，黄敏；寄主：蝽蟓卵]; **Identification:** identifiedBy: Elijah J. Talamas, Huayan Chen; dateIdentified: 2019; **Event:** samplingProtocol: none specified; eventDate: 1980-13; **Record Level:** modified: 04/29/2020; language: en
- **Type status:** Other material. **Occurrence:** catalogNumber: ZJU 816259-2; recordedBy: Min Huang; individualCount: 1; sex: female; lifeStage: adult; associatedOccurrences: Pentatomidae; **Taxon:** scientificName: Trissolcus cultratus; order: Hymenoptera; family: Scelionidae; genus: Trissolcus; specificEpithet: cultratus; **Location:** country: China; stateProvince: Jiangsu; county: Rugao; locationRemarks: label transliteration: "Jiangsu, Rugao, ex. from eggs of Pentatomidae, viii.1980, Min Huang"; [江苏，如皋，1980.viii，黄敏；寄主：蝽蟓卵]; **Identification:** identifiedBy: Elijah J. Talamas, Huayan Chen; dateIdentified: 2019; **Event:** samplingProtocol: none specified; eventDate: 1980-14; **Record Level:** modified: 04/29/2020; language: en
- **Type status:** Other material. **Occurrence:** catalogNumber: ZJU 816259-3; recordedBy: Min Huang; individualCount: 1; sex: female; lifeStage: adult; associatedOccurrences: Pentatomidae; **Taxon:** scientificName: Trissolcus cultratus; order: Hymenoptera; family: Scelionidae; genus: Trissolcus; specificEpithet: cultratus; **Location:** country: China; stateProvince: Jiangsu; county: Rugao; locationRemarks: label transliteration: "Jiangsu, Rugao, ex. from eggs of Pentatomidae, viii.1980, Min Huang"; [江苏，如皋，1980.viii，黄敏；寄主：蝽蟓卵]; **Identification:** identifiedBy: Elijah J. Talamas, Huayan Chen; dateIdentified: 2019; **Event:** samplingProtocol: none specified; eventDate: 1980-15; **Record Level:** modified: 04/29/2020; language: en
- **Type status:** Other material. **Occurrence:** catalogNumber: ZJU 816259-4; recordedBy: Min Huang; individualCount: 1; sex: female; lifeStage: adult; associatedOccurrences: Pentatomidae; **Taxon:** scientificName: Trissolcus cultratus; order: Hymenoptera; family: Scelionidae; genus: Trissolcus; specificEpithet: cultratus; **Location:** country: China; stateProvince: Jiangsu; county: Rugao; locationRemarks: label transliteration: "Jiangsu, Rugao, ex. from eggs of Pentatomidae, viii.1980, Min Huang"; [江苏，如皋，1980.viii，黄敏；寄主：蝽蟓卵]; **Identification:** identifiedBy: Elijah J. Talamas, Huayan Chen; dateIdentified: 2019; **Event:** samplingProtocol: none specified; eventDate: 1980-16; **Record Level:** modified: 04/29/2020; language: en
- **Type status:** Other material. **Occurrence:** catalogNumber: ZJU 816259-5; recordedBy: Min Huang; individualCount: 1; sex: female; lifeStage: adult; associatedOccurrences: Pentatomidae; **Taxon:** scientificName: Trissolcus cultratus; order: Hymenoptera; family: Scelionidae; genus: Trissolcus; specificEpithet: cultratus; **Location:** country: China; stateProvince: Jiangsu; county: Rugao; locationRemarks: label transliteration: "Jiangsu, Rugao, ex. from eggs of Pentatomidae, viii.1980, Min Huang"; [江苏，如皋，1980.viii，黄敏；寄主：蝽蟓卵]; **Identification:** identifiedBy: Elijah J. Talamas, Huayan Chen; dateIdentified: 2019; **Event:** samplingProtocol: none specified; eventDate: 1980-17; **Record Level:** modified: 04/29/2020; language: en
- **Type status:** Other material. **Occurrence:** catalogNumber: ZJU 816259-6; recordedBy: Min Huang; individualCount: 1; sex: female; lifeStage: adult; associatedOccurrences: Pentatomidae; **Taxon:** scientificName: Trissolcus cultratus; order: Hymenoptera; family: Scelionidae; genus: Trissolcus; specificEpithet: cultratus; **Location:** country: China; stateProvince: Jiangsu; county: Rugao; locationRemarks: label transliteration: "Jiangsu, Rugao, ex. from eggs of Pentatomidae, viii.1980, Min Huang"; [江苏，如皋，1980.viii，黄敏；寄主：蝽蟓卵]; **Identification:** identifiedBy: Elijah J. Talamas, Huayan Chen; dateIdentified: 2019; **Event:** samplingProtocol: none specified; eventDate: 1980-18; **Record Level:** modified: 04/29/2020; language: en
- **Type status:** Other material. **Occurrence:** catalogNumber: ZJU 816259-7; recordedBy: Min Huang; individualCount: 1; sex: female; lifeStage: adult; associatedOccurrences: Pentatomidae; **Taxon:** scientificName: Trissolcus cultratus; order: Hymenoptera; family: Scelionidae; genus: Trissolcus; specificEpithet: cultratus; **Location:** country: China; stateProvince: Jiangsu; county: Rugao; locationRemarks: label transliteration: "Jiangsu, Rugao, ex. from eggs of Pentatomidae, viii.1980, Min Huang"; [江苏，如皋，1980.viii，黄敏；寄主：蝽蟓卵]; **Identification:** identifiedBy: Elijah J. Talamas, Huayan Chen; dateIdentified: 2019; **Event:** samplingProtocol: none specified; eventDate: 1980-19; **Record Level:** modified: 04/29/2020; language: en
- **Type status:** Other material. **Occurrence:** catalogNumber: ZJU 816259-8; recordedBy: Min Huang; individualCount: 1; sex: female; lifeStage: adult; associatedOccurrences: Pentatomidae; **Taxon:** scientificName: Trissolcus cultratus; order: Hymenoptera; family: Scelionidae; genus: Trissolcus; specificEpithet: cultratus; **Location:** country: China; stateProvince: Jiangsu; county: Rugao; locationRemarks: label transliteration: "Jiangsu, Rugao, ex. from eggs of Pentatomidae, viii.1980, Min Huang"; [江苏，如皋，1980.viii，黄敏；寄主：蝽蟓卵]; **Identification:** identifiedBy: Elijah J. Talamas, Huayan Chen; dateIdentified: 2019; **Event:** samplingProtocol: none specified; eventDate: 1980-20; **Record Level:** modified: 04/29/2020; language: en
- **Type status:** Other material. **Occurrence:** catalogNumber: ZJU 814227-1; recordedBy: Junhua He; individualCount: 1; sex: female; lifeStage: adult; associatedOccurrences: Pentatomidae; **Taxon:** scientificName: Trissolcus cultratus; order: Hymenoptera; family: Scelionidae; genus: Trissolcus; specificEpithet: cultratus; **Location:** country: China; stateProvince: Guizhou; county: Guiyang; locationRemarks: label transliteration: "Guizhou, Guiyang, ex. from eggs of Pentatomidae, 14.v.1981, Junhua He"; [贵州，贵阳，1981.5.24，何俊华；寄主：蝽象]; **Identification:** identifiedBy: Elijah J. Talamas, Huayan Chen; dateIdentified: 2019; **Event:** samplingProtocol: none specified; eventDate: 05/24/1985; **Record Level:** modified: 04/29/2020; language: en
- **Type status:** Other material. **Occurrence:** catalogNumber: ZJU 814227-2; recordedBy: Junhua He; individualCount: 1; sex: female; lifeStage: adult; associatedOccurrences: Pentatomidae; **Taxon:** scientificName: Trissolcus cultratus; order: Hymenoptera; family: Scelionidae; genus: Trissolcus; specificEpithet: cultratus; **Location:** country: China; stateProvince: Guizhou; county: Guiyang; locationRemarks: label transliteration: "Guizhou, Guiyang, ex. from eggs of Pentatomidae, 14.v.1981, Junhua He"; [贵州，贵阳，1981.5.24，何俊华；寄主：蝽象]; **Identification:** identifiedBy: Elijah J. Talamas, Huayan Chen; dateIdentified: 2019; **Event:** samplingProtocol: none specified; eventDate: 05/24/1985; **Record Level:** modified: 04/29/2020; language: en
- **Type status:** Other material. **Occurrence:** catalogNumber: ZJU 800089-1; recordedBy: Tifang Feng; individualCount: 1; sex: female; lifeStage: adult; associatedOccurrences: Urochela luteovaria; **Taxon:** scientificName: Trissolcus cultratus; order: Hymenoptera; family: Scelionidae; genus: Trissolcus; specificEpithet: cultratus; **Location:** country: China; stateProvince: Qinghai; county: Minhe; locationRemarks: label transliteration: "Qinghai, Minhe, ex. from eggs of Urochela luteovaria, 25.v.1964, Tifang Feng"; [青海，民和，1964.v.25, 冯体芳；寄主：梨蝽象Urochela luteovaria]; **Identification:** identifiedBy: Elijah J. Talamas, Huayan Chen; dateIdentified: 2019; **Event:** samplingProtocol: none specified; eventDate: 05/25/1964; **Record Level:** modified: 04/29/2020; language: en
- **Type status:** Other material. **Occurrence:** catalogNumber: ZJU 800089-2; recordedBy: Tifang Feng; individualCount: 1; sex: female; lifeStage: adult; associatedOccurrences: Urochela luteovaria; **Taxon:** scientificName: Trissolcus cultratus; order: Hymenoptera; family: Scelionidae; genus: Trissolcus; specificEpithet: cultratus; **Location:** country: China; stateProvince: Qinghai; county: Minhe; locationRemarks: label transliteration: "Qinghai, Minhe, ex. from eggs of Urochela luteovaria, 25.v.1964, Tifang Feng"; [青海，民和，1964.v.25, 冯体芳；寄主：梨蝽象Urochela luteovaria]; **Identification:** identifiedBy: Elijah J. Talamas, Huayan Chen; dateIdentified: 2019; **Event:** samplingProtocol: none specified; eventDate: 05/25/1964; **Record Level:** modified: 04/29/2020; language: en
- **Type status:** Other material. **Occurrence:** catalogNumber: ZJU 853650-1; recordedBy: Maozhi Wang; individualCount: 1; sex: female; lifeStage: adult; associatedOccurrences: Hippotiscus dorsalis; **Taxon:** scientificName: Trissolcus cultratus; order: Hymenoptera; family: Scelionidae; genus: Trissolcus; specificEpithet: cultratus; **Location:** country: China; stateProvince: Zhejiang; locality: Mount Mogan; locationRemarks: label transliteration: "Zhejiang, Mount Mogan, ex. from eggs of Hippotiscus dorsalis, 1985, Maozhi Wang"; [浙江，莫干山，1985，王茂芝；寄主：竹圆蝽Hippotiscus dorsalis]; **Identification:** identifiedBy: Elijah J. Talamas, Huayan Chen; dateIdentified: 2019; **Event:** samplingProtocol: none specified; eventDate: 1985; **Record Level:** modified: 04/29/2020; language: en
- **Type status:** Other material. **Occurrence:** catalogNumber: ZJU 853650-2; recordedBy: Maozhi Wang; individualCount: 1; sex: female; lifeStage: adult; associatedOccurrences: Hippotiscus dorsalis; **Taxon:** scientificName: Trissolcus cultratus; order: Hymenoptera; family: Scelionidae; genus: Trissolcus; specificEpithet: cultratus; **Location:** country: China; stateProvince: Zhejiang; locality: Mount Mogan; locationRemarks: label transliteration: "Zhejiang, Mount Mogan, ex. from eggs of Hippotiscus dorsalis, 1985, Maozhi Wang"; [浙江，莫干山，1985，王茂芝；寄主：竹圆蝽Hippotiscus dorsalis]; **Identification:** identifiedBy: Elijah J. Talamas, Huayan Chen; dateIdentified: 2019; **Event:** samplingProtocol: none specified; eventDate: 1985; **Record Level:** modified: 04/29/2020; language: en
- **Type status:** Other material. **Occurrence:** catalogNumber: ZJU 853649; recordedBy: Maozhi Wang; individualCount: 1; sex: female; lifeStage: adult; associatedOccurrences: Hippotiscus dorsalis; **Taxon:** scientificName: Trissolcus cultratus; order: Hymenoptera; family: Scelionidae; genus: Trissolcus; specificEpithet: cultratus; **Location:** country: China; stateProvince: Zhejiang; locality: Mount Mogan; locationRemarks: label transliteration: "Zhejiang, Mount Mogan, ex. from eggs of Hippotiscus dorsalis, 1985, Maozhi Wang"; [浙江，莫干山，1985，王茂芝；寄主：竹圆蝽Hippotiscus dorsalis]; **Identification:** identifiedBy: Elijah J. Talamas, Huayan Chen; dateIdentified: 2019; **Event:** samplingProtocol: none specified; eventDate: 1985; **Record Level:** modified: 04/29/2020; language: en
- **Type status:** Other material. **Occurrence:** catalogNumber: ZJU 740720-1; individualCount: 1; sex: male, egg mass; lifeStage: adult; associatedOccurrences: Pentatomidae; **Taxon:** scientificName: Trissolcus cultratus; order: Hymenoptera; family: Scelionidae; genus: Trissolcus; specificEpithet: cultratus; **Location:** country: China; stateProvince: Shaanxi; county: Hanzhong; locationRemarks: label transliteration: "Shaanxi, Hanzhong, ex. from eggs of Pentatomidae in paddy field, ix.1974"; [陕西，汉中，1974.ix；寄主：稻蝽象]; **Identification:** identifiedBy: Elijah J. Talamas, Huayan Chen; dateIdentified: 2019; **Event:** samplingProtocol: none specified; eventDate: 1974-11; **Record Level:** modified: 04/29/2020; language: en
- **Type status:** Other material. **Occurrence:** catalogNumber: ZJU 740720-2; individualCount: 1; sex: male; lifeStage: adult; associatedOccurrences: Pentatomidae; **Taxon:** scientificName: Trissolcus cultratus; order: Hymenoptera; family: Scelionidae; genus: Trissolcus; specificEpithet: cultratus; **Location:** country: China; stateProvince: Shaanxi; county: Hanzhong; locationRemarks: label transliteration: "Shaanxi, Hanzhong, ex. from eggs of Pentatomidae in paddy field, ix.1974"; [陕西，汉中，1974.ix；寄主：稻蝽象]; **Identification:** identifiedBy: Elijah J. Talamas, Huayan Chen; dateIdentified: 2019; **Event:** samplingProtocol: none specified; eventDate: 1974-11; **Record Level:** modified: 04/29/2020; language: en
- **Type status:** Other material. **Occurrence:** catalogNumber: ZJU 740720-3; individualCount: 1; sex: male; lifeStage: adult; associatedOccurrences: Pentatomidae; **Taxon:** scientificName: Trissolcus cultratus; order: Hymenoptera; family: Scelionidae; genus: Trissolcus; specificEpithet: cultratus; **Location:** country: China; stateProvince: Shaanxi; county: Hanzhong; locationRemarks: label transliteration: "Shaanxi, Hanzhong, ex. from eggs of Pentatomidae in paddy field, ix.1974"; [陕西，汉中，1974.ix；寄主：稻蝽象]; **Identification:** identifiedBy: Elijah J. Talamas, Huayan Chen; dateIdentified: 2019; **Event:** samplingProtocol: none specified; eventDate: 1974-11; **Record Level:** modified: 04/29/2020; language: en
- **Type status:** Other material. **Occurrence:** catalogNumber: ZJU 740720-4; individualCount: 1; sex: male; lifeStage: adult; associatedOccurrences: Pentatomidae; **Taxon:** scientificName: Trissolcus cultratus; order: Hymenoptera; family: Scelionidae; genus: Trissolcus; specificEpithet: cultratus; **Location:** country: China; stateProvince: Shaanxi; county: Hanzhong; locationRemarks: label transliteration: "Shaanxi, Hanzhong, ex. from eggs of Pentatomidae in paddy field, ix.1974"; [陕西，汉中，1974.ix；寄主：稻蝽象]; **Identification:** identifiedBy: Elijah J. Talamas, Huayan Chen; dateIdentified: 2019; **Event:** samplingProtocol: none specified; eventDate: 1974-11; **Record Level:** modified: 04/29/2020; language: en

#### Distribution

China (Qinghai, Shaanxi, Sichuan, Guizhou, Jiangsu, Zhejiang, Taiwan); Austria, Belgium, Czech Republic, France, Germany, Hungary, Japan, Poland, Russia, South Korea, Sweden, Switzerland and United Kingdom (Talamas et al. 2017).

#### Biology

**Host associations.** This species was reared from the eggs of *Hippotiscus dorsalis* Stål (Pentatomidae), *Urochela luteovaria* Distant (Urostylididae) and some unidentified species of Pentatomidae in China.

#### Notes

##### Images

*Trissolcus cultratus* Mayr 1879 (Fig. 1).

### Trissolcus elasmuchae

(Watanabe) 1954

1043381B-56A4-5565-86BA-9AACD94DB959

#### Materials

- **Type status:** Other material. **Occurrence:** catalogNumber: ZJU 5728.17-1; recordedBy: Junhua He; individualCount: 3; sex: 2 female, 1 male, egg mass; lifeStage: adult; associatedOccurrences: Niphe elongata; **Taxon:** scientificName: Trissolcus elasmuchae; order: Hymenoptera; family: Scelionidae; genus: Trissolcus; specificEpithet: elasmuchae; **Location:** country: China; stateProvince: Zhejiang; county: Hangzhou; locationRemarks: label transliteration: "Zhejiang, Hangzhou, ex. from eggs of Niphe elongata, 18.vii.1957, Junhua He"; [浙江，杭州，1957.vii.18, 何俊华；寄主：褐蝽象卵Niphe elongata]; **Identification:** identifiedBy: Elijah J. Talamas, Huayan Chen; dateIdentified: 2019; **Event:** samplingProtocol: none specified; eventDate: 07/18/1957; **Record Level:** modified: 04/29/2020; language: en
- **Type status:** Other material. **Occurrence:** catalogNumber: ZJU 5728.17-2; recordedBy: Junhua He; individualCount: 3; sex: 2 female, 1 male, egg mass; lifeStage: adult; associatedOccurrences: Niphe elongata; **Taxon:** scientificName: Trissolcus elasmuchae; order: Hymenoptera; family: Scelionidae; genus: Trissolcus; specificEpithet: elasmuchae; **Location:** country: China; stateProvince: Zhejiang; county: Hangzhou; locationRemarks: label transliteration: "Zhejiang, Hangzhou, ex. from eggs of Niphe elongata, 18.vii.1957, Junhua He"; [浙江，杭州，1957.vii.18, 何俊华；寄主：褐蝽象卵Niphe elongata]; **Identification:** identifiedBy: Elijah J. Talamas, Huayan Chen; dateIdentified: 2019; **Event:** samplingProtocol: none specified; eventDate: 07/18/1957; **Record Level:** modified: 04/29/2020; language: en
- **Type status:** Other material. **Occurrence:** catalogNumber: ZJU 5728.17-3; recordedBy: Junhua He; individualCount: 3; sex: 1 female，2 males; lifeStage: adult; associatedOccurrences: Niphe elongata; **Taxon:** scientificName: Trissolcus elasmuchae; order: Hymenoptera; family: Scelionidae; genus: Trissolcus; specificEpithet: elasmuchae; **Location:** country: China; stateProvince: Zhejiang; county: Hangzhou; locationRemarks: label transliteration: "Zhejiang, Hangzhou, ex. from eggs of Niphe elongata, 18.vii.1957, Junhua He"; [浙江，杭州，1957.vii.18, 何俊华；寄主：褐蝽象卵Niphe elongata]; **Identification:** identifiedBy: Elijah J. Talamas, Huayan Chen; dateIdentified: 2019; **Event:** samplingProtocol: none specified; eventDate: 07/18/1957; **Record Level:** modified: 04/29/2020; language: en
- **Type status:** Other material. **Occurrence:** catalogNumber: ZJU 5728.17-4; recordedBy: Junhua He; individualCount: 3; sex: 3 females，egg mass; lifeStage: adult; associatedOccurrences: Niphe elongata; **Taxon:** scientificName: Trissolcus elasmuchae; order: Hymenoptera; family: Scelionidae; genus: Trissolcus; specificEpithet: elasmuchae; **Location:** country: China; stateProvince: Zhejiang; county: Hangzhou; locationRemarks: label transliteration: "Zhejiang, Hangzhou, ex. from eggs of Niphe elongata, 18.vii.1957, Junhua He"; [浙江，杭州，1957.vii.18, 何俊华；寄主：褐蝽象卵Niphe elongata]; **Identification:** identifiedBy: Elijah J. Talamas, Huayan Chen; dateIdentified: 2019; **Event:** samplingProtocol: none specified; eventDate: 07/18/1957; **Record Level:** modified: 04/29/2020; language: en

#### Distribution

China (Zhejiang, Taiwan); France, India, Japan, South Korea, Sweden, United Kingdom and Vietnam (Talamas et al. 2017).

#### Biology

**Host associations.** This species was reared from the eggs of *Niphe elongata* (Dallas) (Pentatomidae) in China.

#### Images

*Trissolcus elasmuchaeWatanabe 1954* (Fig. 2).

### Trissolcus japonicus

(Ashmead) 1904

7E3B9756-8217-5B45-B940-7446597153C6

#### Materials

- **Type status:** Other material. **Occurrence:** catalogNumber: ZJU 853338-1; recordedBy: Hanlin Chen; individualCount: 1; sex: female; lifeStage: adult; associatedOccurrences: Erthesina fullo; **Taxon:** scientificName: Trissolcus japonicus; order: Hymenoptera; family: Scelionidae; genus: Trissolcus; specificEpithet: japonicus; vernacularName: samurai wasp; **Location:** country: China; stateProvince: Zhejiang; county: Songyang; locationRemarks: label transliteration: "Zhejiang, Songyang County, ex. from eggs of Erthesina fullo, 14.viii.1985, Hanlin Chen"; [浙江，松阳，1985.viii.14, 陈汉林；寄主：麻皮蝽卵]; **Identification:** identifiedBy: Elijah J. Talamas, Huayan Chen; dateIdentified: 2019; **Event:** samplingProtocol: none specified; eventDate: 08/14/1985; **Record Level:** modified: 04/29/2020; language: en; collectionCode: Insects; basisOfRecord: PreservedSpecimen
- **Type status:** Other material. **Occurrence:** catalogNumber: ZJU 853338-2; recordedBy: Hanlin Chen; individualCount: 1; sex: male; lifeStage: adult; associatedOccurrences: Erthesina fullo; **Taxon:** scientificName: Trissolcus japonicus; order: Hymenoptera; family: Scelionidae; genus: Trissolcus; specificEpithet: japonicus; vernacularName: samurai wasp; **Location:** country: China; stateProvince: Zhejiang; county: Songyang; locationRemarks: label transliteration: "Zhejiang, Songyang County, ex. from eggs of Erthesina fullo, 14.viii.1985, Hanlin Chen"; [浙江，松阳，1985.viii.14, 陈汉林；寄主：麻皮蝽卵]; **Identification:** identifiedBy: Elijah J. Talamas, Huayan Chen; dateIdentified: 2019; **Event:** samplingProtocol: none specified; eventDate: 08/14/1985; **Record Level:** modified: 04/29/2020; language: en; collectionCode: Insects; basisOfRecord: PreservedSpecimen
- **Type status:** Other material. **Occurrence:** catalogNumber: ZJU 853338-3; recordedBy: Hanlin Chen; individualCount: 1; sex: female; lifeStage: adult; associatedOccurrences: Erthesina fullo; **Taxon:** scientificName: Trissolcus japonicus; order: Hymenoptera; family: Scelionidae; genus: Trissolcus; specificEpithet: japonicus; vernacularName: samurai wasp; **Location:** country: China; stateProvince: Zhejiang; county: Songyang; locationRemarks: label transliteration: "Zhejiang, Songyang County, ex. from eggs of Erthesina fullo, 14.viii.1985, Hanlin Chen"; [浙江，松阳，1985.viii.14, 陈汉林；寄主：麻皮蝽卵]; **Identification:** identifiedBy: Elijah J. Talamas, Huayan Chen; dateIdentified: 2019; **Event:** samplingProtocol: none specified; eventDate: 08/14/1985; **Record Level:** modified: 04/29/2020; language: en; collectionCode: Insects; basisOfRecord: PreservedSpecimen
- **Type status:** Other material. **Occurrence:** catalogNumber: ZJU 853338-4; recordedBy: Hanlin Chen; individualCount: 1; sex: female; lifeStage: adult; associatedOccurrences: Erthesina fullo; **Taxon:** scientificName: Trissolcus japonicus; order: Hymenoptera; family: Scelionidae; genus: Trissolcus; specificEpithet: japonicus; vernacularName: samurai wasp; **Location:** country: China; stateProvince: Zhejiang; county: Songyang; locationRemarks: label transliteration: "Zhejiang, Songyang County, ex. from eggs of Erthesina fullo, 14.viii.1985, Hanlin Chen"; [浙江，松阳，1985.viii.14, 陈汉林；寄主：麻皮蝽卵]; **Identification:** identifiedBy: Elijah J. Talamas, Huayan Chen; dateIdentified: 2019; **Event:** samplingProtocol: none specified; eventDate: 08/14/1985; **Record Level:** modified: 04/29/2020; language: en; collectionCode: Insects; basisOfRecord: PreservedSpecimen
- **Type status:** Other material. **Occurrence:** catalogNumber: ZJU 853338-5; recordedBy: Hanlin Chen; individualCount: 1; sex: female; lifeStage: adult; associatedOccurrences: Erthesina fullo; **Taxon:** scientificName: Trissolcus japonicus; order: Hymenoptera; family: Scelionidae; genus: Trissolcus; specificEpithet: japonicus; vernacularName: samurai wasp; **Location:** country: China; stateProvince: Zhejiang; county: Songyang; locationRemarks: label transliteration: "Zhejiang, Songyang County, ex. from eggs of Erthesina fullo, 14.viii.1985, Hanlin Chen"; [浙江，松阳，1985.viii.14, 陈汉林；寄主：麻皮蝽卵]; **Identification:** identifiedBy: Elijah J. Talamas, Huayan Chen; dateIdentified: 2019; **Event:** samplingProtocol: none specified; eventDate: 08/14/1985; **Record Level:** modified: 04/29/2020; language: en; collectionCode: Insects; basisOfRecord: PreservedSpecimen
- **Type status:** Other material. **Occurrence:** catalogNumber: ZJU 853338-6; recordedBy: Hanlin Chen; individualCount: 1; sex: female; lifeStage: adult; associatedOccurrences: Erthesina fullo; **Taxon:** scientificName: Trissolcus japonicus; order: Hymenoptera; family: Scelionidae; genus: Trissolcus; specificEpithet: japonicus; vernacularName: samurai wasp; **Location:** country: China; stateProvince: Zhejiang; county: Songyang; locationRemarks: label transliteration: "Zhejiang, Songyang County, ex. from eggs of Erthesina fullo, 14.viii.1985, Hanlin Chen"; [浙江，松阳，1985.viii.14, 陈汉林；寄主：麻皮蝽卵]; **Identification:** identifiedBy: Elijah J. Talamas, Huayan Chen; dateIdentified: 2019; **Event:** samplingProtocol: none specified; eventDate: 08/14/1985; **Record Level:** modified: 04/29/2020; language: en; collectionCode: Insects; basisOfRecord: PreservedSpecimen
- **Type status:** Other material. **Occurrence:** catalogNumber: ZJU 853338-7; recordedBy: Hanlin Chen; individualCount: 1; sex: female; lifeStage: adult; associatedOccurrences: Erthesina fullo; **Taxon:** scientificName: Trissolcus japonicus; order: Hymenoptera; family: Scelionidae; genus: Trissolcus; specificEpithet: japonicus; vernacularName: samurai wasp; **Location:** country: China; stateProvince: Zhejiang; county: Songyang; locationRemarks: label transliteration: "Zhejiang, Songyang County, ex. from eggs of Erthesina fullo, 14.viii.1985, Hanlin Chen"; [浙江，松阳，1985.viii.14, 陈汉林；寄主：麻皮蝽卵]; **Identification:** identifiedBy: Elijah J. Talamas, Huayan Chen; dateIdentified: 2019; **Event:** samplingProtocol: none specified; eventDate: 08/14/1985; **Record Level:** modified: 04/29/2020; language: en; collectionCode: Insects; basisOfRecord: PreservedSpecimen
- **Type status:** Other material. **Occurrence:** catalogNumber: ZJU 853338-8; recordedBy: Hanlin Chen; individualCount: 1; sex: female; lifeStage: adult; associatedOccurrences: Erthesina fullo; **Taxon:** scientificName: Trissolcus japonicus; order: Hymenoptera; family: Scelionidae; genus: Trissolcus; specificEpithet: japonicus; vernacularName: samurai wasp; **Location:** country: China; stateProvince: Zhejiang; county: Songyang; locationRemarks: label transliteration: "Zhejiang, Songyang County, ex. from eggs of Erthesina fullo, 14.viii.1985, Hanlin Chen"; [浙江，松阳，1985.viii.14, 陈汉林；寄主：麻皮蝽卵]; **Identification:** identifiedBy: Elijah J. Talamas, Huayan Chen; dateIdentified: 2019; **Event:** samplingProtocol: none specified; eventDate: 08/14/1985; **Record Level:** modified: 04/29/2020; language: en; collectionCode: Insects; basisOfRecord: PreservedSpecimen
- **Type status:** Other material. **Occurrence:** catalogNumber: ZJU 740411-1; recordedBy: Gansu Agricultural University; individualCount: 1; sex: female; lifeStage: adult; associatedOccurrences: Rhaphigaster nebulosa; **Taxon:** scientificName: Trissolcus japonicus; order: Hymenoptera; family: Scelionidae; genus: Trissolcus; specificEpithet: japonicus; vernacularName: samurai wasp; **Location:** country: China; stateProvince: Gansu; county: Wuwei; locationRemarks: label transliteration: "Gansu, Wuwei, ex. from eggs of Rhaphigaster nebulosa, v.1974, Gansu Agricultural University"; [甘肃，武威，1974.5，甘肃农大；寄主：沙枣蝽Rhaphigaster nebulosa]; **Identification:** identifiedBy: Elijah J. Talamas, Huayan Chen; dateIdentified: 2019; **Event:** samplingProtocol: none specified; eventDate: 1974-05; **Record Level:** modified: 04/29/2020; language: en; collectionCode: Insects; basisOfRecord: PreservedSpecimen
- **Type status:** Other material. **Occurrence:** catalogNumber: ZJU 740411-2; recordedBy: Gansu Agricultural University; individualCount: 1; sex: female; lifeStage: adult; associatedOccurrences: Rhaphigaster nebulosa; **Taxon:** scientificName: Trissolcus japonicus; order: Hymenoptera; family: Scelionidae; genus: Trissolcus; specificEpithet: japonicus; vernacularName: samurai wasp; **Location:** country: China; stateProvince: Gansu; county: Wuwei; locationRemarks: label transliteration: "Gansu, Wuwei, ex. from eggs of Rhaphigaster nebulosa, v.1974, Gansu Agricultural University"; [甘肃，武威，1974.5，甘肃农大；寄主：沙枣蝽Rhaphigaster nebulosa]; **Identification:** identifiedBy: Elijah J. Talamas, Huayan Chen; dateIdentified: 2019; **Event:** samplingProtocol: none specified; eventDate: 1974-05; **Record Level:** modified: 04/29/2020; language: en; collectionCode: Insects; basisOfRecord: PreservedSpecimen
- **Type status:** Other material. **Occurrence:** catalogNumber: ZJU 740411-3; recordedBy: Gansu Agricultural University; individualCount: 1; sex: male; lifeStage: adult; associatedOccurrences: Rhaphigaster nebulosa; **Taxon:** scientificName: Trissolcus japonicus; order: Hymenoptera; family: Scelionidae; genus: Trissolcus; specificEpithet: japonicus; vernacularName: samurai wasp; **Location:** country: China; stateProvince: Gansu; county: Wuwei; locationRemarks: label transliteration: "Gansu, Wuwei, ex. from eggs of Rhaphigaster nebulosa, v.1974, Gansu Agricultural University"; [甘肃，武威，1974.5，甘肃农大；寄主：沙枣蝽Rhaphigaster nebulosa]; **Identification:** identifiedBy: Elijah J. Talamas, Huayan Chen; dateIdentified: 2019; **Event:** samplingProtocol: none specified; eventDate: 1974-05; **Record Level:** modified: 04/29/2020; language: en; collectionCode: Insects; basisOfRecord: PreservedSpecimen
- **Type status:** Other material. **Occurrence:** catalogNumber: ZJU 740411-4; recordedBy: Gansu Agricultural University; individualCount: 1; sex: female; lifeStage: adult; associatedOccurrences: Rhaphigaster nebulosa; **Taxon:** scientificName: Trissolcus japonicus; order: Hymenoptera; family: Scelionidae; genus: Trissolcus; specificEpithet: japonicus; vernacularName: samurai wasp; **Location:** country: China; stateProvince: Gansu; county: Wuwei; locationRemarks: label transliteration: "Gansu, Wuwei, ex. from eggs of Rhaphigaster nebulosa, v.1974, Gansu Agricultural University"; [甘肃，武威，1974.5，甘肃农大；寄主：沙枣蝽Rhaphigaster nebulosa]; **Identification:** identifiedBy: Elijah J. Talamas, Huayan Chen; dateIdentified: 2019; **Event:** samplingProtocol: none specified; eventDate: 1974-05; **Record Level:** modified: 04/29/2020; language: en; collectionCode: Insects; basisOfRecord: PreservedSpecimen
- **Type status:** Other material. **Occurrence:** catalogNumber: ZJU 740411-5; recordedBy: Gansu Agricultural University; individualCount: 1; sex: male; lifeStage: adult; associatedOccurrences: Rhaphigaster nebulosa; **Taxon:** scientificName: Trissolcus japonicus; order: Hymenoptera; family: Scelionidae; genus: Trissolcus; specificEpithet: japonicus; vernacularName: samurai wasp; **Location:** country: China; stateProvince: Gansu; county: Wuwei; locationRemarks: label transliteration: "Gansu, Wuwei, ex. from eggs of Rhaphigaster nebulosa, v.1974, Gansu Agricultural University"; [甘肃，武威，1974.5，甘肃农大；寄主：沙枣蝽Rhaphigaster nebulosa]; **Identification:** identifiedBy: Elijah J. Talamas, Huayan Chen; dateIdentified: 2019; **Event:** samplingProtocol: none specified; eventDate: 1974-05; **Record Level:** modified: 04/29/2020; language: en; collectionCode: Insects; basisOfRecord: PreservedSpecimen
- **Type status:** Other material. **Occurrence:** catalogNumber: ZJU 740411-6; recordedBy: Gansu Agricultural University; individualCount: 1; sex: female; lifeStage: adult; associatedOccurrences: Rhaphigaster nebulosa; **Taxon:** scientificName: Trissolcus japonicus; order: Hymenoptera; family: Scelionidae; genus: Trissolcus; specificEpithet: japonicus; vernacularName: samurai wasp; **Location:** country: China; stateProvince: Gansu; county: Wuwei; locationRemarks: label transliteration: "Gansu, Wuwei, ex. from eggs of Rhaphigaster nebulosa, v.1974, Gansu Agricultural University"; [甘肃，武威，1974.5，甘肃农大；寄主：沙枣蝽Rhaphigaster nebulosa]; **Identification:** identifiedBy: Elijah J. Talamas, Huayan Chen; dateIdentified: 2019; **Event:** samplingProtocol: none specified; eventDate: 1974-05; **Record Level:** modified: 04/29/2020; language: en; collectionCode: Insects; basisOfRecord: PreservedSpecimen
- **Type status:** Other material. **Occurrence:** catalogNumber: ZJU 740411-7; recordedBy: Gansu Agricultural University; individualCount: 1; sex: male; lifeStage: adult; associatedOccurrences: Rhaphigaster nebulosa; **Taxon:** scientificName: Trissolcus japonicus; order: Hymenoptera; family: Scelionidae; genus: Trissolcus; specificEpithet: japonicus; vernacularName: samurai wasp; **Location:** country: China; stateProvince: Gansu; county: Wuwei; locationRemarks: label transliteration: "Gansu, Wuwei, ex. from eggs of Rhaphigaster nebulosa, v.1974, Gansu Agricultural University"; [甘肃，武威，1974.5，甘肃农大；寄主：沙枣蝽Rhaphigaster nebulosa]; **Identification:** identifiedBy: Elijah J. Talamas, Huayan Chen; dateIdentified: 2019; **Event:** samplingProtocol: none specified; eventDate: 1974-05; **Record Level:** modified: 04/29/2020; language: en; collectionCode: Insects; basisOfRecord: PreservedSpecimen
- **Type status:** Other material. **Occurrence:** catalogNumber: ZJU 740411-8; recordedBy: Gansu Agricultural University; individualCount: 1; sex: female; lifeStage: adult; associatedOccurrences: Rhaphigaster nebulosa; **Taxon:** scientificName: Trissolcus japonicus; order: Hymenoptera; family: Scelionidae; genus: Trissolcus; specificEpithet: japonicus; vernacularName: samurai wasp; **Location:** country: China; stateProvince: Gansu; county: Wuwei; locationRemarks: label transliteration: "Gansu, Wuwei, ex. from eggs of Rhaphigaster nebulosa, v.1974, Gansu Agricultural University"; [甘肃，武威，1974.5，甘肃农大；寄主：沙枣蝽Rhaphigaster nebulosa]; **Identification:** identifiedBy: Elijah J. Talamas, Huayan Chen; dateIdentified: 2019; **Event:** samplingProtocol: none specified; eventDate: 1974-05; **Record Level:** modified: 04/29/2020; language: en; collectionCode: Insects; basisOfRecord: PreservedSpecimen
- **Type status:** Other material. **Occurrence:** catalogNumber: ZJU 740411-9; recordedBy: Gansu Agricultural University; individualCount: 1; sex: female; lifeStage: adult; associatedOccurrences: Rhaphigaster nebulosa; **Taxon:** scientificName: Trissolcus japonicus; order: Hymenoptera; family: Scelionidae; genus: Trissolcus; specificEpithet: japonicus; vernacularName: samurai wasp; **Location:** country: China; stateProvince: Gansu; county: Wuwei; locationRemarks: label transliteration: "Gansu, Wuwei, ex. from eggs of Rhaphigaster nebulosa, v.1974, Gansu Agricultural University"; [甘肃，武威，1974.5，甘肃农大；寄主：沙枣蝽Rhaphigaster nebulosa]; **Identification:** identifiedBy: Elijah J. Talamas, Huayan Chen; dateIdentified: 2019; **Event:** samplingProtocol: none specified; eventDate: 1974-05; **Record Level:** modified: 04/29/2020; language: en; collectionCode: Insects; basisOfRecord: PreservedSpecimen
- **Type status:** Other material. **Occurrence:** catalogNumber: ZJU 740411-10; recordedBy: Gansu Agricultural University; individualCount: 1; sex: female; lifeStage: adult; associatedOccurrences: Rhaphigaster nebulosa; **Taxon:** scientificName: Trissolcus japonicus; order: Hymenoptera; family: Scelionidae; genus: Trissolcus; specificEpithet: japonicus; vernacularName: samurai wasp; **Location:** country: China; stateProvince: Gansu; county: Wuwei; locationRemarks: label transliteration: "Gansu, Wuwei, ex. from eggs of Rhaphigaster nebulosa, v.1974, Gansu Agricultural University"; [甘肃，武威，1974.5，甘肃农大；寄主：沙枣蝽Rhaphigaster nebulosa]; **Identification:** identifiedBy: Elijah J. Talamas, Huayan Chen; dateIdentified: 2019; **Event:** samplingProtocol: none specified; eventDate: 1974-05; **Record Level:** modified: 04/29/2020; language: en; collectionCode: Insects; basisOfRecord: PreservedSpecimen
- **Type status:** Other material. **Occurrence:** catalogNumber: ZJU 740412-1; recordedBy: Gansu Agricultural University; individualCount: 1; sex: male; lifeStage: adult; associatedOccurrences: Rhaphigaster nebulosa; **Taxon:** scientificName: Trissolcus japonicus; order: Hymenoptera; family: Scelionidae; genus: Trissolcus; specificEpithet: japonicus; vernacularName: samurai wasp; **Location:** country: China; stateProvince: Gansu; county: Linze; locationRemarks: label transliteration: "Gansu, Linze County, ex.from eggs of Rhaphigaster nebulosa, vi.1974, Gansu Agricultural University"; [甘肃，临泽，1974.6，甘肃农大；寄主：沙枣蝽Rhaphigaster nebulosa]; **Identification:** identifiedBy: Elijah J. Talamas, Huayan Chen; dateIdentified: 2019; **Event:** samplingProtocol: none specified; eventDate: 1974-06; **Record Level:** modified: 04/29/2020; language: en; collectionCode: Insects; basisOfRecord: PreservedSpecimen
- **Type status:** Other material. **Occurrence:** catalogNumber: ZJU 740412-2; recordedBy: Gansu Agricultural University; individualCount: 1; sex: male; lifeStage: adult; associatedOccurrences: Rhaphigaster nebulosa; **Taxon:** scientificName: Trissolcus japonicus; order: Hymenoptera; family: Scelionidae; genus: Trissolcus; specificEpithet: japonicus; vernacularName: samurai wasp; **Location:** country: China; stateProvince: Gansu; county: Linze; locationRemarks: label transliteration: "Gansu, Linze County, ex.from eggs of Rhaphigaster nebulosa, vi.1974, Gansu Agricultural University"; [甘肃，临泽，1974.6，甘肃农大；寄主：沙枣蝽Rhaphigaster nebulosa]; **Identification:** identifiedBy: Elijah J. Talamas, Huayan Chen; dateIdentified: 2019; **Event:** samplingProtocol: none specified; eventDate: 1974-06; **Record Level:** modified: 04/29/2020; language: en; collectionCode: Insects; basisOfRecord: PreservedSpecimen
- **Type status:** Other material. **Occurrence:** catalogNumber: ZJU 740412-3; recordedBy: Gansu Agricultural University; individualCount: 1; sex: male; lifeStage: adult; associatedOccurrences: Rhaphigaster nebulosa; **Taxon:** scientificName: Trissolcus japonicus; order: Hymenoptera; family: Scelionidae; genus: Trissolcus; specificEpithet: japonicus; vernacularName: samurai wasp; **Location:** country: China; stateProvince: Gansu; county: Linze; locationRemarks: label transliteration: "Gansu, Linze County, ex.from eggs of Rhaphigaster nebulosa, vi.1974, Gansu Agricultural University"; [甘肃，临泽，1974.6，甘肃农大；寄主：沙枣蝽Rhaphigaster nebulosa]; **Identification:** identifiedBy: Elijah J. Talamas, Huayan Chen; dateIdentified: 2019; **Event:** samplingProtocol: none specified; eventDate: 1974-06; **Record Level:** modified: 04/29/2020; language: en; collectionCode: Insects; basisOfRecord: PreservedSpecimen
- **Type status:** Other material. **Occurrence:** catalogNumber: ZJU 740412-4; recordedBy: Gansu Agricultural University; individualCount: 1; sex: female; lifeStage: adult; associatedOccurrences: Rhaphigaster nebulosa; **Taxon:** scientificName: Trissolcus japonicus; order: Hymenoptera; family: Scelionidae; genus: Trissolcus; specificEpithet: japonicus; vernacularName: samurai wasp; **Location:** country: China; stateProvince: Gansu; county: Linze; locationRemarks: label transliteration: "Gansu, Linze County, ex.from eggs of Rhaphigaster nebulosa, vi.1974, Gansu Agricultural University"; [甘肃，临泽，1974.6，甘肃农大；寄主：沙枣蝽Rhaphigaster nebulosa]; **Identification:** identifiedBy: Elijah J. Talamas, Huayan Chen; dateIdentified: 2019; **Event:** samplingProtocol: none specified; eventDate: 1974-06; **Record Level:** modified: 04/29/2020; language: en; collectionCode: Insects; basisOfRecord: PreservedSpecimen
- **Type status:** Other material. **Occurrence:** catalogNumber: ZJU 740412-5; recordedBy: Gansu Agricultural University; individualCount: 1; sex: male; lifeStage: adult; associatedOccurrences: Rhaphigaster nebulosa; **Taxon:** scientificName: Trissolcus japonicus; order: Hymenoptera; family: Scelionidae; genus: Trissolcus; specificEpithet: japonicus; vernacularName: samurai wasp; **Location:** country: China; stateProvince: Gansu; county: Linze; locationRemarks: label transliteration: "Gansu, Linze County, ex.from eggs of Rhaphigaster nebulosa, vi.1974, Gansu Agricultural University"; [甘肃，临泽，1974.6，甘肃农大；寄主：沙枣蝽Rhaphigaster nebulosa]; **Identification:** identifiedBy: Elijah J. Talamas, Huayan Chen; dateIdentified: 2019; **Event:** samplingProtocol: none specified; eventDate: 1974-06; **Record Level:** modified: 04/29/2020; language: en; collectionCode: Insects; basisOfRecord: PreservedSpecimen
- **Type status:** Other material. **Occurrence:** catalogNumber: ZJU 740412-6; recordedBy: Gansu Agricultural University; individualCount: 1; sex: female; lifeStage: adult; associatedOccurrences: Rhaphigaster nebulosa; **Taxon:** scientificName: Trissolcus japonicus; order: Hymenoptera; family: Scelionidae; genus: Trissolcus; specificEpithet: japonicus; vernacularName: samurai wasp; **Location:** country: China; stateProvince: Gansu; county: Linze; locationRemarks: label transliteration: "Gansu, Linze County, ex.from eggs of Rhaphigaster nebulosa, vi.1974, Gansu Agricultural University"; [甘肃，临泽，1974.6，甘肃农大；寄主：沙枣蝽Rhaphigaster nebulosa]; **Identification:** identifiedBy: Elijah J. Talamas, Huayan Chen; dateIdentified: 2019; **Event:** samplingProtocol: none specified; eventDate: 1974-06; **Record Level:** modified: 04/29/2020; language: en; collectionCode: Insects; basisOfRecord: PreservedSpecimen
- **Type status:** Other material. **Occurrence:** catalogNumber: ZJU 5727.1-1; recordedBy: Xuedi Li; individualCount: 3; sex: 2 female, 1 male, egg mass; lifeStage: adult; associatedOccurrences: Pentatomidae; **Taxon:** scientificName: Trissolcus japonicus; order: Hymenoptera; family: Scelionidae; genus: Trissolcus; specificEpithet: japonicus; vernacularName: samurai wasp; **Location:** country: China; stateProvince: Zhejiang; county: Hangzhou; locationRemarks: label transliteration: "Zhejiang, Hangzhou, ex. from eggs of Pentatomidae, 24.v.1957, Xuedi Li"; [浙江，杭州，1957.v.24,李学迪；寄主：蝽象卵]; **Identification:** identifiedBy: Elijah J. Talamas, Huayan Chen; dateIdentified: 2019; **Event:** samplingProtocol: none specified; eventDate: 05/24/1957; **Record Level:** modified: 04/29/2020; language: en
- **Type status:** Other material. **Occurrence:** catalogNumber: ZJU 5727.1-2; recordedBy: Xuedi Li; individualCount: 3; sex: female; lifeStage: adult; associatedOccurrences: Pentatomidae; **Taxon:** scientificName: Trissolcus japonicus; order: Hymenoptera; family: Scelionidae; genus: Trissolcus; specificEpithet: japonicus; vernacularName: samurai wasp; **Location:** country: China; stateProvince: Zhejiang; county: Hangzhou; locationRemarks: label transliteration: "Zhejiang, Hangzhou, ex. from eggs of Pentatomidae, 24.v.1957, Xuedi Li"; [浙江，杭州，1957.v.24,李学迪；寄主：蝽象卵]; **Identification:** identifiedBy: Elijah J. Talamas, Huayan Chen; dateIdentified: 2019; **Event:** samplingProtocol: none specified; eventDate: 05/24/1957; **Record Level:** modified: 04/29/2020; language: en
- **Type status:** Other material. **Occurrence:** catalogNumber: ZJU 5727.1-3; recordedBy: Xuedi Li; individualCount: 3; sex: female; lifeStage: adult; associatedOccurrences: Pentatomidae; **Taxon:** scientificName: Trissolcus japonicus; order: Hymenoptera; family: Scelionidae; genus: Trissolcus; specificEpithet: japonicus; vernacularName: samurai wasp; **Location:** country: China; stateProvince: Zhejiang; county: Hangzhou; locationRemarks: label transliteration: "Zhejiang, Hangzhou, ex. from eggs of Pentatomidae, 24.v.1957, Xuedi Li"; [浙江，杭州，1957.v.24,李学迪；寄主：蝽象卵]; **Identification:** identifiedBy: Elijah J. Talamas, Huayan Chen; dateIdentified: 2019; **Event:** samplingProtocol: none specified; eventDate: 05/24/1957; **Record Level:** modified: 04/29/2020; language: en
- **Type status:** Other material. **Occurrence:** catalogNumber: ZJU 5727.1-4; recordedBy: Xuedi Li; individualCount: 3; sex: female; lifeStage: adult; associatedOccurrences: Pentatomidae; **Taxon:** scientificName: Trissolcus japonicus; order: Hymenoptera; family: Scelionidae; genus: Trissolcus; specificEpithet: japonicus; vernacularName: samurai wasp; **Location:** country: China; stateProvince: Zhejiang; county: Hangzhou; locationRemarks: label transliteration: "Zhejiang, Hangzhou, ex. from eggs of Pentatomidae, 24.v.1957, Xuedi Li"; [浙江，杭州，1957.v.24,李学迪；寄主：蝽象卵]; **Identification:** identifiedBy: Elijah J. Talamas, Huayan Chen; dateIdentified: 2019; **Event:** samplingProtocol: none specified; eventDate: 05/24/1957; **Record Level:** modified: 04/29/2020; language: en
- **Type status:** Other material. **Occurrence:** catalogNumber: ZJU 5727.1-5; recordedBy: Xuedi Li; individualCount: 3; sex: female; lifeStage: adult; associatedOccurrences: Pentatomidae; **Taxon:** scientificName: Trissolcus japonicus; order: Hymenoptera; family: Scelionidae; genus: Trissolcus; specificEpithet: japonicus; vernacularName: samurai wasp; **Location:** country: China; stateProvince: Zhejiang; county: Hangzhou; locationRemarks: label transliteration: "Zhejiang, Hangzhou, ex. from eggs of Pentatomidae, 24.v.1957, Xuedi Li"; [浙江，杭州，1957.v.24,李学迪；寄主：蝽象卵]; **Identification:** identifiedBy: Elijah J. Talamas, Huayan Chen; dateIdentified: 2019; **Event:** samplingProtocol: none specified; eventDate: 05/24/1957; **Record Level:** modified: 04/29/2020; language: en
- **Type status:** Other material. **Occurrence:** catalogNumber: ZJU 760779; recordedBy: Yizhen Luo; individualCount: 1; sex: female; lifeStage: adult; associatedOccurrences: Halyomorpha halys; **Taxon:** scientificName: Trissolcus japonicus; order: Hymenoptera; family: Scelionidae; genus: Trissolcus; specificEpithet: japonicus; vernacularName: samurai wasp; **Location:** country: China; stateProvince: Shandong; county: Qingdao; locationRemarks: label transliteration: "Hunan, Changsha, Wangcheng District, ex. from eggs of Niphe elongata , 22.vii.1954, Songyun Mai"; [湖南，长沙望城，1954.vii.22,麦松云；寄主：褐蝽象卵Niphe elongata]; **Identification:** identifiedBy: Elijah J. Talamas, Huayan Chen; dateIdentified: 2019; **Event:** samplingProtocol: none specified; eventDate: 12/22/1950; **Record Level:** modified: 04/29/2020; language: en
- **Type status:** Other material. **Occurrence:** catalogNumber: ZJU 965035-1; recordedBy: Junhua He; individualCount: 2; sex: 1 female，1 male; lifeStage: adult; associatedOccurrences: Pentatomidae; **Taxon:** scientificName: Trissolcus japonicus; order: Hymenoptera; family: Scelionidae; genus: Trissolcus; specificEpithet: japonicus; vernacularName: samurai wasp; **Location:** country: China; stateProvince: Zhejiang; county: Deqing; locationRemarks: label transliteration: “Zhejiang, Deqing County, ex. from eggs of Pentatomidae, 27.v.1995, Junhua He"; [浙江，德清，1995.v.27,何俊华；寄主：蝽象卵]; **Identification:** identifiedBy: Elijah J. Talamas, Huayan Chen; dateIdentified: 2019; **Event:** samplingProtocol: none specified; eventDate: 05/27/1995; **Record Level:** modified: 04/29/2020; language: en
- **Type status:** Other material. **Occurrence:** catalogNumber: ZJU 965035-2; recordedBy: Junhua He; individualCount: 2; sex: female; lifeStage: adult; associatedOccurrences: Pentatomidae; **Taxon:** scientificName: Trissolcus japonicus; order: Hymenoptera; family: Scelionidae; genus: Trissolcus; specificEpithet: japonicus; vernacularName: samurai wasp; **Location:** country: China; stateProvince: Zhejiang; county: Deqing; locationRemarks: label transliteration: “Zhejiang, Deqing County, ex. from eggs of Pentatomidae, 27.v.1995, Junhua He"; [浙江，德清，1995.v.27,何俊华；寄主：蝽象卵]; **Identification:** identifiedBy: Elijah J. Talamas, Huayan Chen; dateIdentified: 2019; **Event:** samplingProtocol: none specified; eventDate: 05/27/1995; **Record Level:** modified: 04/29/2020; language: en
- **Type status:** Other material. **Occurrence:** catalogNumber: ZJU 965035-3; recordedBy: Junhua He; individualCount: 1; sex: female，egg mass; lifeStage: adult; associatedOccurrences: Pentatomidae; **Taxon:** scientificName: Trissolcus japonicus; order: Hymenoptera; family: Scelionidae; genus: Trissolcus; specificEpithet: japonicus; vernacularName: samurai wasp; **Location:** country: China; stateProvince: Zhejiang; county: Deqing; locationRemarks: label transliteration: “Zhejiang, Deqing County, ex. from eggs of Pentatomidae, 27.v.1995, Junhua He"; [浙江，德清，1995.v.27,何俊华；寄主：蝽象卵]; **Identification:** identifiedBy: Elijah J. Talamas, Huayan Chen; dateIdentified: 2019; **Event:** samplingProtocol: none specified; eventDate: 05/27/1995; **Record Level:** modified: 04/29/2020; language: en
- **Type status:** Other material. **Occurrence:** catalogNumber: ZJU 965035-4; recordedBy: Junhua He; individualCount: 2; sex: female; lifeStage: adult; associatedOccurrences: Pentatomidae; **Taxon:** scientificName: Trissolcus japonicus; order: Hymenoptera; family: Scelionidae; genus: Trissolcus; specificEpithet: japonicus; vernacularName: samurai wasp; **Location:** country: China; stateProvince: Zhejiang; county: Deqing; locationRemarks: label transliteration: “Zhejiang, Deqing County, ex. from eggs of Pentatomidae, 27.v.1995, Junhua He"; [浙江，德清，1995.v.27,何俊华；寄主：蝽象卵]; **Identification:** identifiedBy: Elijah J. Talamas, Huayan Chen; dateIdentified: 2019; **Event:** samplingProtocol: none specified; eventDate: 05/27/1995; **Record Level:** modified: 04/29/2020; language: en

#### Distribution

China (Gansu, Shandong, Zhejiang, Taiwan); Italy, Japan, Russia, South Korea, Switzerland and the United States (Talamas et al. 2017, Sabbatini Peverieri et al. 2018, Stahl et al. 2018).

#### Biology

**Host associations.** This species has previously been recorded emerging from the eggs of *Halyomorpha halys* (Stål) in China (Yang et al. 2009). In this study, we found that this species has also been reared from the eggs of *Erthesina fullo* (Thunberg) (Pentatomidae), *Rhaphigaster nebulosa* (Poda) (Pentatomidae) and some other unidentified species of Pentatomidae.

#### Images

*Trissolcus japonicus* Ashmead 1904 (Fig. 3).

### Trissolcus latisulcus

(Crawford) 1913

3480799C-6921-5F5B-B2E5-CECDBC2EF617

#### Materials

- **Type status:** Other material. **Occurrence:** catalogNumber: ZJU 835240-1; recordedBy: Jiasheng Gan; individualCount: 1; sex: male; lifeStage: adult; associatedOccurrences: Poecilocoris latus; **Taxon:** scientificName: Trissolcus latisulcus; order: Hymenoptera; family: Scelionidae; genus: Trissolcus; specificEpithet: latisulcus; **Location:** country: China; stateProvince: Yunnan; county: Guangnan; locationRemarks: label transliteration: "Yunnan, Guangnan County, ex. from eggs of Poecilocoris latus, viii.1980, Jiasheng Gan"; [云南，广南，1980.viii, 甘家生；寄主：油茶蝽卵 Poecilocoris latus]; **Identification:** identifiedBy: Elijah J. Talamas, Huayan Chen; dateIdentified: 2019; **Event:** samplingProtocol: none specified; eventDate: 1980-08; **Record Level:** modified: 04/29/2020; language: en
- **Type status:** Other material. **Occurrence:** catalogNumber: ZJU 835240-2; recordedBy: Jiasheng Gan; individualCount: 1; sex: female; lifeStage: adult; associatedOccurrences: Poecilocoris latus; **Taxon:** scientificName: Trissolcus latisulcus; order: Hymenoptera; family: Scelionidae; genus: Trissolcus; specificEpithet: latisulcus; **Location:** country: China; stateProvince: Yunnan; county: Guangnan; locationRemarks: label transliteration: "Yunnan, Guangnan County, ex. from eggs of Poecilocoris latus, viii.1980, Jiasheng Gan"; [云南，广南，1980.viii, 甘家生；寄主：油茶蝽卵 Poecilocoris latus]; **Identification:** identifiedBy: Elijah J. Talamas, Huayan Chen; dateIdentified: 2019; **Event:** samplingProtocol: none specified; eventDate: 1980-08; **Record Level:** modified: 04/29/2020; language: en
- **Type status:** Other material. **Occurrence:** catalogNumber: ZJU 835240-3; recordedBy: Jiasheng Gan; individualCount: 1; sex: female; lifeStage: adult; associatedOccurrences: Poecilocoris latus; **Taxon:** scientificName: Trissolcus latisulcus; order: Hymenoptera; family: Scelionidae; genus: Trissolcus; specificEpithet: latisulcus; **Location:** country: China; stateProvince: Yunnan; county: Guangnan; locationRemarks: label transliteration: "Yunnan, Guangnan County, ex. from eggs of Poecilocoris latus, viii.1980, Jiasheng Gan"; [云南，广南，1980.viii, 甘家生；寄主：油茶蝽卵 Poecilocoris latus]; **Identification:** identifiedBy: Elijah J. Talamas, Huayan Chen; dateIdentified: 2019; **Event:** samplingProtocol: none specified; eventDate: 1980-08; **Record Level:** modified: 04/29/2020; language: en
- **Type status:** Other material. **Occurrence:** catalogNumber: ZJU 835240-4; recordedBy: Jiasheng Gan; individualCount: 1; sex: female; lifeStage: adult; associatedOccurrences: Poecilocoris latus; **Taxon:** scientificName: Trissolcus latisulcus; order: Hymenoptera; family: Scelionidae; genus: Trissolcus; specificEpithet: latisulcus; **Location:** country: China; stateProvince: Yunnan; county: Guangnan; locationRemarks: label transliteration: "Yunnan, Guangnan County, ex. from eggs of Poecilocoris latus, viii.1980, Jiasheng Gan"; [云南，广南，1980.viii, 甘家生；寄主：油茶蝽卵 Poecilocoris latus]; **Identification:** identifiedBy: Elijah J. Talamas, Huayan Chen; dateIdentified: 2019; **Event:** samplingProtocol: none specified; eventDate: 1980-08; **Record Level:** modified: 04/29/2020; language: en
- **Type status:** Other material. **Occurrence:** catalogNumber: ZJU 835240-5; recordedBy: Jiasheng Gan; individualCount: 1; sex: female; lifeStage: adult; associatedOccurrences: Poecilocoris latus; **Taxon:** scientificName: Trissolcus latisulcus; order: Hymenoptera; family: Scelionidae; genus: Trissolcus; specificEpithet: latisulcus; **Location:** country: China; stateProvince: Yunnan; county: Guangnan; locationRemarks: label transliteration: "Yunnan, Guangnan County, ex. from eggs of Poecilocoris latus, viii.1980, Jiasheng Gan"; [云南，广南，1980.viii, 甘家生；寄主：油茶蝽卵 Poecilocoris latus]; **Identification:** identifiedBy: Elijah J. Talamas, Huayan Chen; dateIdentified: 2019; **Event:** samplingProtocol: none specified; eventDate: 1980-08; **Record Level:** modified: 04/29/2020; language: en
- **Type status:** Other material. **Occurrence:** catalogNumber: ZJU 835240-6; recordedBy: Jiasheng Gan; individualCount: 1; sex: female; lifeStage: adult; associatedOccurrences: Poecilocoris latus; **Taxon:** scientificName: Trissolcus latisulcus; order: Hymenoptera; family: Scelionidae; genus: Trissolcus; specificEpithet: latisulcus; **Location:** country: China; stateProvince: Yunnan; county: Guangnan; locationRemarks: label transliteration: "Yunnan, Guangnan County, ex. from eggs of Poecilocoris latus, viii.1980, Jiasheng Gan"; [云南，广南，1980.viii, 甘家生；寄主：油茶蝽卵 Poecilocoris latus]; **Identification:** identifiedBy: Elijah J. Talamas, Huayan Chen; dateIdentified: 2019; **Event:** samplingProtocol: none specified; eventDate: 1980-08; **Record Level:** modified: 04/29/2020; language: en
- **Type status:** Other material. **Occurrence:** catalogNumber: ZJU 835240-7; recordedBy: Jiasheng Gan; individualCount: 1; sex: male; lifeStage: adult; associatedOccurrences: Poecilocoris latus; **Taxon:** scientificName: Trissolcus latisulcus; order: Hymenoptera; family: Scelionidae; genus: Trissolcus; specificEpithet: latisulcus; **Location:** country: China; stateProvince: Yunnan; county: Guangnan; locationRemarks: label transliteration: "Yunnan, Guangnan County, ex. from eggs of Poecilocoris latus, viii.1980, Jiasheng Gan"; [云南，广南，1980.viii, 甘家生；寄主：油茶蝽卵 Poecilocoris latus]; **Identification:** identifiedBy: Elijah J. Talamas, Huayan Chen; dateIdentified: 2019; **Event:** samplingProtocol: none specified; eventDate: 1980-08; **Record Level:** modified: 04/29/2020; language: en
- **Type status:** Other material. **Occurrence:** catalogNumber: ZJU 835240-8; recordedBy: Jiasheng Gan; individualCount: 1; sex: female; lifeStage: adult; associatedOccurrences: Poecilocoris latus; **Taxon:** scientificName: Trissolcus latisulcus; order: Hymenoptera; family: Scelionidae; genus: Trissolcus; specificEpithet: latisulcus; **Location:** country: China; stateProvince: Yunnan; county: Guangnan; locationRemarks: label transliteration: "Yunnan, Guangnan County, ex. from eggs of Poecilocoris latus, viii.1980, Jiasheng Gan"; [云南，广南，1980.viii, 甘家生；寄主：油茶蝽卵 Poecilocoris latus]; **Identification:** identifiedBy: Elijah J. Talamas, Huayan Chen; dateIdentified: 2019; **Event:** samplingProtocol: none specified; eventDate: 1980-08; **Record Level:** modified: 04/29/2020; language: en
- **Type status:** Other material. **Occurrence:** catalogNumber: ZJU 835240-9; recordedBy: Jiasheng Gan; individualCount: 1; sex: female; lifeStage: adult; associatedOccurrences: Poecilocoris latus; **Taxon:** scientificName: Trissolcus latisulcus; order: Hymenoptera; family: Scelionidae; genus: Trissolcus; specificEpithet: latisulcus; **Location:** country: China; stateProvince: Yunnan; county: Guangnan; locationRemarks: label transliteration: "Yunnan, Guangnan County, ex. from eggs of Poecilocoris latus, viii.1980, Jiasheng Gan"; [云南，广南，1980.viii, 甘家生；寄主：油茶蝽卵 Poecilocoris latus]; **Identification:** identifiedBy: Elijah J. Talamas, Huayan Chen; dateIdentified: 2019; **Event:** samplingProtocol: none specified; eventDate: 1980-08; **Record Level:** modified: 04/29/2020; language: en
- **Type status:** Other material. **Occurrence:** catalogNumber: ZJU 835240-10; recordedBy: Jiasheng Gan; individualCount: 1; sex: male; lifeStage: adult; associatedOccurrences: Poecilocoris latus; **Taxon:** scientificName: Trissolcus latisulcus; order: Hymenoptera; family: Scelionidae; genus: Trissolcus; specificEpithet: latisulcus; **Location:** country: China; stateProvince: Yunnan; county: Guangnan; locationRemarks: label transliteration: "Yunnan, Guangnan County, ex. from eggs of Poecilocoris latus, viii.1980, Jiasheng Gan"; [云南，广南，1980.viii, 甘家生；寄主：油茶蝽卵 Poecilocoris latus]; **Identification:** identifiedBy: Elijah J. Talamas, Huayan Chen; dateIdentified: 2019; **Event:** samplingProtocol: none specified; eventDate: 1980-08; **Record Level:** modified: 04/29/2020; language: en
- **Type status:** Other material. **Occurrence:** catalogNumber: ZJU 835240-11; recordedBy: Jiasheng Gan; individualCount: 1; sex: female; lifeStage: adult; associatedOccurrences: Poecilocoris latus; **Taxon:** scientificName: Trissolcus latisulcus; order: Hymenoptera; family: Scelionidae; genus: Trissolcus; specificEpithet: latisulcus; **Location:** country: China; stateProvince: Yunnan; county: Guangnan; locationRemarks: label transliteration: "Yunnan, Guangnan County, ex. from eggs of Poecilocoris latus, viii.1980, Jiasheng Gan"; [云南，广南，1980.viii, 甘家生；寄主：油茶蝽卵 Poecilocoris latus]; **Identification:** identifiedBy: Elijah J. Talamas, Huayan Chen; dateIdentified: 2019; **Event:** samplingProtocol: none specified; eventDate: 1980-08; **Record Level:** modified: 04/29/2020; language: en
- **Type status:** Other material. **Occurrence:** catalogNumber: ZJU 835240-12; recordedBy: Jiasheng Gan; individualCount: 1; sex: female; lifeStage: adult; associatedOccurrences: Poecilocoris latus; **Taxon:** scientificName: Trissolcus latisulcus; order: Hymenoptera; family: Scelionidae; genus: Trissolcus; specificEpithet: latisulcus; **Location:** country: China; stateProvince: Yunnan; county: Guangnan; locationRemarks: label transliteration: "Yunnan, Guangnan County, ex. from eggs of Poecilocoris latus, viii.1980, Jiasheng Gan"; [云南，广南，1980.viii, 甘家生；寄主：油茶蝽卵 Poecilocoris latus]; **Identification:** identifiedBy: Elijah J. Talamas, Huayan Chen; dateIdentified: 2019; **Event:** samplingProtocol: none specified; eventDate: 1980-08; **Record Level:** modified: 04/29/2020; language: en
- **Type status:** Other material. **Occurrence:** catalogNumber: ZJU 835240-13; recordedBy: Jiasheng Gan; individualCount: 1; sex: female; lifeStage: adult; associatedOccurrences: Poecilocoris latus; **Taxon:** scientificName: Trissolcus latisulcus; order: Hymenoptera; family: Scelionidae; genus: Trissolcus; specificEpithet: latisulcus; **Location:** country: China; stateProvince: Yunnan; county: Guangnan; locationRemarks: label transliteration: "Yunnan, Guangnan County, ex. from eggs of Poecilocoris latus, viii.1980, Jiasheng Gan"; [云南，广南，1980.viii, 甘家生；寄主：油茶蝽卵 Poecilocoris latus]; **Identification:** identifiedBy: Elijah J. Talamas, Huayan Chen; dateIdentified: 2019; **Event:** samplingProtocol: none specified; eventDate: 1980-08; **Record Level:** modified: 04/29/2020; language: en
- **Type status:** Other material. **Occurrence:** catalogNumber: ZJU 835240-14; recordedBy: Jiasheng Gan; individualCount: 1; sex: female; lifeStage: adult; associatedOccurrences: Poecilocoris latus; **Taxon:** scientificName: Trissolcus latisulcus; order: Hymenoptera; family: Scelionidae; genus: Trissolcus; specificEpithet: latisulcus; **Location:** country: China; stateProvince: Yunnan; county: Guangnan; locationRemarks: label transliteration: "Yunnan, Guangnan County, ex. from eggs of Poecilocoris latus, viii.1980, Jiasheng Gan"; [云南，广南，1980.viii, 甘家生；寄主：油茶蝽卵 Poecilocoris latus]; **Identification:** identifiedBy: Elijah J. Talamas, Huayan Chen; dateIdentified: 2019; **Event:** samplingProtocol: none specified; eventDate: 1980-08; **Record Level:** modified: 04/29/2020; language: en
- **Type status:** Other material. **Occurrence:** catalogNumber: ZJU 835240-15; recordedBy: Jiasheng Gan; individualCount: 1; sex: female; lifeStage: adult; associatedOccurrences: Poecilocoris latus; **Taxon:** scientificName: Trissolcus latisulcus; order: Hymenoptera; family: Scelionidae; genus: Trissolcus; specificEpithet: latisulcus; **Location:** country: China; stateProvince: Yunnan; county: Guangnan; locationRemarks: label transliteration: "Yunnan, Guangnan County, ex. from eggs of Poecilocoris latus, viii.1980, Jiasheng Gan"; [云南，广南，1980.viii, 甘家生；寄主：油茶蝽卵 Poecilocoris latus]; **Identification:** identifiedBy: Elijah J. Talamas, Huayan Chen; dateIdentified: 2019; **Event:** samplingProtocol: none specified; eventDate: 1980-08; **Record Level:** modified: 04/29/2020; language: en
- **Type status:** Other material. **Occurrence:** catalogNumber: ZJU 835240-16; recordedBy: Jiasheng Gan; individualCount: 1; sex: male; lifeStage: adult; associatedOccurrences: Poecilocoris latus; **Taxon:** scientificName: Trissolcus latisulcus; order: Hymenoptera; family: Scelionidae; genus: Trissolcus; specificEpithet: latisulcus; **Location:** country: China; stateProvince: Yunnan; county: Guangnan; locationRemarks: label transliteration: "Yunnan, Guangnan County, ex. from eggs of Poecilocoris latus, viii.1980, Jiasheng Gan"; [云南，广南，1980.viii, 甘家生；寄主：油茶蝽卵 Poecilocoris latus]; **Identification:** identifiedBy: Elijah J. Talamas, Huayan Chen; dateIdentified: 2019; **Event:** samplingProtocol: none specified; eventDate: 1980-08; **Record Level:** modified: 04/29/2020; language: en
- **Type status:** Other material. **Occurrence:** catalogNumber: ZJU 835240-17; recordedBy: Jiasheng Gan; individualCount: 1; sex: female; lifeStage: adult; associatedOccurrences: Poecilocoris latus; **Taxon:** scientificName: Trissolcus latisulcus; order: Hymenoptera; family: Scelionidae; genus: Trissolcus; specificEpithet: latisulcus; **Location:** country: China; stateProvince: Yunnan; county: Guangnan; locationRemarks: label transliteration: "Yunnan, Guangnan County, ex. from eggs of Poecilocoris latus, viii.1980, Jiasheng Gan"; [云南，广南，1980.viii, 甘家生；寄主：油茶蝽卵 Poecilocoris latus]; **Identification:** identifiedBy: Elijah J. Talamas, Huayan Chen; dateIdentified: 2019; **Event:** samplingProtocol: none specified; eventDate: 1980-08; **Record Level:** modified: 04/29/2020; language: en
- **Type status:** Other material. **Occurrence:** catalogNumber: ZJU 835240-18; recordedBy: Jiasheng Gan; individualCount: 1; sex: male; lifeStage: adult; associatedOccurrences: Poecilocoris latus; **Taxon:** scientificName: Trissolcus latisulcus; order: Hymenoptera; family: Scelionidae; genus: Trissolcus; specificEpithet: latisulcus; **Location:** country: China; stateProvince: Yunnan; county: Guangnan; locationRemarks: label transliteration: "Yunnan, Guangnan County, ex. from eggs of Poecilocoris latus, viii.1980, Jiasheng Gan"; [云南，广南，1980.viii, 甘家生；寄主：油茶蝽卵 Poecilocoris latus]; **Identification:** identifiedBy: Elijah J. Talamas, Huayan Chen; dateIdentified: 2019; **Event:** samplingProtocol: none specified; eventDate: 1980-08; **Record Level:** modified: 04/29/2020; language: en

#### Distribution

China (Yunnan, Taiwan); India, Laos, Malaysia, Philippines, Thailand and Vietnam (Talamas et al. 2017).

#### Biology

**Host associations.** This species was reared from the eggs of *Poecilocoris latus* Dallas (Scutelleridae) in China.

#### Notes

Gan 1982 reported an unidentified species of Scelionidae attacking the eggs of *P. latus* feeding on *Camellia* trees from Yunnan Province. We examined the specimens that formed the basis of his report and we now know the species is *T. latisulcus.* According to *Gan 1982*, *T. latisulcus* could lay up to 700 eggs under laboratory conditions. There are 6–7 generations of *T. latisulcus* per year in Guangnan County of Yunnan, with a generation time of 20–30 days and they overwinter as adults.

#### Images

Trissolcus latisulcus Crawford 1913 (*Fig. 4).

### Trissolcus mitsukurii

(Ashmead) 1904

813BD985-AD25-55BB-A6F0-39A60CCFADEF

#### Materials

- **Type status:** Other material. **Occurrence:** catalogNumber: ZJU 740644-1; recordedBy: Zhengxiang Ye; individualCount: 1; sex: female; lifeStage: adult; associatedOccurrences: Pentatomidae; **Taxon:** scientificName: Trissolcus mitsukurii; order: Hymenoptera; family: Scelionidae; genus: Trissolcus; specificEpithet: mitsukurii; **Location:** country: China; stateProvince: Jiangxi; county: Nanchang; locationRemarks: label transliteration: "Jiangxi, Nanchang, ex. from eggs of Pentatomidae，19，Zhengxiang Ye"; [江西，南昌，19，叶正襄；寄主：蝽蟓卵]; **Identification:** identifiedBy: Elijah J. Talamas, Huayan Chen; dateIdentified: 2019; **Event:** samplingProtocol: none specified; **Record Level:** modified: 04/29/2020; language: en; collectionCode: Insects; basisOfRecord: PreservedSpecimen
- **Type status:** Other material. **Occurrence:** catalogNumber: ZJU 740644-2; recordedBy: Zhengxiang Ye; individualCount: 1; sex: female; lifeStage: adult; associatedOccurrences: Pentatomidae; **Taxon:** scientificName: Trissolcus mitsukurii; order: Hymenoptera; family: Scelionidae; genus: Trissolcus; specificEpithet: mitsukurii; **Location:** country: China; stateProvince: Jiangxi; county: Nanchang; locationRemarks: label transliteration: "Jiangxi, Nanchang, ex. from eggs of Pentatomidae，19，Zhengxiang Ye"; [江西，南昌，19，叶正襄；寄主：蝽蟓卵]; **Identification:** identifiedBy: Elijah J. Talamas, Huayan Chen; dateIdentified: 2019; **Event:** samplingProtocol: none specified; **Record Level:** modified: 04/29/2020; language: en; collectionCode: Insects; basisOfRecord: PreservedSpecimen
- **Type status:** Other material. **Occurrence:** catalogNumber: ZJU 740644-3; recordedBy: Zhengxiang Ye; individualCount: 1; sex: female; lifeStage: adult; associatedOccurrences: Pentatomidae; **Taxon:** scientificName: Trissolcus mitsukurii; order: Hymenoptera; family: Scelionidae; genus: Trissolcus; specificEpithet: mitsukurii; **Location:** country: China; stateProvince: Jiangxi; county: Nanchang; locationRemarks: label transliteration: "Jiangxi, Nanchang, ex. from eggs of Pentatomidae，19，Zhengxiang Ye"; [江西，南昌，19，叶正襄；寄主：蝽蟓卵]; **Identification:** identifiedBy: Elijah J. Talamas, Huayan Chen; dateIdentified: 2019; **Event:** samplingProtocol: none specified; **Record Level:** modified: 04/29/2020; language: en; collectionCode: Insects; basisOfRecord: PreservedSpecimen
- **Type status:** Other material. **Occurrence:** catalogNumber: ZJU 740644-4; recordedBy: Zhengxiang Ye; individualCount: 1; sex: female; lifeStage: adult; associatedOccurrences: Pentatomidae; **Taxon:** scientificName: Trissolcus mitsukurii; order: Hymenoptera; family: Scelionidae; genus: Trissolcus; specificEpithet: mitsukurii; **Location:** country: China; stateProvince: Jiangxi; county: Nanchang; locationRemarks: label transliteration: "Jiangxi, Nanchang, ex. from eggs of Pentatomidae，19，Zhengxiang Ye"; [江西，南昌，19，叶正襄；寄主：蝽蟓卵]; **Identification:** identifiedBy: Elijah J. Talamas, Huayan Chen; dateIdentified: 2019; **Event:** samplingProtocol: none specified; **Record Level:** modified: 04/29/2020; language: en; collectionCode: Insects; basisOfRecord: PreservedSpecimen
- **Type status:** Other material. **Occurrence:** catalogNumber: ZJU 740644-5; recordedBy: Zhengxiang Ye; individualCount: 1; sex: female; lifeStage: adult; associatedOccurrences: Pentatomidae; **Taxon:** scientificName: Trissolcus mitsukurii; order: Hymenoptera; family: Scelionidae; genus: Trissolcus; specificEpithet: mitsukurii; **Location:** country: China; stateProvince: Jiangxi; county: Nanchang; locationRemarks: label transliteration: "Jiangxi, Nanchang, ex. from eggs of Pentatomidae，19，Zhengxiang Ye"; [江西，南昌，19，叶正襄；寄主：蝽蟓卵]; **Identification:** identifiedBy: Elijah J. Talamas, Huayan Chen; dateIdentified: 2019; **Event:** samplingProtocol: none specified; **Record Level:** modified: 04/29/2020; language: en; collectionCode: Insects; basisOfRecord: PreservedSpecimen
- **Type status:** Other material. **Occurrence:** catalogNumber: ZJU 870488-1; recordedBy: Hanlin Chen; individualCount: 1; sex: female; lifeStage: adult; associatedOccurrences: Pentatomidae; **Taxon:** scientificName: Trissolcus mitsukurii; order: Hymenoptera; family: Scelionidae; genus: Trissolcus; specificEpithet: mitsukurii; **Location:** country: China; stateProvince: Zhejiang; county: Songyang; locationRemarks: label transliteration: "Zhejiang, Songyang County, ex. from eggs of Pentatomidae, ix.1985, Hanlin Chen"; [浙江，松阳，1985.ix, 陈汉林；寄主：蝽卵]; **Identification:** identifiedBy: Elijah J. Talamas, Huayan Chen; dateIdentified: 2019; **Event:** samplingProtocol: none specified; eventDate: 1985-11; **Record Level:** modified: 04/29/2020; language: en; collectionCode: Insects; basisOfRecord: PreservedSpecimen
- **Type status:** Other material. **Occurrence:** catalogNumber: ZJU 870488-2; recordedBy: Hanlin Chen; individualCount: 1; sex: female; lifeStage: adult; associatedOccurrences: Pentatomidae; **Taxon:** scientificName: Trissolcus mitsukurii; order: Hymenoptera; family: Scelionidae; genus: Trissolcus; specificEpithet: mitsukurii; **Location:** country: China; stateProvince: Zhejiang; county: Songyang; locationRemarks: label transliteration: "Zhejiang, Songyang County, ex. from eggs of Pentatomidae, ix.1985, Hanlin Chen"; [浙江，松阳，1985.ix, 陈汉林；寄主：蝽卵]; **Identification:** identifiedBy: Elijah J. Talamas, Huayan Chen; dateIdentified: 2019; **Event:** samplingProtocol: none specified; eventDate: 1985-11; **Record Level:** modified: 04/29/2020; language: en; collectionCode: Insects; basisOfRecord: PreservedSpecimen
- **Type status:** Other material. **Occurrence:** catalogNumber: ZJU 870488-3; recordedBy: Hanlin Chen; individualCount: 1; sex: female; lifeStage: adult; associatedOccurrences: Pentatomidae; **Taxon:** scientificName: Trissolcus mitsukurii; order: Hymenoptera; family: Scelionidae; genus: Trissolcus; specificEpithet: mitsukurii; **Location:** country: China; stateProvince: Zhejiang; county: Songyang; locationRemarks: label transliteration: "Zhejiang, Songyang County, ex. from eggs of Pentatomidae, ix.1985, Hanlin Chen"; [浙江，松阳，1985.ix, 陈汉林；寄主：蝽卵]; **Identification:** identifiedBy: Elijah J. Talamas, Huayan Chen; dateIdentified: 2019; **Event:** samplingProtocol: none specified; eventDate: 1985-11; **Record Level:** modified: 04/29/2020; language: en; collectionCode: Insects; basisOfRecord: PreservedSpecimen
- **Type status:** Other material. **Occurrence:** catalogNumber: ZJU 870488-4; recordedBy: Hanlin Chen; individualCount: 1; sex: female; lifeStage: adult; associatedOccurrences: Pentatomidae; **Taxon:** scientificName: Trissolcus mitsukurii; order: Hymenoptera; family: Scelionidae; genus: Trissolcus; specificEpithet: mitsukurii; **Location:** country: China; stateProvince: Zhejiang; county: Songyang; locationRemarks: label transliteration: "Zhejiang, Songyang County, ex. from eggs of Pentatomidae, ix.1985, Hanlin Chen"; [浙江，松阳，1985.ix, 陈汉林；寄主：蝽卵]; **Identification:** identifiedBy: Elijah J. Talamas, Huayan Chen; dateIdentified: 2019; **Event:** samplingProtocol: none specified; eventDate: 1985-11; **Record Level:** modified: 04/29/2020; language: en; collectionCode: Insects; basisOfRecord: PreservedSpecimen
- **Type status:** Other material. **Occurrence:** catalogNumber: ZJU 870488-5; recordedBy: Hanlin Chen; individualCount: 1; sex: female; lifeStage: adult; associatedOccurrences: Pentatomidae; **Taxon:** scientificName: Trissolcus mitsukurii; order: Hymenoptera; family: Scelionidae; genus: Trissolcus; specificEpithet: mitsukurii; **Location:** country: China; stateProvince: Zhejiang; county: Songyang; locationRemarks: label transliteration: "Zhejiang, Songyang County, ex. from eggs of Pentatomidae, ix.1985, Hanlin Chen"; [浙江，松阳，1985.ix, 陈汉林；寄主：蝽卵]; **Identification:** identifiedBy: Elijah J. Talamas, Huayan Chen; dateIdentified: 2019; **Event:** samplingProtocol: none specified; eventDate: 1985-11; **Record Level:** modified: 04/29/2020; language: en; collectionCode: Insects; basisOfRecord: PreservedSpecimen
- **Type status:** Other material. **Occurrence:** catalogNumber: ZJU 816020-1; recordedBy: Dongxiang Xie; individualCount: 1; sex: female; lifeStage: adult; associatedOccurrences: Pentatomidae; **Taxon:** scientificName: Trissolcus mitsukurii; order: Hymenoptera; family: Scelionidae; genus: Trissolcus; specificEpithet: mitsukurii; **Location:** country: China; stateProvince: Guizhou; county: Sandu; locationRemarks: label transliteration: "Guizhou, Sandu Shui Autonomous County, ex. from eggs of Pentatomidae, v.1981, Dongxiang Xie"; [贵州，三都，1981.v, 谢冬香，寄主：蝽蟓卵]; **Identification:** identifiedBy: Elijah J. Talamas, Huayan Chen; dateIdentified: 2019; **Event:** samplingProtocol: none specified; eventDate: 1981-05; **Record Level:** modified: 04/29/2020; language: en
- **Type status:** Other material. **Occurrence:** catalogNumber: ZJU 816020-2; recordedBy: Dongxiang Xie; individualCount: 1; sex: female; lifeStage: adult; associatedOccurrences: Pentatomidae; **Taxon:** scientificName: Trissolcus mitsukurii; order: Hymenoptera; family: Scelionidae; genus: Trissolcus; specificEpithet: mitsukurii; **Location:** country: China; stateProvince: Guizhou; county: Sandu; locationRemarks: label transliteration: "Guizhou, Sandu Shui Autonomous County, ex. from eggs of Pentatomidae, v.1981, Dongxiang Xie"; [贵州，三都，1981.v, 谢冬香，寄主：蝽蟓卵]; **Identification:** identifiedBy: Elijah J. Talamas, Huayan Chen; dateIdentified: 2019; **Event:** samplingProtocol: none specified; eventDate: 1981-05; **Record Level:** modified: 04/29/2020; language: en
- **Type status:** Other material. **Occurrence:** catalogNumber: ZJU 816020-3; recordedBy: Dongxiang Xie; individualCount: 1; sex: female; lifeStage: adult; associatedOccurrences: Pentatomidae; **Taxon:** scientificName: Trissolcus mitsukurii; order: Hymenoptera; family: Scelionidae; genus: Trissolcus; specificEpithet: mitsukurii; **Location:** country: China; stateProvince: Guizhou; county: Sandu; locationRemarks: label transliteration: "Guizhou, Sandu Shui Autonomous County, ex. from eggs of Pentatomidae, v.1981, Dongxiang Xie"; [贵州，三都，1981.v, 谢冬香，寄主：蝽蟓卵]; **Identification:** identifiedBy: Elijah J. Talamas, Huayan Chen; dateIdentified: 2019; **Event:** samplingProtocol: none specified; eventDate: 1981-05; **Record Level:** modified: 04/29/2020; language: en
- **Type status:** Other material. **Occurrence:** catalogNumber: ZJU 816020-4; recordedBy: Dongxiang Xie; individualCount: 1; sex: male; lifeStage: adult; associatedOccurrences: Pentatomidae; **Taxon:** scientificName: Trissolcus mitsukurii; order: Hymenoptera; family: Scelionidae; genus: Trissolcus; specificEpithet: mitsukurii; **Location:** country: China; stateProvince: Guizhou; county: Sandu; locationRemarks: label transliteration: "Guizhou, Sandu Shui Autonomous County, ex. from eggs of Pentatomidae, v.1981, Dongxiang Xie"; [贵州，三都，1981.v, 谢冬香，寄主：蝽蟓卵]; **Identification:** identifiedBy: Elijah J. Talamas, Huayan Chen; dateIdentified: 2019; **Event:** samplingProtocol: none specified; eventDate: 1981-05; **Record Level:** modified: 04/29/2020; language: en
- **Type status:** Other material. **Occurrence:** catalogNumber: ZJU 64032.1-1; recordedBy: Dengdi Jin; individualCount: 1; sex: female; lifeStage: adult; associatedOccurrences: Pentatomidae; **Taxon:** scientificName: Trissolcus mitsukurii; order: Hymenoptera; family: Scelionidae; genus: Trissolcus; specificEpithet: mitsukurii; **Location:** country: China; stateProvince: Zhejiang; county: Hangzhou; locationRemarks: label transliteration: "Zhejiang, Hangzhou, ex. from eggs of Pentatomidae, 25.vi.1964, Dengdi Jin"; [浙江，杭州，1964.6.25，金登迪；寄主：蝽象]; **Identification:** identifiedBy: Elijah J. Talamas, Huayan Chen; dateIdentified: 2019; **Event:** samplingProtocol: none specified; eventDate: 06/25/1964; **Record Level:** modified: 04/29/2020; language: en
- **Type status:** Other material. **Occurrence:** catalogNumber: ZJU 64032.1-2; recordedBy: Dengdi Jin; individualCount: 3; sex: female; lifeStage: adult; associatedOccurrences: Pentatomidae; **Taxon:** scientificName: Trissolcus mitsukurii; order: Hymenoptera; family: Scelionidae; genus: Trissolcus; specificEpithet: mitsukurii; **Location:** country: China; stateProvince: Zhejiang; county: Hangzhou; locationRemarks: label transliteration: "Zhejiang, Hangzhou, ex. from eggs of Pentatomidae, 25.vi.1964, Dengdi Jin"; [浙江，杭州，1964.6.25，金登迪；寄主：蝽象]; **Identification:** identifiedBy: Elijah J. Talamas, Huayan Chen; dateIdentified: 2019; **Event:** samplingProtocol: none specified; eventDate: 06/25/1964; **Record Level:** modified: 04/29/2020; language: en
- **Type status:** Other material. **Occurrence:** catalogNumber: ZJU 64032.1-3; recordedBy: Dengdi Jin; individualCount: 3; sex: female; lifeStage: adult; associatedOccurrences: Pentatomidae; **Taxon:** scientificName: Trissolcus mitsukurii; order: Hymenoptera; family: Scelionidae; genus: Trissolcus; specificEpithet: mitsukurii; **Location:** country: China; stateProvince: Zhejiang; county: Hangzhou; locationRemarks: label transliteration: "Zhejiang, Hangzhou, ex. from eggs of Pentatomidae, 25.vi.1964, Dengdi Jin"; [浙江，杭州，1964.6.25，金登迪；寄主：蝽象]; **Identification:** identifiedBy: Elijah J. Talamas, Huayan Chen; dateIdentified: 2019; **Event:** samplingProtocol: none specified; eventDate: 06/25/1964; **Record Level:** modified: 04/29/2020; language: en
- **Type status:** Other material. **Occurrence:** catalogNumber: ZJU 64032.1-4; recordedBy: Dengdi Jin; individualCount: 3; sex: 2 female, 1 male; lifeStage: adult; associatedOccurrences: Pentatomidae; **Taxon:** scientificName: Trissolcus mitsukurii; order: Hymenoptera; family: Scelionidae; genus: Trissolcus; specificEpithet: mitsukurii; **Location:** country: China; stateProvince: Zhejiang; county: Hangzhou; locationRemarks: label transliteration: "Zhejiang, Hangzhou, ex. from eggs of Pentatomidae, 25.vi.1964, Dengdi Jin"; [浙江，杭州，1964.6.25，金登迪；寄主：蝽象]; **Identification:** identifiedBy: Elijah J. Talamas, Huayan Chen; dateIdentified: 2019; **Event:** samplingProtocol: none specified; eventDate: 06/25/1964; **Record Level:** modified: 04/29/2020; language: en
- **Type status:** Other material. **Occurrence:** catalogNumber: ZJU 7501231-1; recordedBy: Zhibang Liu; individualCount: 1; sex: female; lifeStage: adult; associatedOccurrences: Pentatomidae; **Taxon:** scientificName: Trissolcus mitsukurii; order: Hymenoptera; family: Scelionidae; genus: Trissolcus; specificEpithet: mitsukurii; **Location:** country: China; stateProvince: Guizhou; county: Luodian; locationRemarks: label transliteration: "Guizhou, Luodian County, ex. from eggs of Pentatomidae on rice, 1974, Zhibang Liu"; [贵州，罗甸，1974，刘治邦；寄主：水稻上蝽卵]; **Identification:** identifiedBy: Elijah J. Talamas, Huayan Chen; dateIdentified: 2019; **Event:** samplingProtocol: none specified; eventDate: 1974; **Record Level:** modified: 04/29/2020; language: en
- **Type status:** Other material. **Occurrence:** catalogNumber: ZJU 7501231-2; recordedBy: Zhibang Liu; individualCount: 2; sex: male; lifeStage: adult; associatedOccurrences: Pentatomidae; **Taxon:** scientificName: Trissolcus mitsukurii; order: Hymenoptera; family: Scelionidae; genus: Trissolcus; specificEpithet: mitsukurii; **Location:** country: China; stateProvince: Guizhou; county: Luodian; locationRemarks: label transliteration: "Guizhou, Luodian County, ex. from eggs of Pentatomidae on rice, 1974, Zhibang Liu"; [贵州，罗甸，1974，刘治邦；寄主：水稻上蝽卵]; **Identification:** identifiedBy: Elijah J. Talamas, Huayan Chen; dateIdentified: 2019; **Event:** samplingProtocol: none specified; eventDate: 1974; **Record Level:** modified: 04/29/2020; language: en
- **Type status:** Other material. **Occurrence:** catalogNumber: ZJU 7501231-3; recordedBy: Zhibang Liu; individualCount: 2; sex: female; lifeStage: adult; associatedOccurrences: Pentatomidae; **Taxon:** scientificName: Trissolcus mitsukurii; order: Hymenoptera; family: Scelionidae; genus: Trissolcus; specificEpithet: mitsukurii; **Location:** country: China; stateProvince: Guizhou; county: Luodian; locationRemarks: label transliteration: "Guizhou, Luodian County, ex. from eggs of Pentatomidae on rice, 1974, Zhibang Liu"; [贵州，罗甸，1974，刘治邦；寄主：水稻上蝽卵]; **Identification:** identifiedBy: Elijah J. Talamas, Huayan Chen; dateIdentified: 2019; **Event:** samplingProtocol: none specified; eventDate: 1974; **Record Level:** modified: 04/29/2020; language: en
- **Type status:** Other material. **Occurrence:** catalogNumber: ZJU 5737.5; recordedBy: Fahong Yang; individualCount: 2; sex: female; lifeStage: adult; associatedOccurrences: Pentatomidae; **Taxon:** scientificName: Trissolcus mitsukurii; order: Hymenoptera; family: Scelionidae; genus: Trissolcus; specificEpithet: mitsukurii; **Location:** country: China; stateProvince: Zhejiang; county: Hangzhou; locationRemarks: label transliteration: "Zhejiang, Hangzhou, ex. from eggs of Pentatomidae, 16-21.vii.1957, Fahong Yang"; [浙江，杭州，1957.vii.16-21,杨法宏；寄主：蝽象卵]; **Identification:** identifiedBy: Elijah J. Talamas, Huayan Chen; dateIdentified: 2019; **Event:** samplingProtocol: none specified; eventDate: 1957-07-16/21; **Record Level:** modified: 04/29/2020; language: en
- **Type status:** Other material. **Occurrence:** catalogNumber: ZJU 790539-1; recordedBy: Huifen Wu; individualCount: 3; sex: female; lifeStage: adult; associatedOccurrences: Pentatomidae; **Taxon:** scientificName: Trissolcus mitsukurii; order: Hymenoptera; family: Scelionidae; genus: Trissolcus; specificEpithet: mitsukurii; **Location:** country: China; stateProvince: Hunan; county: Changsha; locationRemarks: label transliteration: "Hunan, Changsha, ex. from eggs of Pentatomidae in paddy field, 19, Huifen Wu"; [湖南，长沙，19，吴惠芬；寄主：稻田蝽象]; **Identification:** identifiedBy: Elijah J. Talamas, Huayan Chen; dateIdentified: 2019; **Event:** samplingProtocol: none specified; eventDate: 19; **Record Level:** modified: 04/29/2020; language: en
- **Type status:** Other material. **Occurrence:** catalogNumber: ZJU 790539-2; recordedBy: Huifen Wu; individualCount: 3; sex: 1 female，2 males; lifeStage: adult; associatedOccurrences: Pentatomidae; **Taxon:** scientificName: Trissolcus mitsukurii; order: Hymenoptera; family: Scelionidae; genus: Trissolcus; specificEpithet: mitsukurii; **Location:** country: China; stateProvince: Hunan; county: Changsha; locationRemarks: label transliteration: "Hunan, Changsha, ex. from eggs of Pentatomidae in paddy field, 19, Huifen Wu"; [湖南，长沙，19，吴惠芬；寄主：稻田蝽象]; **Identification:** identifiedBy: Elijah J. Talamas, Huayan Chen; dateIdentified: 2019; **Event:** samplingProtocol: none specified; eventDate: 19; **Record Level:** modified: 04/29/2020; language: en
- **Type status:** Other material. **Occurrence:** catalogNumber: ZJU 790539-3; recordedBy: Huifen Wu; individualCount: 2; sex: female; lifeStage: adult; associatedOccurrences: Pentatomidae; **Taxon:** scientificName: Trissolcus mitsukurii; order: Hymenoptera; family: Scelionidae; genus: Trissolcus; specificEpithet: mitsukurii; **Location:** country: China; stateProvince: Hunan; county: Changsha; locationRemarks: label transliteration: "Hunan, Changsha, ex. from eggs of Pentatomidae in paddy field, 19, Huifen Wu"; [湖南，长沙，19，吴惠芬；寄主：稻田蝽象]; **Identification:** identifiedBy: Elijah J. Talamas, Huayan Chen; dateIdentified: 2019; **Event:** samplingProtocol: none specified; eventDate: 19; **Record Level:** modified: 04/29/2020; language: en
- **Type status:** Other material. **Occurrence:** catalogNumber: ZJU 790539-4; recordedBy: Huifen Wu; individualCount: 3; sex: female; lifeStage: adult; associatedOccurrences: Pentatomidae; **Taxon:** scientificName: Trissolcus mitsukurii; order: Hymenoptera; family: Scelionidae; genus: Trissolcus; specificEpithet: mitsukurii; **Location:** country: China; stateProvince: Hunan; county: Changsha; locationRemarks: label transliteration: "Hunan, Changsha, ex. from eggs of Pentatomidae in paddy field, 19, Huifen Wu"; [湖南，长沙，19，吴惠芬；寄主：稻田蝽象]; **Identification:** identifiedBy: Elijah J. Talamas, Huayan Chen; dateIdentified: 2019; **Event:** samplingProtocol: none specified; eventDate: 19; **Record Level:** modified: 04/29/2020; language: en
- **Type status:** Other material. **Occurrence:** catalogNumber: ZJU 790539-5; recordedBy: Huifen Wu; individualCount: 3; sex: 1 female，2 males; lifeStage: adult; associatedOccurrences: Pentatomidae; **Taxon:** scientificName: Trissolcus mitsukurii; order: Hymenoptera; family: Scelionidae; genus: Trissolcus; specificEpithet: mitsukurii; **Location:** country: China; stateProvince: Hunan; county: Changsha; locationRemarks: label transliteration: "Hunan, Changsha, ex. from eggs of Pentatomidae in paddy field, 19, Huifen Wu"; [湖南，长沙，19，吴惠芬；寄主：稻田蝽象]; **Identification:** identifiedBy: Elijah J. Talamas, Huayan Chen; dateIdentified: 2019; **Event:** samplingProtocol: none specified; eventDate: 19; **Record Level:** modified: 04/29/2020; language: en
- **Type status:** Other material. **Occurrence:** catalogNumber: ZJU 790539-6; recordedBy: Huifen Wu; individualCount: 4; sex: 3 females，1 male; lifeStage: adult; associatedOccurrences: Pentatomidae; **Taxon:** scientificName: Trissolcus mitsukurii; order: Hymenoptera; family: Scelionidae; genus: Trissolcus; specificEpithet: mitsukurii; **Location:** country: China; stateProvince: Hunan; county: Changsha; locationRemarks: label transliteration: "Hunan, Changsha, ex. from eggs of Pentatomidae in paddy field, 19, Huifen Wu"; [湖南，长沙，19，吴惠芬；寄主：稻田蝽象]; **Identification:** identifiedBy: Elijah J. Talamas, Huayan Chen; dateIdentified: 2019; **Event:** samplingProtocol: none specified; eventDate: 19; **Record Level:** modified: 04/29/2020; language: en
- **Type status:** Other material. **Occurrence:** catalogNumber: ZJU 790539-7; recordedBy: Huifen Wu; individualCount: 2; sex: female; lifeStage: adult; associatedOccurrences: Pentatomidae; **Taxon:** scientificName: Trissolcus mitsukurii; order: Hymenoptera; family: Scelionidae; genus: Trissolcus; specificEpithet: mitsukurii; **Location:** country: China; stateProvince: Hunan; county: Changsha; locationRemarks: label transliteration: "Hunan, Changsha, ex. from eggs of Pentatomidae in paddy field, 19, Huifen Wu"; [湖南，长沙，19，吴惠芬；寄主：稻田蝽象]; **Identification:** identifiedBy: Elijah J. Talamas, Huayan Chen; dateIdentified: 2019; **Event:** samplingProtocol: none specified; eventDate: 19; **Record Level:** modified: 04/29/2020; language: en
- **Type status:** Other material. **Occurrence:** catalogNumber: ZJU 790539-8; recordedBy: Huifen Wu; individualCount: 3; sex: 2 females，1 male; lifeStage: adult; associatedOccurrences: Pentatomidae; **Taxon:** scientificName: Trissolcus japonicus; order: Hymenoptera; family: Scelionidae; genus: Trissolcus; specificEpithet: mitsukurii; **Location:** country: China; stateProvince: Hunan; county: Changsha; locationRemarks: label transliteration: "Hunan, Changsha, ex. from eggs of Pentatomidae in paddy field, 19, Huifen Wu"; [湖南，长沙，19，吴惠芬；寄主：稻田蝽象]; **Identification:** identifiedBy: Elijah J. Talamas, Huayan Chen; dateIdentified: 2019; **Event:** samplingProtocol: none specified; eventDate: 19; **Record Level:** modified: 04/29/2020; language: en

#### Distribution

China (Guizhou, Zhejiang, Jiangxi, Hunan); Italy, Japan, South Korea, Thailand and the United States (quarantine) ([Bibr B5756497][Bibr B5756420])

#### Biology

**Host associations.** This species was reared from the eggs of some unidentified species of Pentatomidae in China.

#### Images

**Figure 6.**
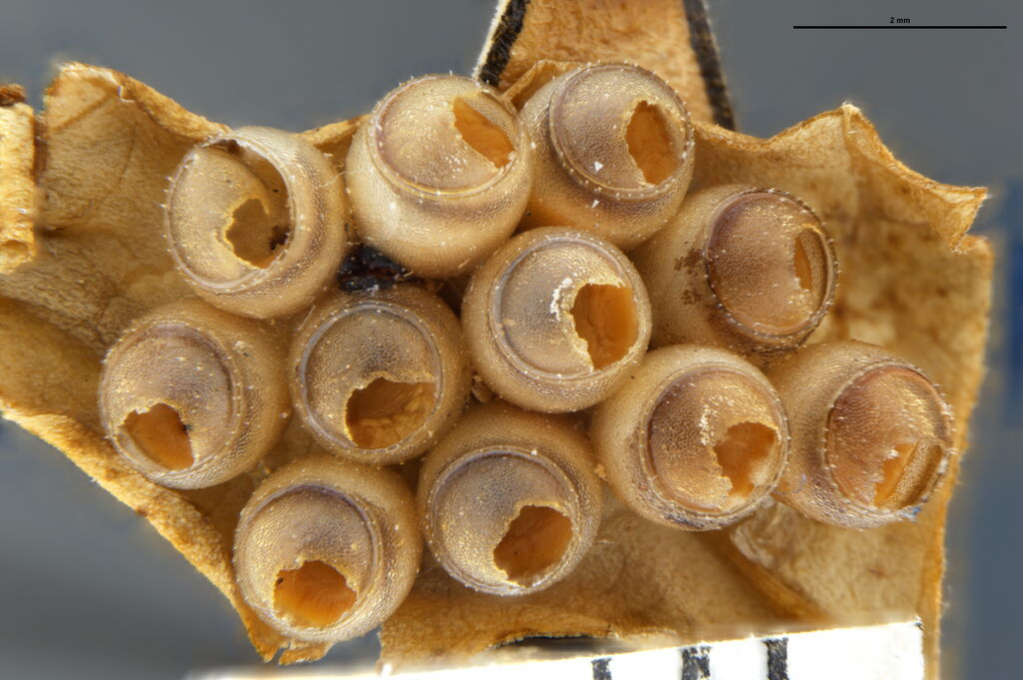
Host egg mass of*Trissolcus mitsukurii* (ZJU 816020-1)

*Trissolcus mitsukurii* Ashmead 1904 (Figs 5, 6).

### Trissolcus semistriatus

(Nees von Esenbeck) 1834

5082FC75-13D5-56B9-AE2E-9AA522D927B4

#### Materials

- **Type status:** Other material. **Occurrence:** catalogNumber: ZJU 5757.9-1; individualCount: 1; sex: female; lifeStage: adult; associatedOccurrences: Eurydema sp.; **Taxon:** scientificName: Trissolcus semistriatus; order: Hymenoptera; family: Scelionidae; genus: Trissolcus; specificEpithet: semistriatus; **Location:** country: China; stateProvince: Xinjiang; locationRemarks: label transliteration: "Xinjiang, ex. from eggs of Eurydema sp., 1956-1957, Bayi Agricultural College (Xinjiang Agricultural University)"; [新疆，1956-1957，八一农院昆虫组，寄主：红花菜蝽象卵 Eurydema sp.]; **Identification:** identifiedBy: Elijah J. Talamas, Huayan Chen; dateIdentified: 2019; **Event:** samplingProtocol: none specified; eventDate: 1956-1957; **Record Level:** modified: 04/29/2020; language: en
- **Type status:** Other material. **Occurrence:** catalogNumber: ZJU 5757.9-2; individualCount: 1; sex: female; lifeStage: adult; associatedOccurrences: Eurydema sp.; **Taxon:** scientificName: Trissolcus semistriatus; order: Hymenoptera; family: Scelionidae; genus: Trissolcus; specificEpithet: semistriatus; **Location:** country: China; stateProvince: Xinjiang; locationRemarks: label transliteration: "Xinjiang, ex. from eggs of Eurydema sp., 1956-1957, Bayi Agricultural College (Xinjiang Agricultural University)"; [新疆，1956-1957，八一农院昆虫组，寄主：红花菜蝽象卵 Eurydema sp.]; **Identification:** identifiedBy: Elijah J. Talamas, Huayan Chen; dateIdentified: 2019; **Event:** samplingProtocol: none specified; eventDate: 1956-1957; **Record Level:** modified: 04/29/2020; language: en
- **Type status:** Other material. **Occurrence:** catalogNumber: ZJU 5757.8; individualCount: 1; sex: female; lifeStage: adult; associatedOccurrences: Eurydema sp.; **Taxon:** scientificName: Trissolcus semistriatus; order: Hymenoptera; family: Scelionidae; genus: Trissolcus; specificEpithet: semistriatus; **Location:** country: China; stateProvince: Xinjiang; locationRemarks: label transliteration: "Xinjiang, ex. from eggs of Eurydema sp., 1956-1957, Bayi Agricultural College (Xinjiang Agricultural University)"; [新疆，1956-1957，八一农院昆虫组，寄主：红花菜蝽象卵 Eurydema sp.]; **Identification:** identifiedBy: Elijah J. Talamas, Huayan Chen; dateIdentified: 2019; **Event:** samplingProtocol: none specified; eventDate: 1956-1957; **Record Level:** modified: 04/29/2020; language: en
- **Type status:** Other material. **Occurrence:** catalogNumber: ZJU 76086-1; recordedBy: Institute of Agricultural Sciences of Xianyang; individualCount: 5; sex: female; lifeStage: adult; associatedOccurrences: Pentatomidae; **Taxon:** scientificName: Trissolcus semistriatus; order: Hymenoptera; family: Scelionidae; genus: Trissolcus; specificEpithet: semistriatus; **Location:** country: China; stateProvince: Shaanxi; county: Xianyang; locationRemarks: label transliteration: “Shaanxi, Xianyang, ex. from eggs of Pentatomidae, viii.1975, Institute of Agricultural Sciences of Xianyang"; [陕西，咸阳，1975.viii，咸阳地区农科所；寄主：蝽象]; **Identification:** identifiedBy: Elijah J. Talamas, Huayan Chen; dateIdentified: 2019; **Event:** samplingProtocol: none specified; eventDate: 1975-08; **Record Level:** modified: 04/29/2020; language: en
- **Type status:** Other material. **Occurrence:** catalogNumber: ZJU 76086-2; recordedBy: Institute of Agricultural Sciences of Xianyang; individualCount: 10; sex: female，egg mass; lifeStage: adult; associatedOccurrences: Pentatomidae; **Taxon:** scientificName: Trissolcus semistriatus; order: Hymenoptera; family: Scelionidae; genus: Trissolcus; specificEpithet: semistriatus; **Location:** country: China; stateProvince: Shaanxi; county: Xianyang; locationRemarks: label transliteration: “Shaanxi, Xianyang, ex. from eggs of Pentatomidae, viii.1975, Institute of Agricultural Sciences of Xianyang"; [陕西，咸阳，1975.viii，咸阳地区农科所；寄主：蝽象]; **Identification:** identifiedBy: Elijah J. Talamas, Huayan Chen; dateIdentified: 2019; **Event:** samplingProtocol: none specified; eventDate: 1975-08; **Record Level:** modified: 04/29/2020; language: en
- **Type status:** Other material. **Occurrence:** catalogNumber: ZJU 76086-3; recordedBy: Institute of Agricultural Sciences of Xianyang; individualCount: 5; sex: 4 females，1 male; lifeStage: adult; associatedOccurrences: Pentatomidae; **Taxon:** scientificName: Trissolcus semistriatus; order: Hymenoptera; family: Scelionidae; genus: Trissolcus; specificEpithet: semistriatus; **Location:** country: China; stateProvince: Shaanxi; county: Xianyang; locationRemarks: label transliteration: “Shaanxi, Xianyang, ex. from eggs of Pentatomidae, viii.1975, Institute of Agricultural Sciences of Xianyang"; [陕西，咸阳，1975.viii，咸阳地区农科所；寄主：蝽象]; **Identification:** identifiedBy: Elijah J. Talamas, Huayan Chen; dateIdentified: 2019; **Event:** samplingProtocol: none specified; eventDate: 1975-08; **Record Level:** modified: 04/29/2020; language: en
- **Type status:** Other material. **Occurrence:** catalogNumber: ZJU 76086-4; recordedBy: Institute of Agricultural Sciences of Xianyang; individualCount: 5; sex: female; lifeStage: adult; associatedOccurrences: Pentatomidae; **Taxon:** scientificName: Trissolcus semistriatus; **Location:** country: China; stateProvince: Shaanxi; county: Xianyang; locationRemarks: label transliteration: “Shaanxi, Xianyang, ex. from eggs of Pentatomidae, viii.1975, Institute of Agricultural Sciences of Xianyang"; [陕西，咸阳，1975.viii，咸阳地区农科所；寄主：蝽象]; **Identification:** identifiedBy: Elijah J. Talamas, Huayan Chen; dateIdentified: 2019; **Event:** samplingProtocol: none specified; eventDate: 1975-08; **Record Level:** modified: 04/29/2020; language: en
- **Type status:** Other material. **Occurrence:** catalogNumber: ZJU 76086-5; recordedBy: Institute of Agricultural Sciences of Xianyang; individualCount: 2; sex: 1 female，1 male; lifeStage: adult; associatedOccurrences: Pentatomidae; **Taxon:** scientificName: Trissolcus semistriatus; **Location:** country: China; stateProvince: Shaanxi; county: Xianyang; locationRemarks: label transliteration: “Shaanxi, Xianyang, ex. from eggs of Pentatomidae, viii.1975, Institute of Agricultural Sciences of Xianyang"; [陕西，咸阳，1975.viii，咸阳地区农科所；寄主：蝽象]; **Identification:** identifiedBy: Elijah J. Talamas, Huayan Chen; dateIdentified: 2019; **Event:** samplingProtocol: none specified; eventDate: 1975-08; **Record Level:** modified: 04/29/2020; language: en

#### Distribution

China (Xinjiang, Shaanxi); Italy, Morocco, Portugal, Sweden and Switzerland (Tortorici et al. 2019).

#### Biology

**Host associations.** This species was reared from the eggs of *Eurydema* sp. (Pentatomidae) and an unidentified species of Pentatomidae in China.

#### Images

*Trissolcus semistriatus* Nees von Esenbeck 1834 (Fig. 7).

### Trissolcus yamagishii

Ryu 1984

8AF921C4-ECEA-5F48-A6BA-0D9B77203A0F

#### Materials

- **Type status:** Other material. **Occurrence:** catalogNumber: ZJU 5414.8-1; recordedBy: Songyun Mai; individualCount: 1; sex: female; lifeStage: adult; associatedOccurrences: Niphe elongata; **Taxon:** scientificName: Trissolcus yamagishii; order: Hymenoptera; family: Scelionidae; genus: Trissolcus; specificEpithet: yamagishii; **Location:** country: China; stateProvince: Hunan; county: Changsha; locality: Wangcheng; locationRemarks: label transliteration: "Hunan, Changsha, Wangcheng District, ex. from eggs of Niphe elongata , 22.vii.1954, Songyun Mai"; [湖南，长沙望城，1954.vii.22,麦松云；寄主：褐蝽象卵Niphe elongata]; **Identification:** identifiedBy: Elijah J. Talamas, Huayan Chen; dateIdentified: 2019; **Event:** samplingProtocol: none specified; eventDate: 07/22/1954; **Record Level:** modified: 04/29/2020; language: en
- **Type status:** Other material. **Occurrence:** catalogNumber: ZJU 5414.8-2; recordedBy: Songyun Mai; individualCount: 1; sex: female; lifeStage: adult; associatedOccurrences: Niphe elongata; **Taxon:** scientificName: Trissolcus yamagishii; order: Hymenoptera; family: Scelionidae; genus: Trissolcus; specificEpithet: yamagishii; **Location:** country: China; stateProvince: Hunan; county: Changsha; locality: Wangcheng; locationRemarks: label transliteration: "Hunan, Changsha, Wangcheng District, ex. from eggs of Niphe elongata , 22.vii.1954, Songyun Mai"; [湖南，长沙望城，1954.vii.22,麦松云；寄主：褐蝽象卵Niphe elongata]; **Identification:** identifiedBy: Elijah J. Talamas, Huayan Chen; dateIdentified: 2019; **Event:** samplingProtocol: none specified; eventDate: 07/22/1954; **Record Level:** modified: 04/29/2020; language: en
- **Type status:** Other material. **Occurrence:** catalogNumber: ZJU 5414.8-3; recordedBy: Songyun Mai; individualCount: 1; sex: female; lifeStage: adult; associatedOccurrences: Niphe elongata; **Taxon:** scientificName: Trissolcus yamagishii; order: Hymenoptera; family: Scelionidae; genus: Trissolcus; specificEpithet: yamagishii; **Location:** country: China; stateProvince: Hunan; county: Changsha; locality: Wangcheng; locationRemarks: label transliteration: "Hunan, Changsha, Wangcheng District, ex. from eggs of Niphe elongata , 22.vii.1954, Songyun Mai"; [湖南，长沙望城，1954.vii.22,麦松云；寄主：褐蝽象卵Niphe elongata]; **Identification:** identifiedBy: Elijah J. Talamas, Huayan Chen; dateIdentified: 2019; **Event:** samplingProtocol: none specified; eventDate: 07/22/1954; **Record Level:** modified: 04/29/2020; language: en
- **Type status:** Other material. **Occurrence:** catalogNumber: ZJU 5414.8-4; recordedBy: Songyun Mai; individualCount: 1; sex: female; lifeStage: adult; associatedOccurrences: Niphe elongata; **Taxon:** scientificName: Trissolcus yamagishii; order: Hymenoptera; family: Scelionidae; genus: Trissolcus; specificEpithet: yamagishii; **Location:** country: China; stateProvince: Hunan; county: Changsha; locality: Wangcheng; locationRemarks: label transliteration: "Hunan, Changsha, Wangcheng District, ex. from eggs of Niphe elongata , 22.vii.1954, Songyun Mai"; [湖南，长沙望城，1954.vii.22,麦松云；寄主：褐蝽象卵Niphe elongata]; **Identification:** identifiedBy: Elijah J. Talamas, Huayan Chen; dateIdentified: 2019; **Event:** samplingProtocol: none specified; eventDate: 07/22/1954; **Record Level:** modified: 04/29/2020; language: en

#### Distribution

China (Hunan); India, Laos, South Korea, United Arab Emirates and Vietnam (Talamas et al. 2017).

#### Biology

**Host associations.** This species was reared from the eggs of *Niphe elongata* (Dallas) (Pentatomidae) in China.

#### Notes

This species is newly recorded from China.

#### Images

Trissolcus yamagishii Ryu and Hirashima 1984 (Fig. 8).

## Analysis

**Summary of host associations**

A total of 123 specimens were examined and seven species are recognized. The hosts recorded on specimen label data are summarized below:

Pentatomidae

*Erthesina fullo* (Thunberg): *T. japonicus*

*Eurydema* sp.: *T. semistriatus*

*Hippotiscus dorsalis* Stål: *T. cultratus*

*Niphe elongata* (Dallas): *T. elasmuchae*, *T. yamagishii*

*Rhaphigaster nebulosa* (Poda): *T. japonicus*

Scutelleridae

*Poecilocoris latus* Dallas: *T. latisulcus*

Urostylididae

*Urochela luteovaria* Distant: *T. cultratus*

## Discussion

Specimens housed in museum and university collections are an immense and often irreplaceable source of biological information. This study produced immediately-applicable data that was gleaned from a historic collection simply by identifying specimens and recording label data. It is difficult and, sometimes, impossible to identify host eggs to species, based on morphology alone and several *Trissolcus* species in our study were reared from eggs identified only as Pentatomidae. New methods in molecular diagnostics that can identify trophic interactions from parasitized eggs have been used to overcome this challenge and identify associations between scelionid parasitoids and stink bugs (Gariepy et al. 2018, Lomeli-Flores et al. 2019). Implementation of this approach on specimens in insect collections can further expand the data that can be harvested from these institutional resources.

## Supplementary Material

XML Treatment for Trissolcus cultratus

XML Treatment for Trissolcus elasmuchae

XML Treatment for Trissolcus japonicus

XML Treatment for Trissolcus latisulcus

XML Treatment for Trissolcus mitsukurii

XML Treatment for Trissolcus semistriatus

XML Treatment for Trissolcus yamagishii

## Figures and Tables

**Figure 1a.**
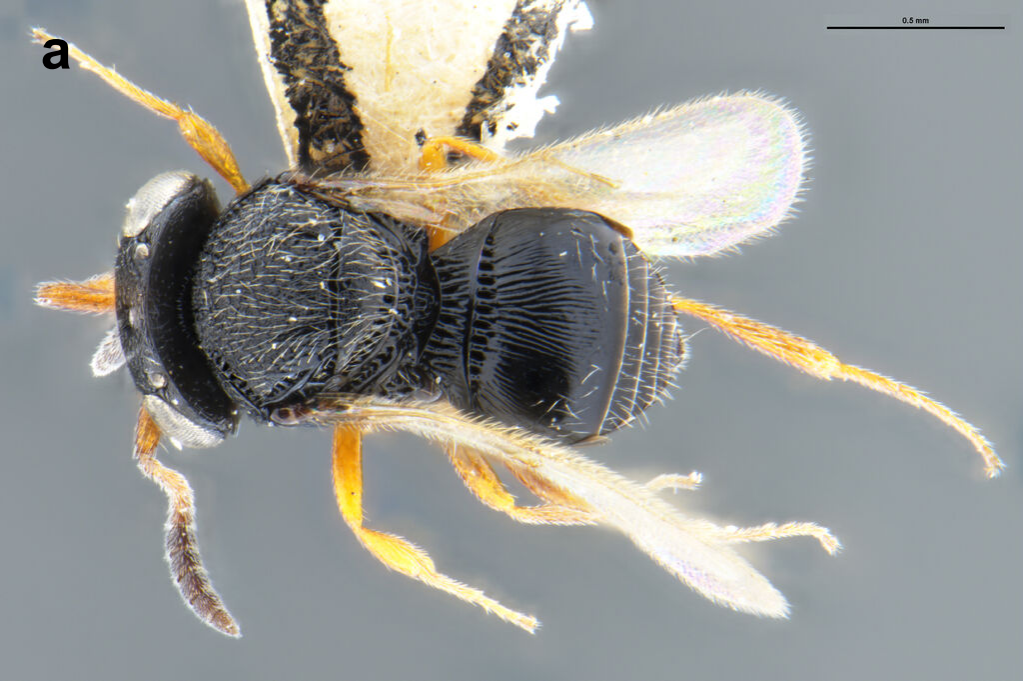
dorsal habitus

**Figure 1b.**
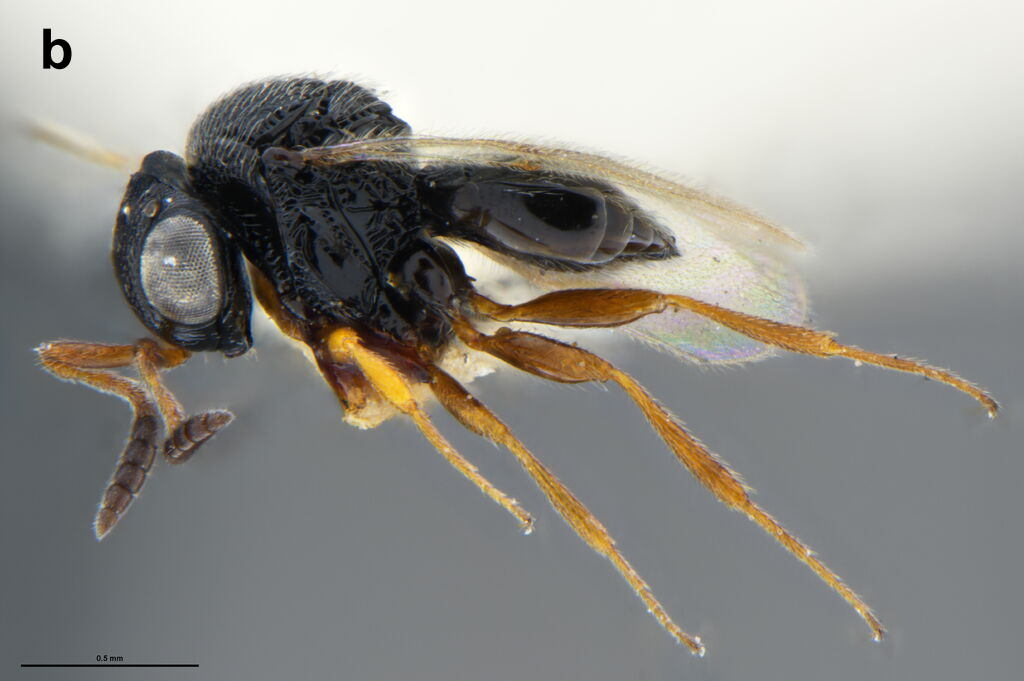
lateral habitus

**Figure 1c.**
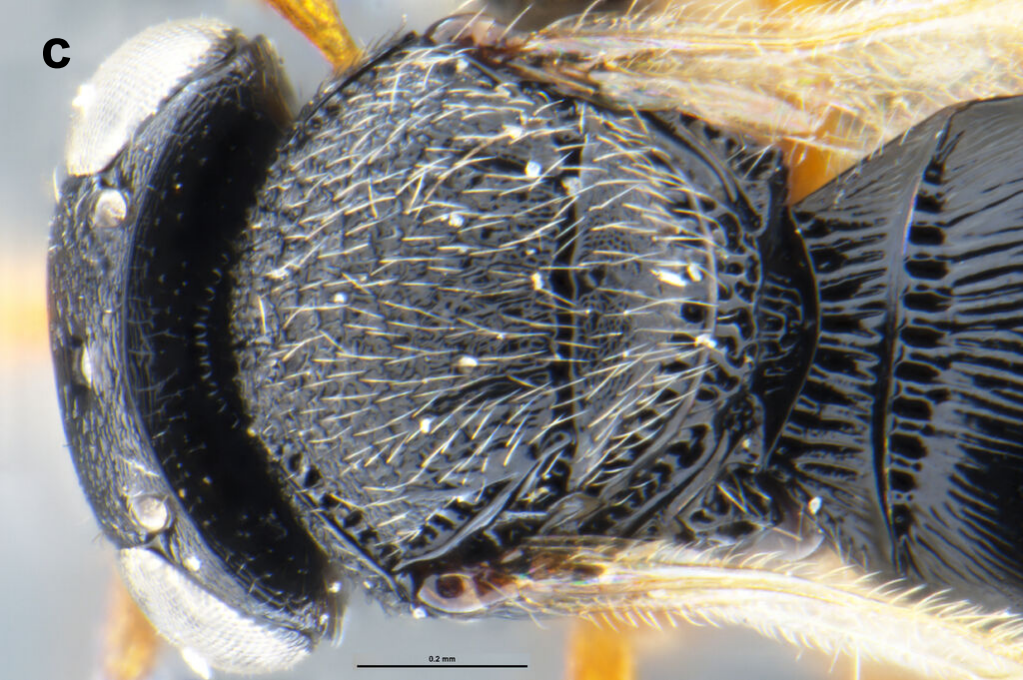
head and mesosoma, dorsal view

**Figure 1d.**
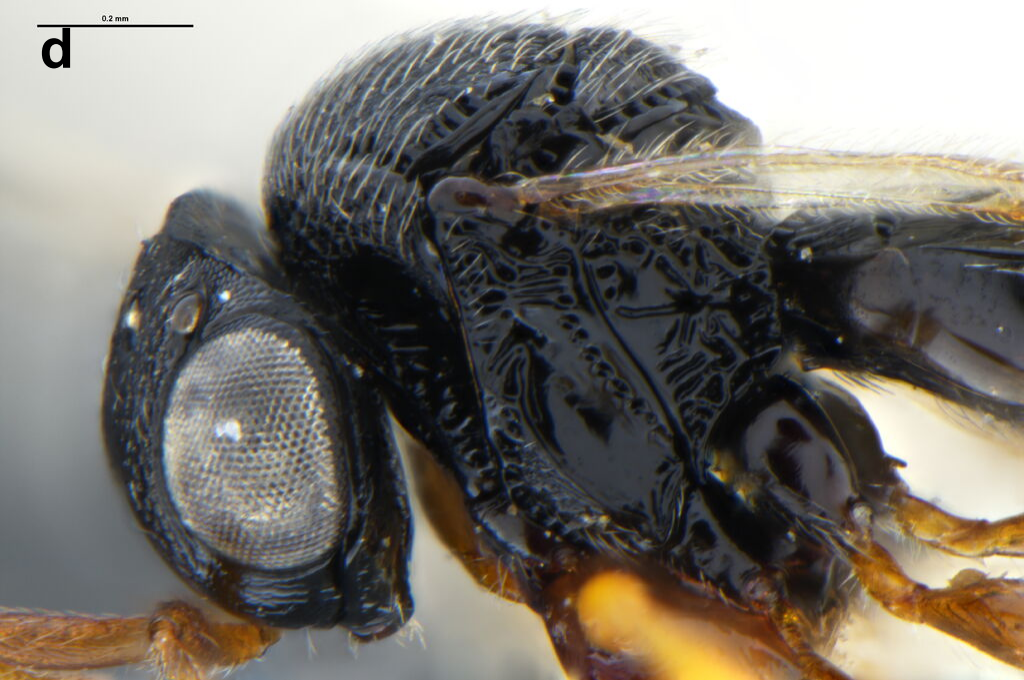
head and mesosoma, lateral view

**Figure 1e.**
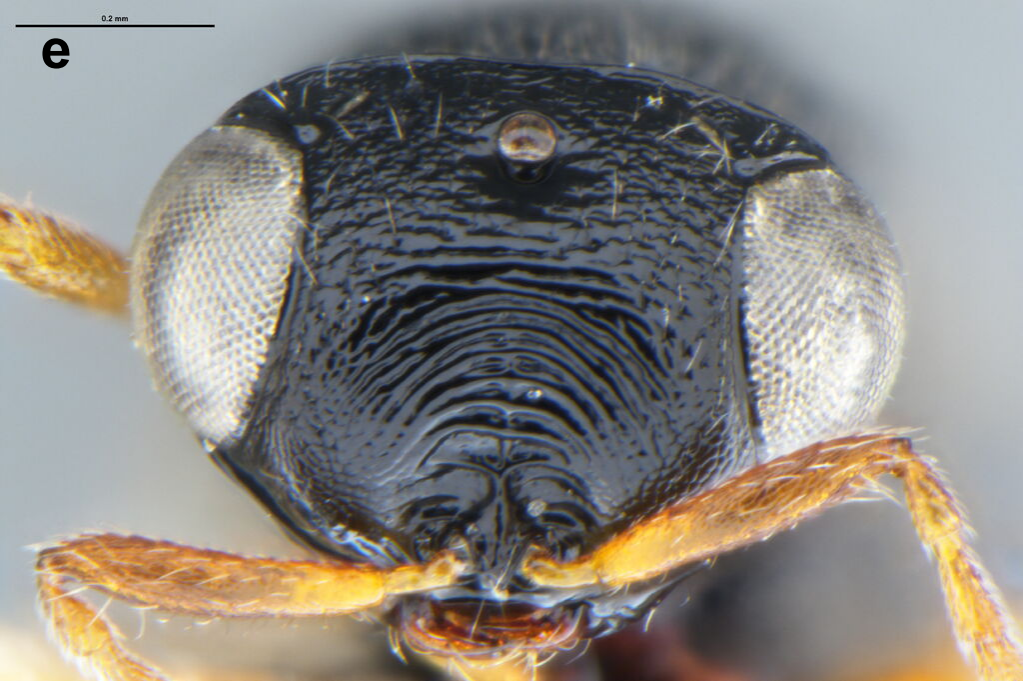
head, anterior view

**Figure 1f.**
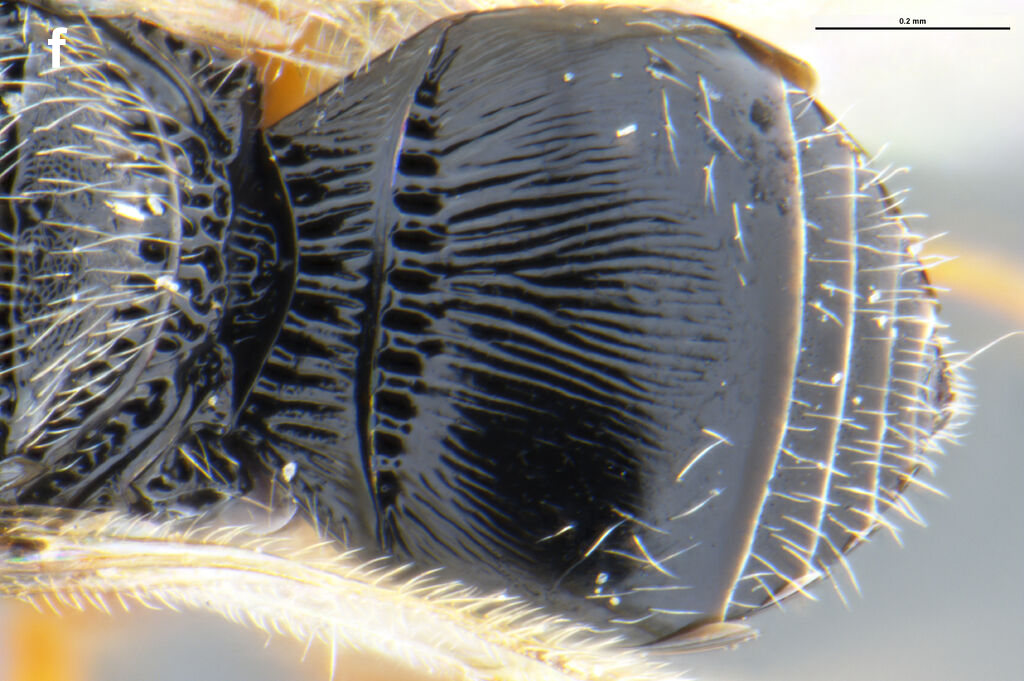
metasoma, dorsal view

**Figure 2a.**
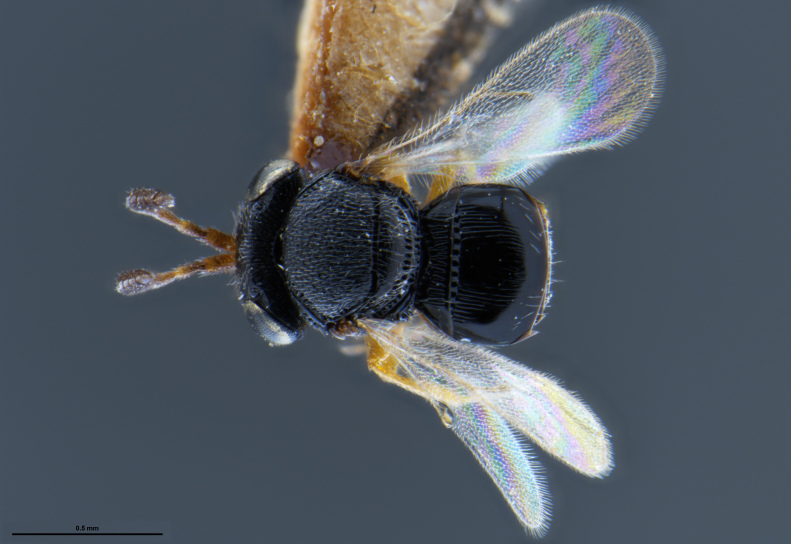
dorsal habitus

**Figure 2b.**
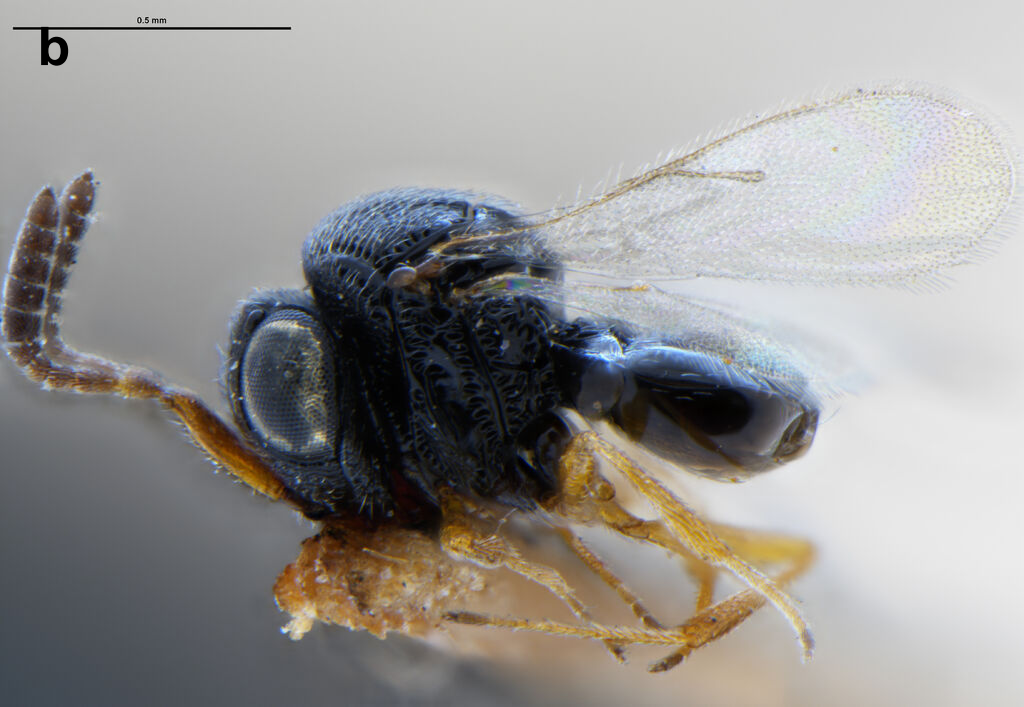
lateral habitus

**Figure 2c.**
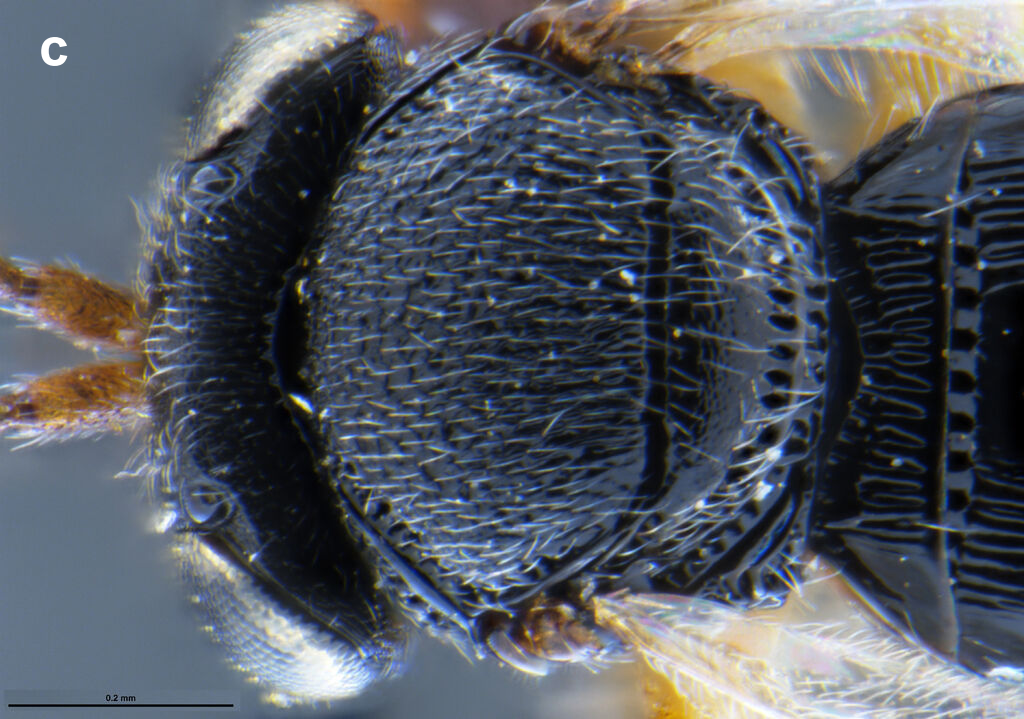
head and mesosoma, dorsal view

**Figure 2d.**
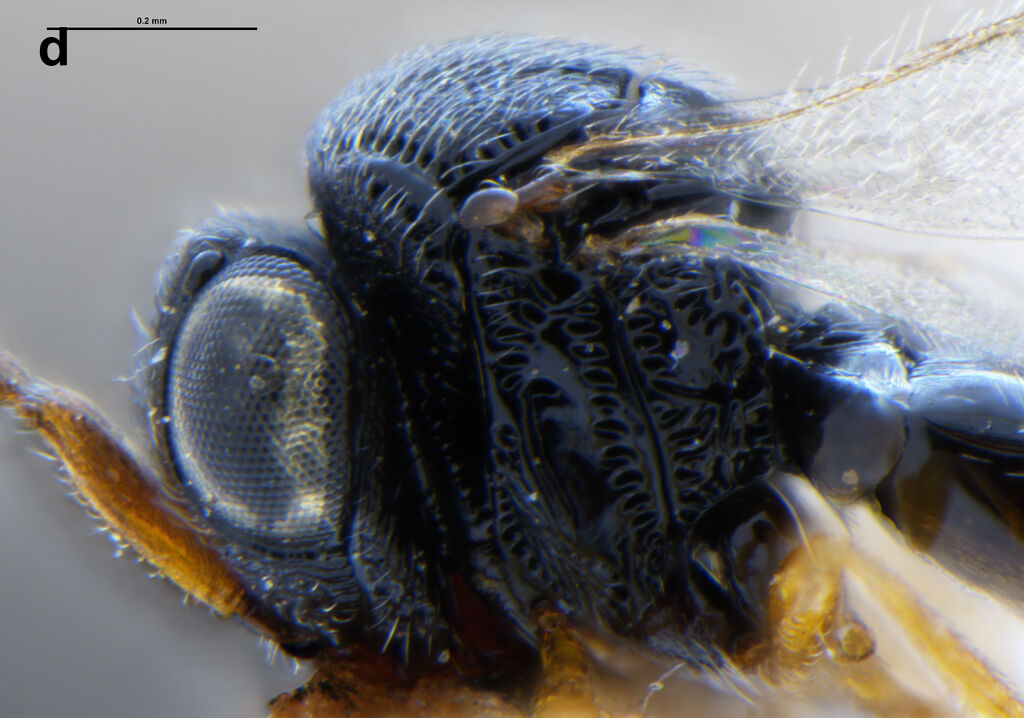
head and mesosoma, lateral view

**Figure 2e.**
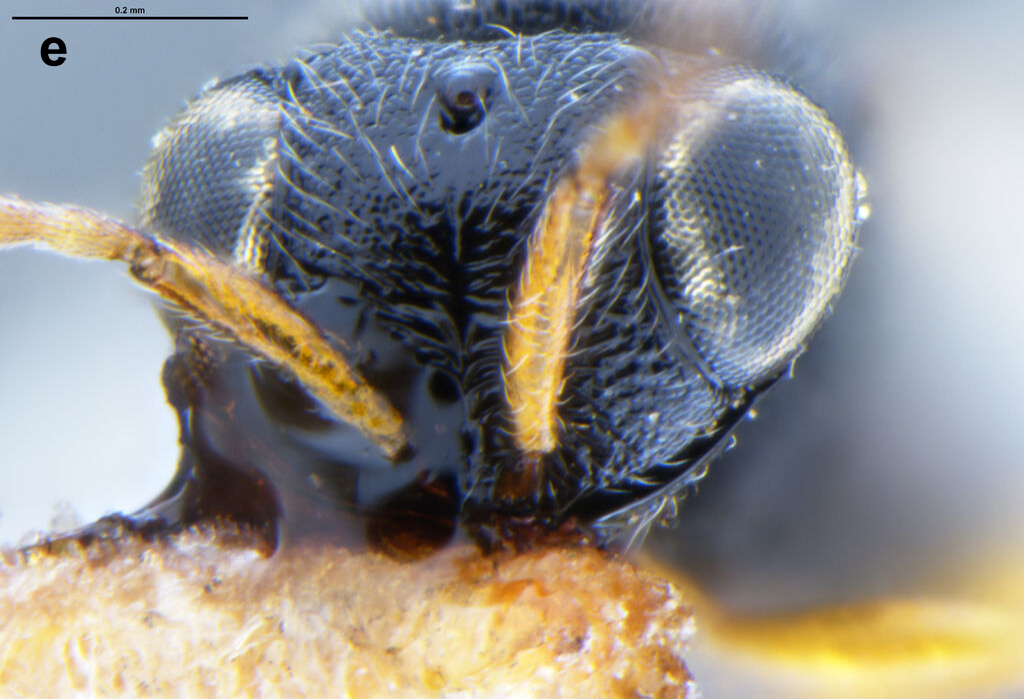
head, anterior view

**Figure 2f.**
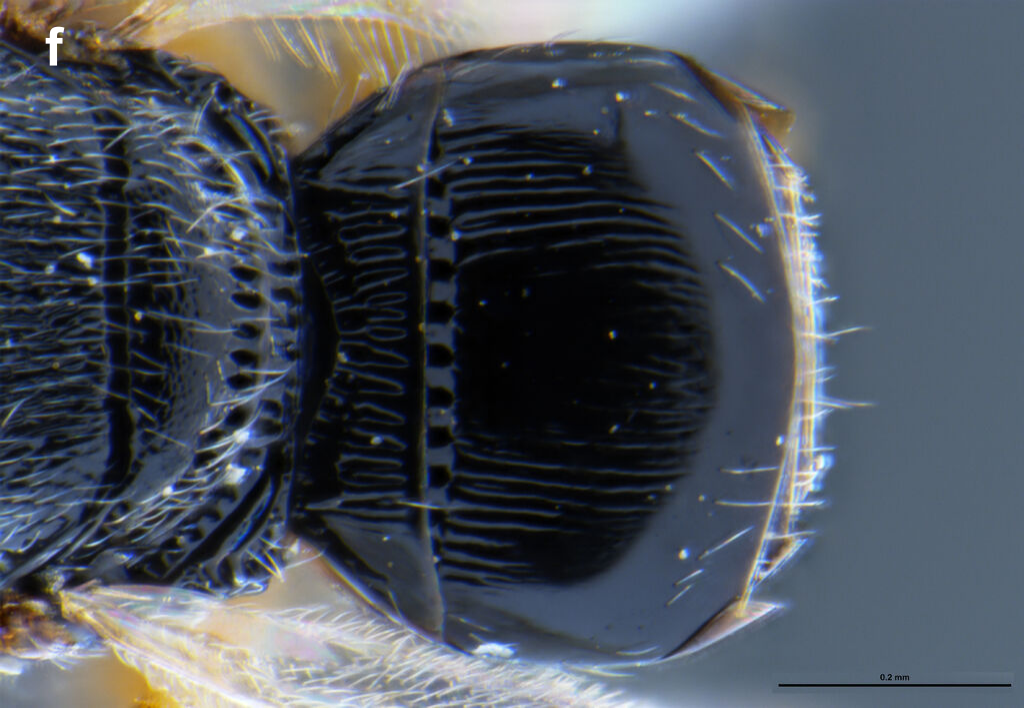
metasoma, dorsal view

**Figure 3a.**
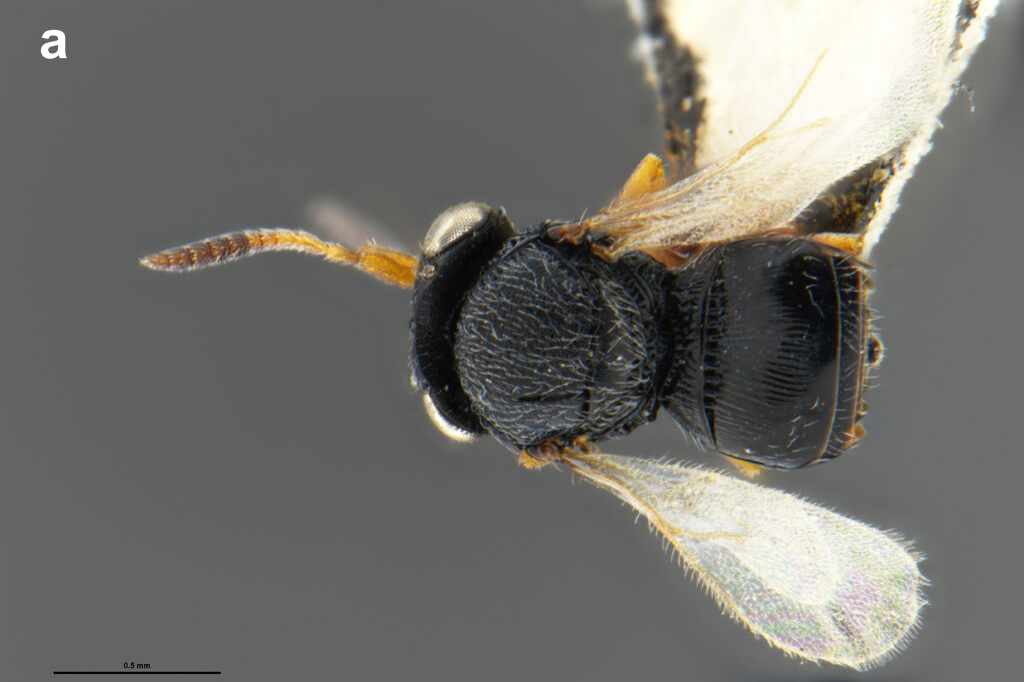
dorsal habitus

**Figure 3b.**
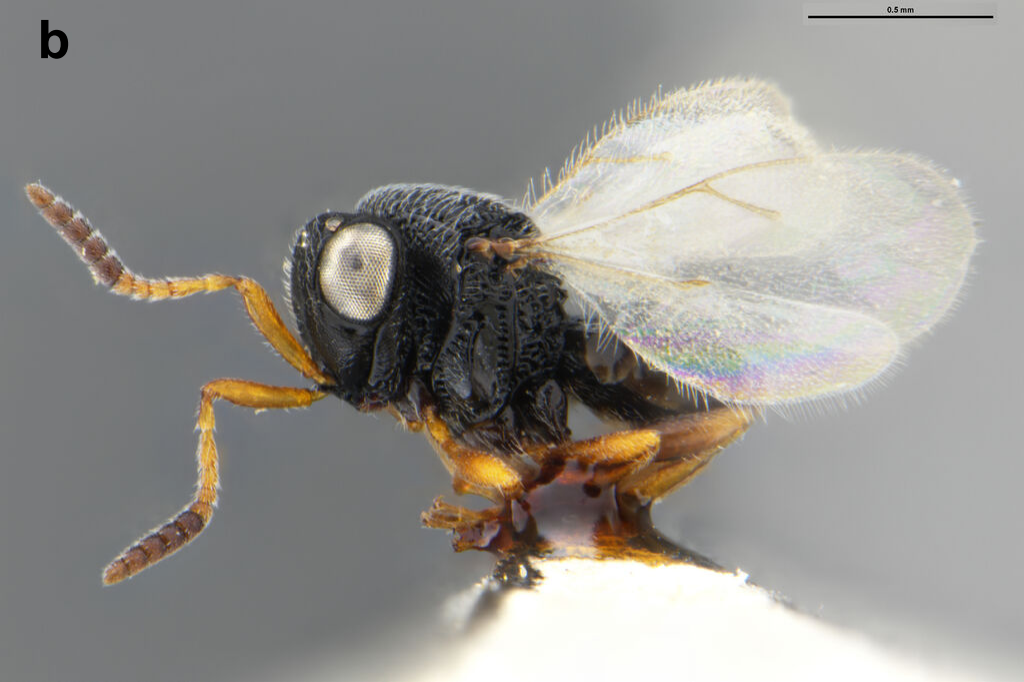
lateral habitus

**Figure 3c.**
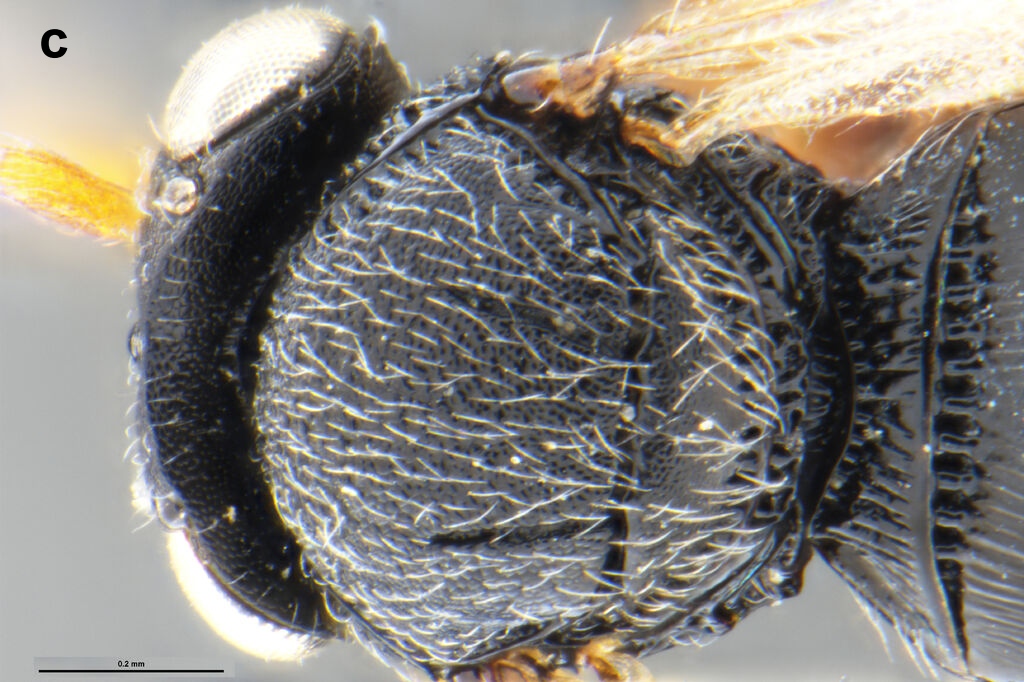
head and mesosoma, dorsal view

**Figure 3d.**
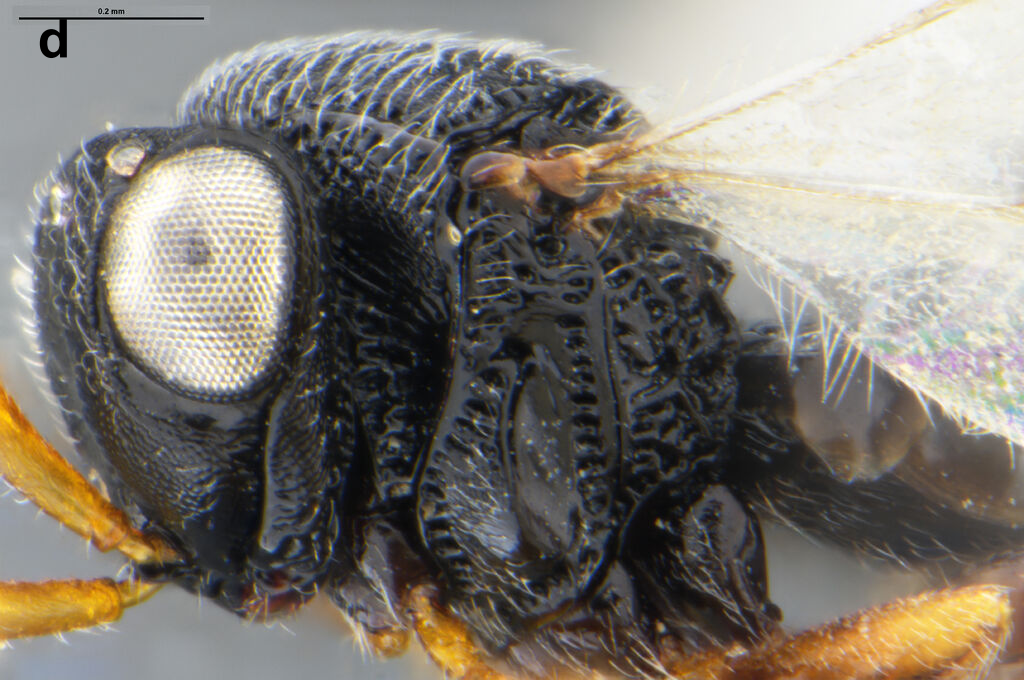
head and mesosoma, lateral view

**Figure 3e.**
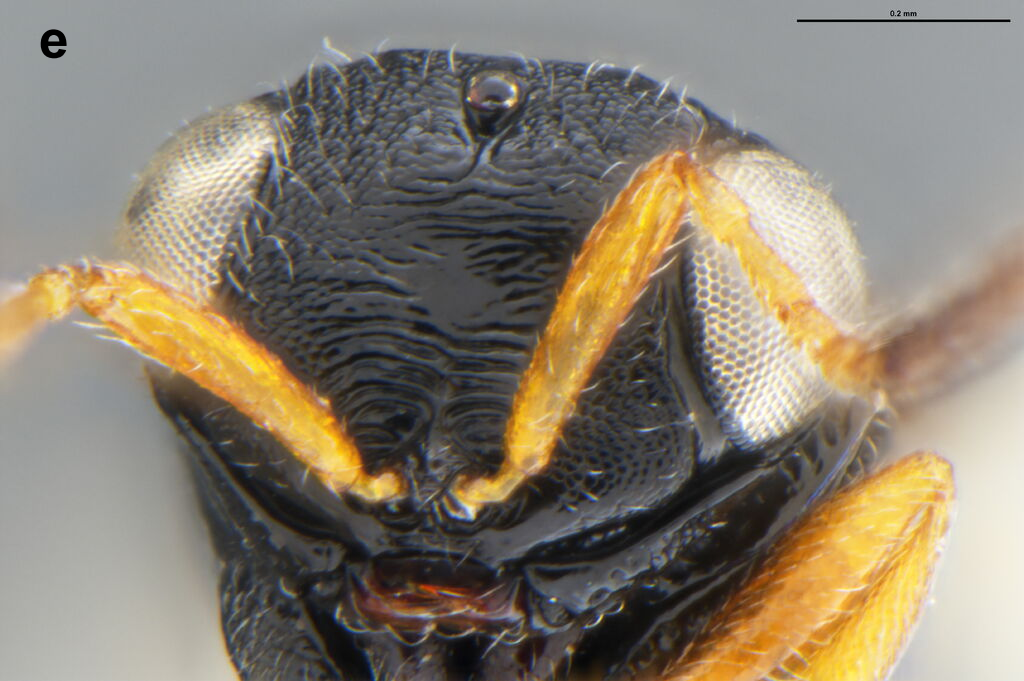
head, anterior view

**Figure 3f.**
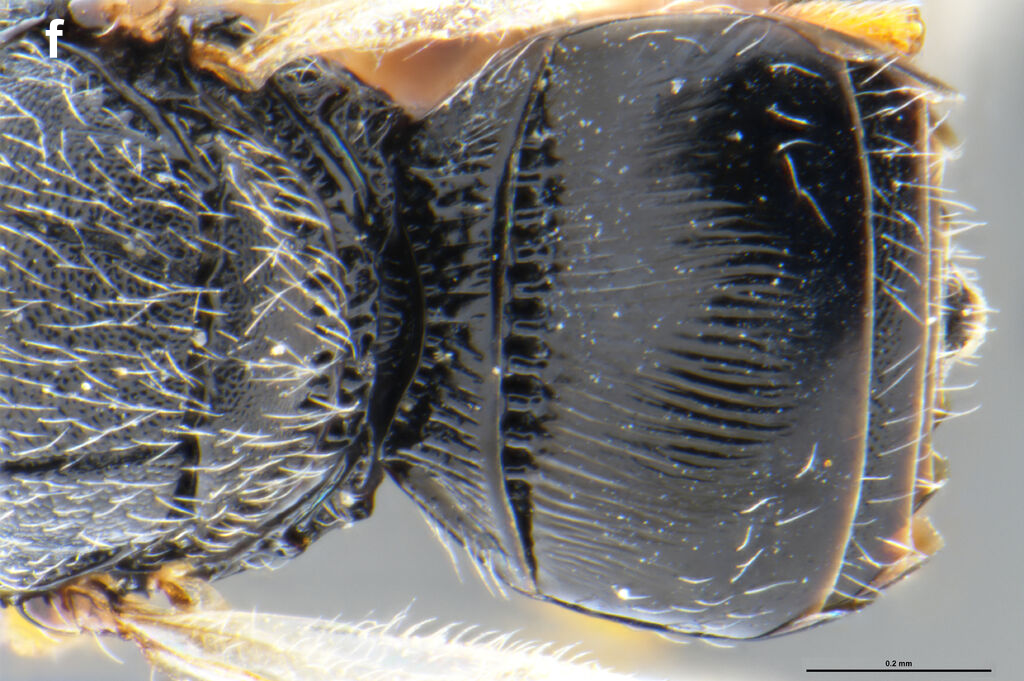
metasoma, dorsal view

**Figure 4a.**
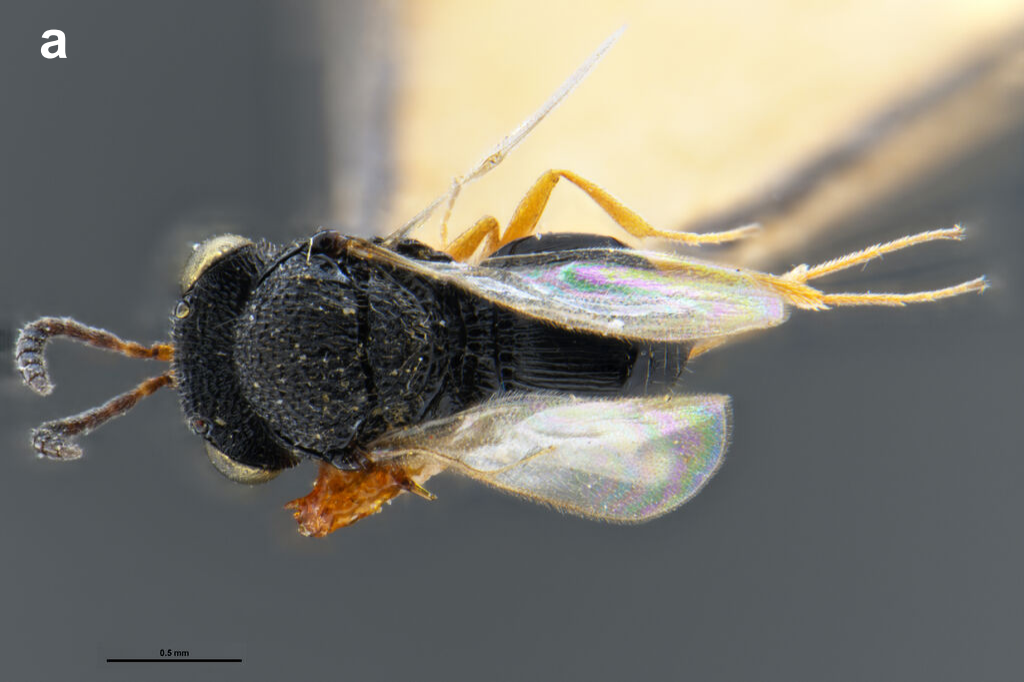
dorsal habitus

**Figure 4b.**
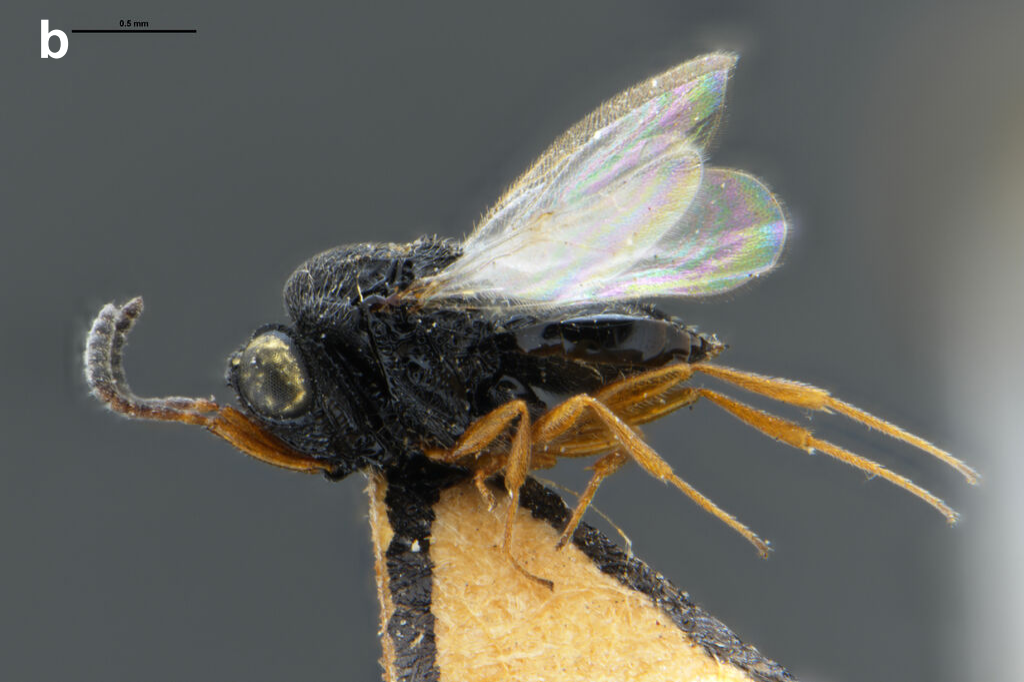
lateral habitus

**Figure 4c.**
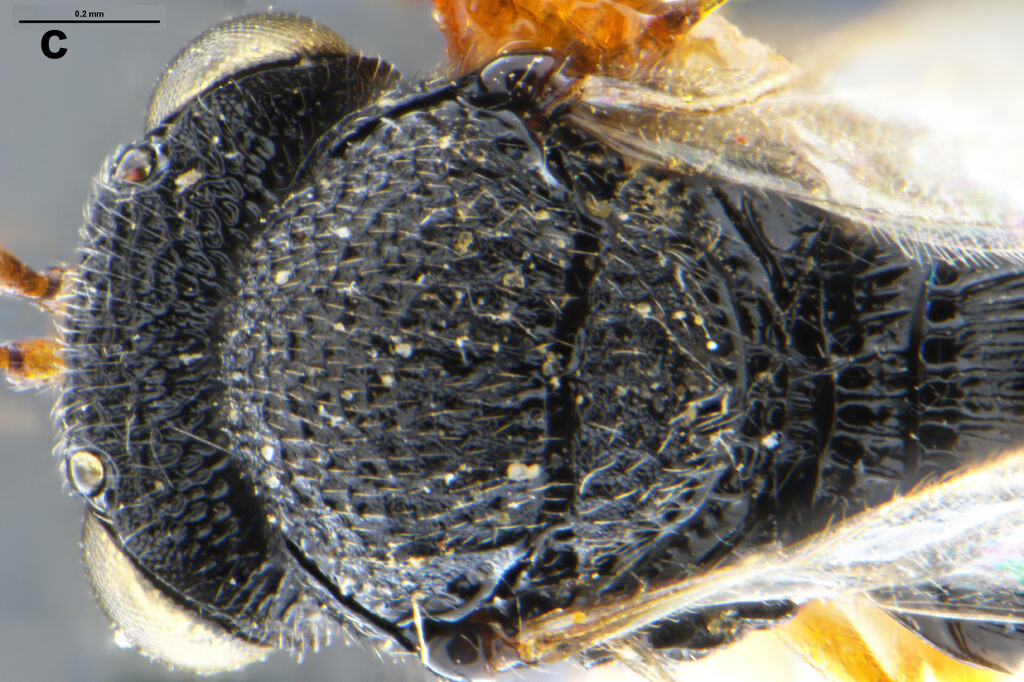
head and mesosoma, dorsal view

**Figure 4d.**
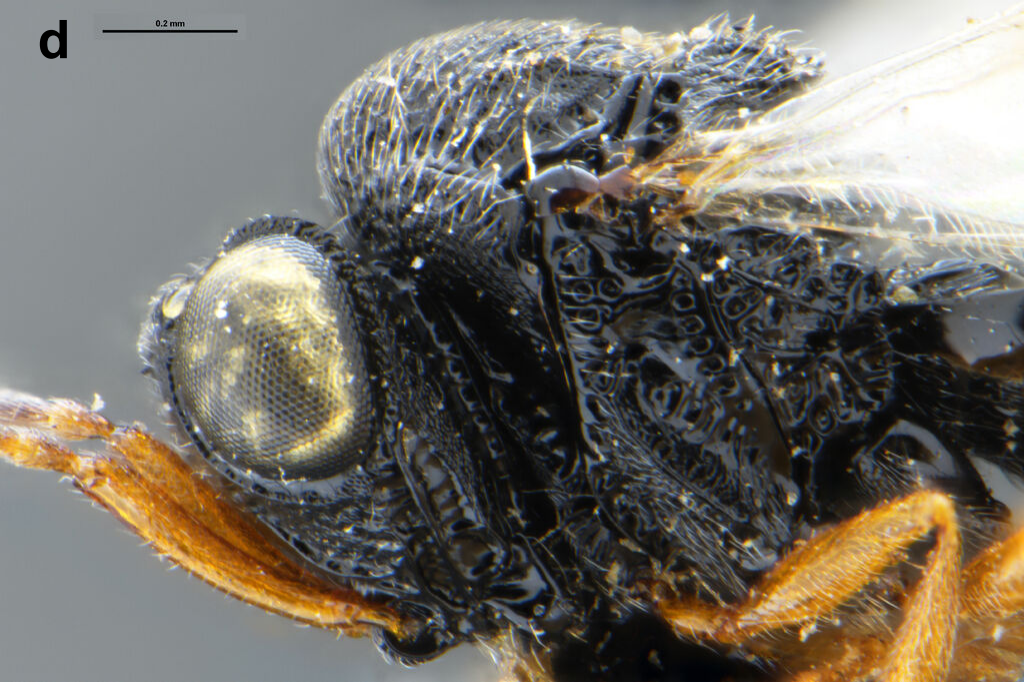
head and mesosoma, lateral view

**Figure 4e.**
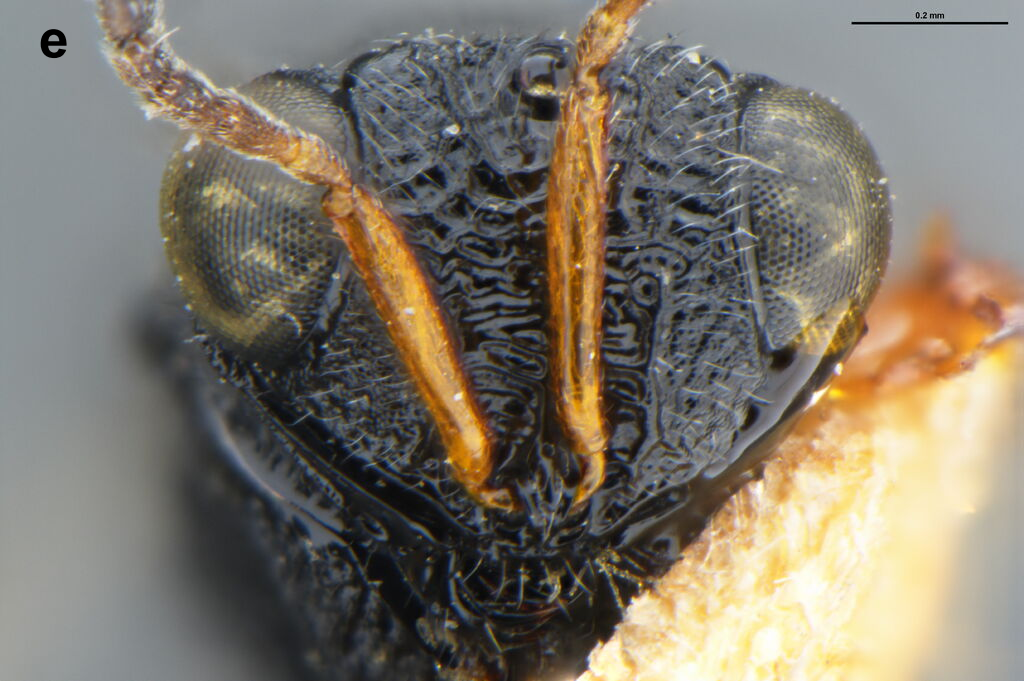
head, anterior view

**Figure 4f.**
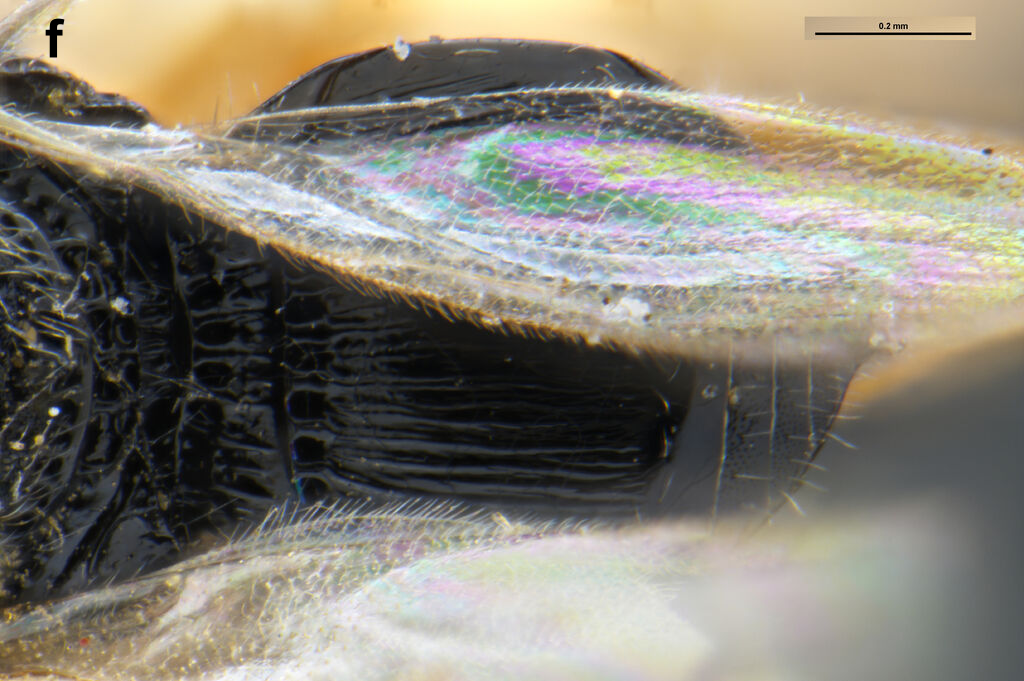
metasoma, dorsal view

**Figure 5a.**
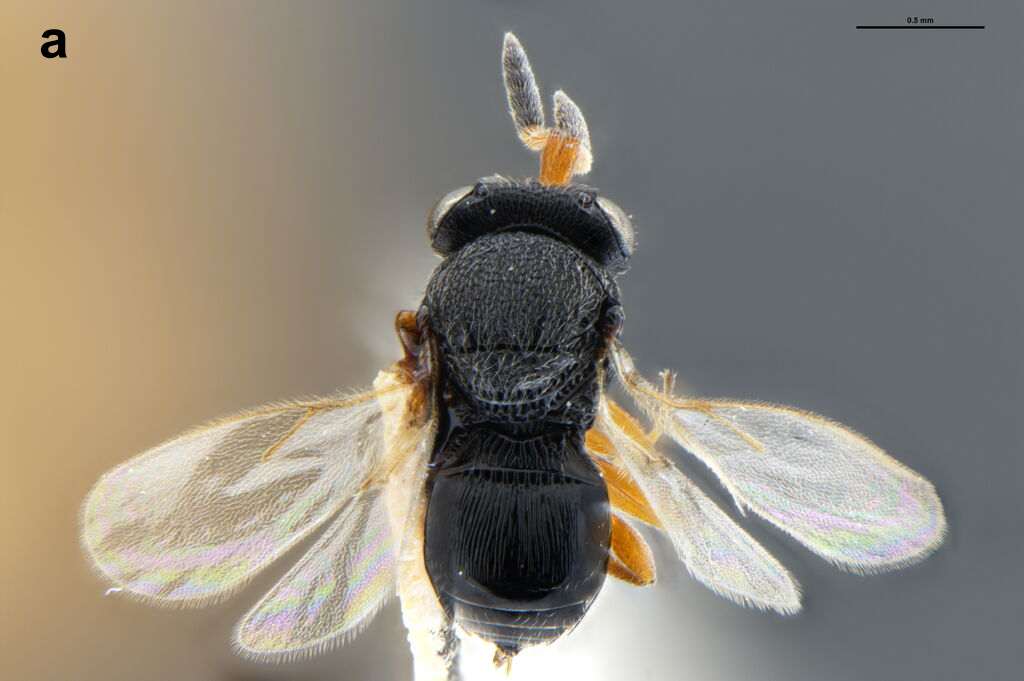
dorsal habitus

**Figure 5b.**
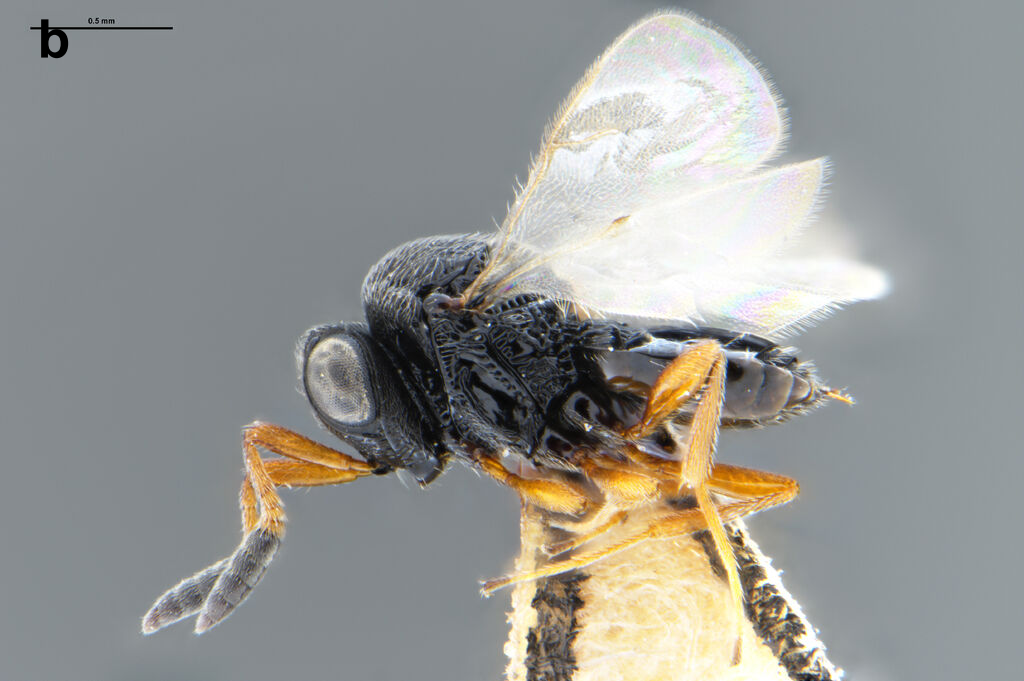
lateral habitus

**Figure 5c.**
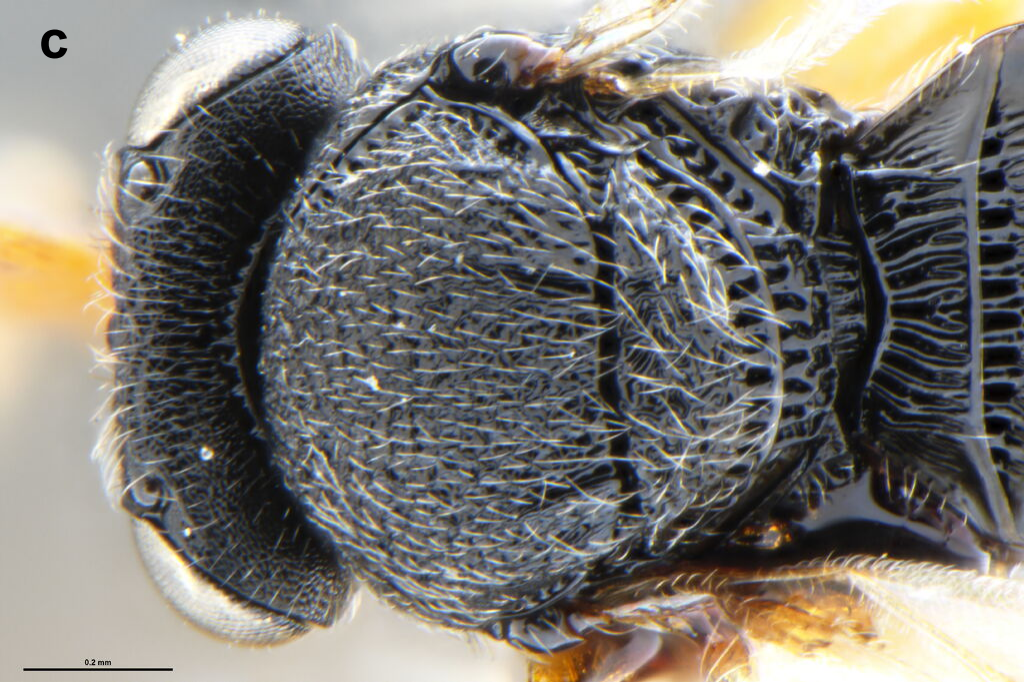
head and mesosoma, dorsal view

**Figure 5d.**
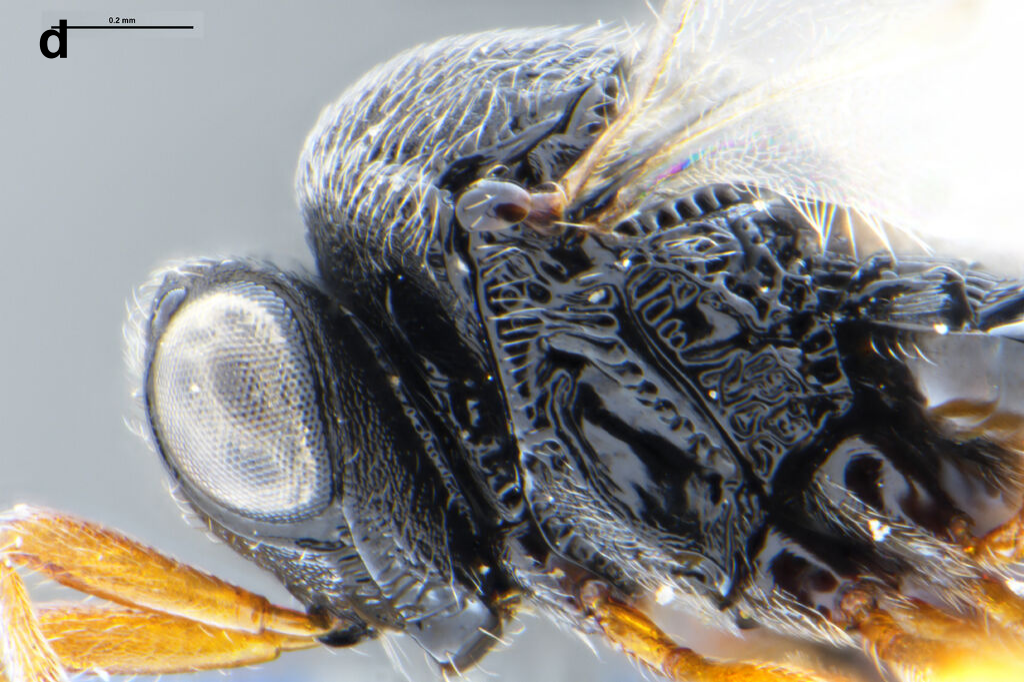
head and mesosoma, lateral view

**Figure 5e.**
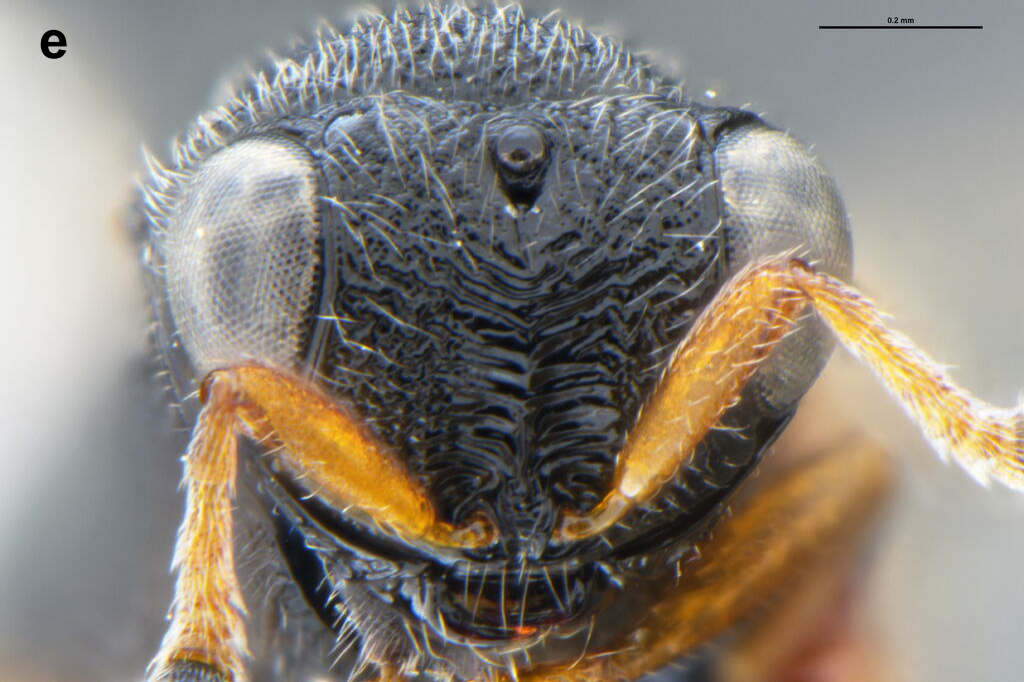
head, anterior view

**Figure 5f.**
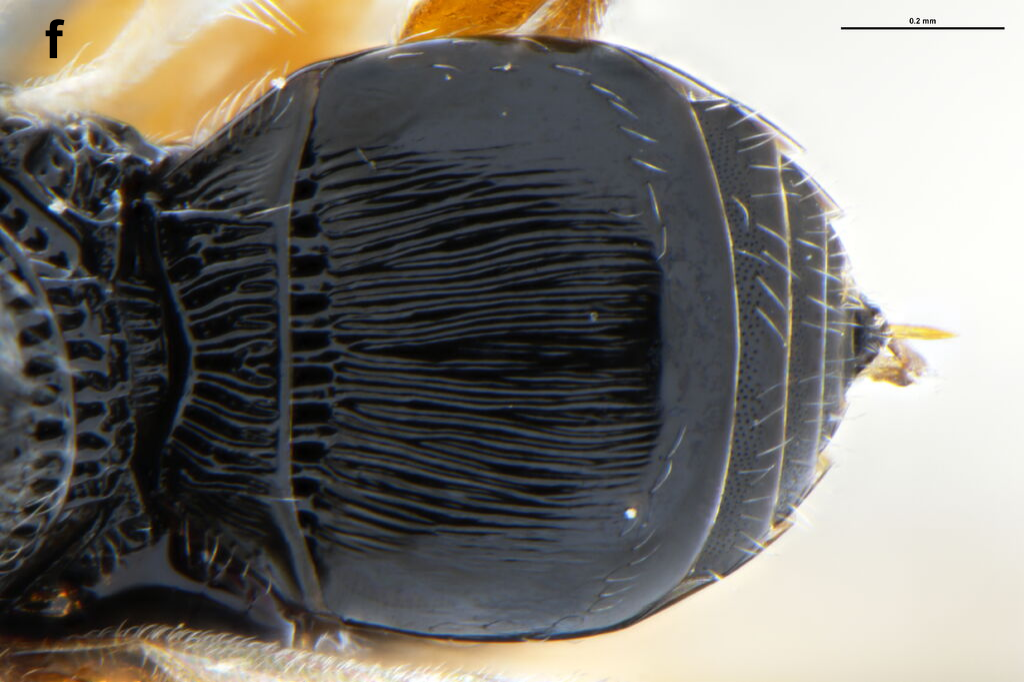
metasoma, dorsal view

**Figure 7a.**
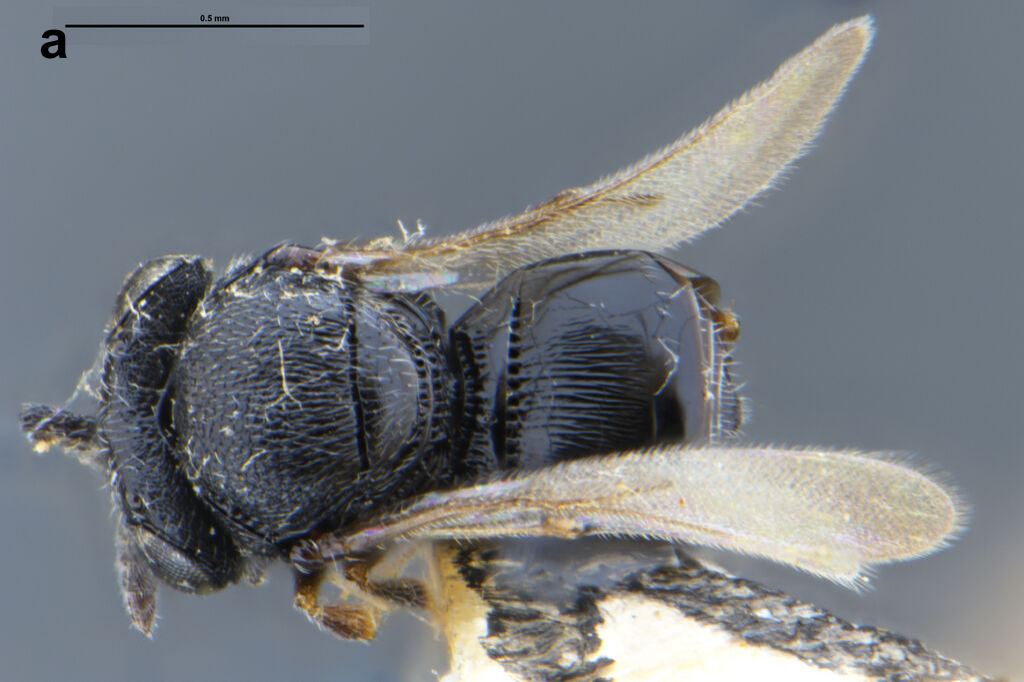
dorsal habitus

**Figure 7b.**
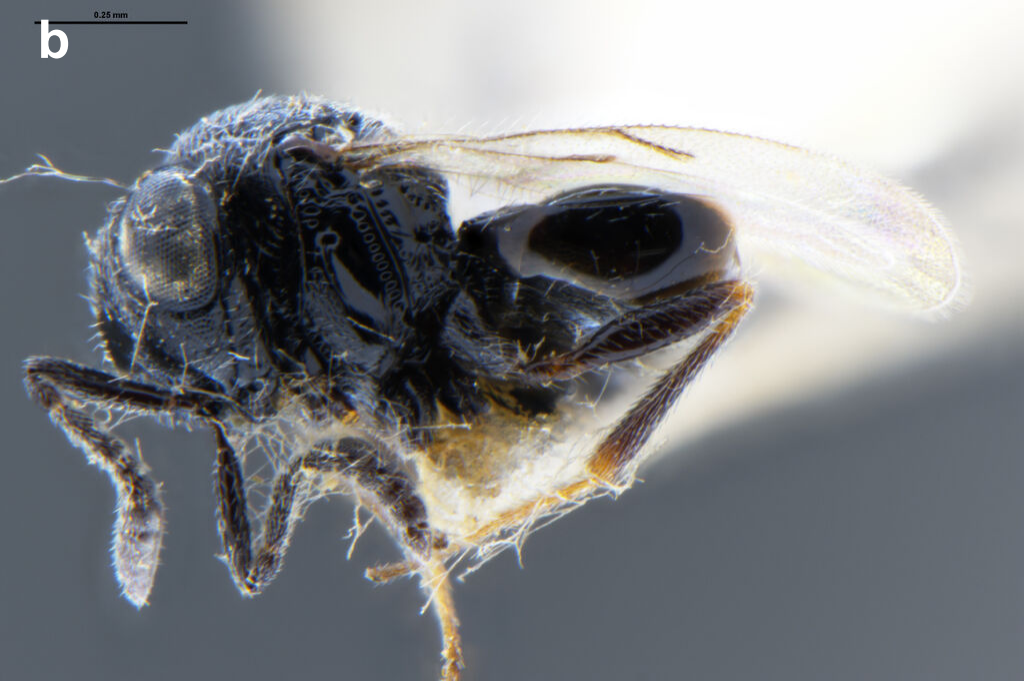
lateral habitus

**Figure 7c.**
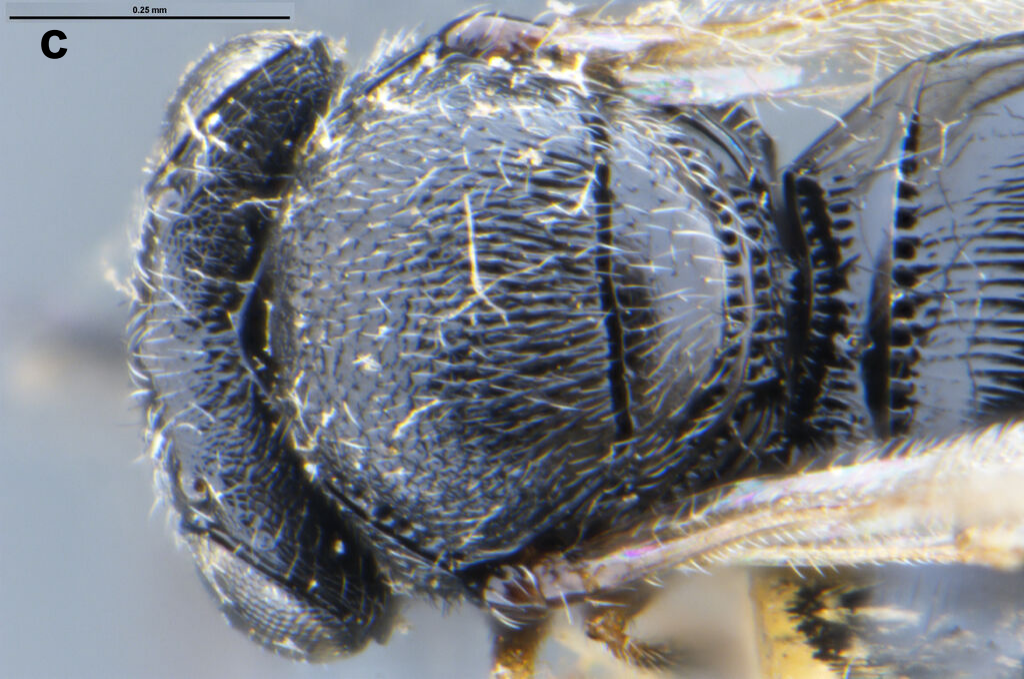
head and mesosoma, dorsal view

**Figure 7d.**
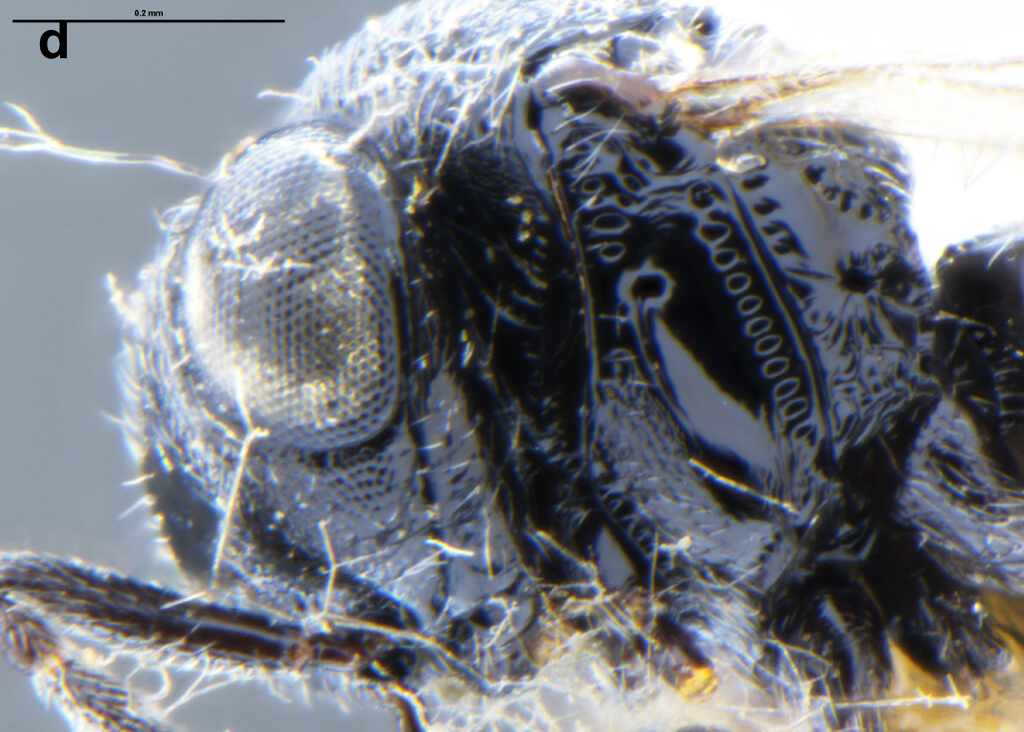
head and mesosoma, lateral view

**Figure 7e.**
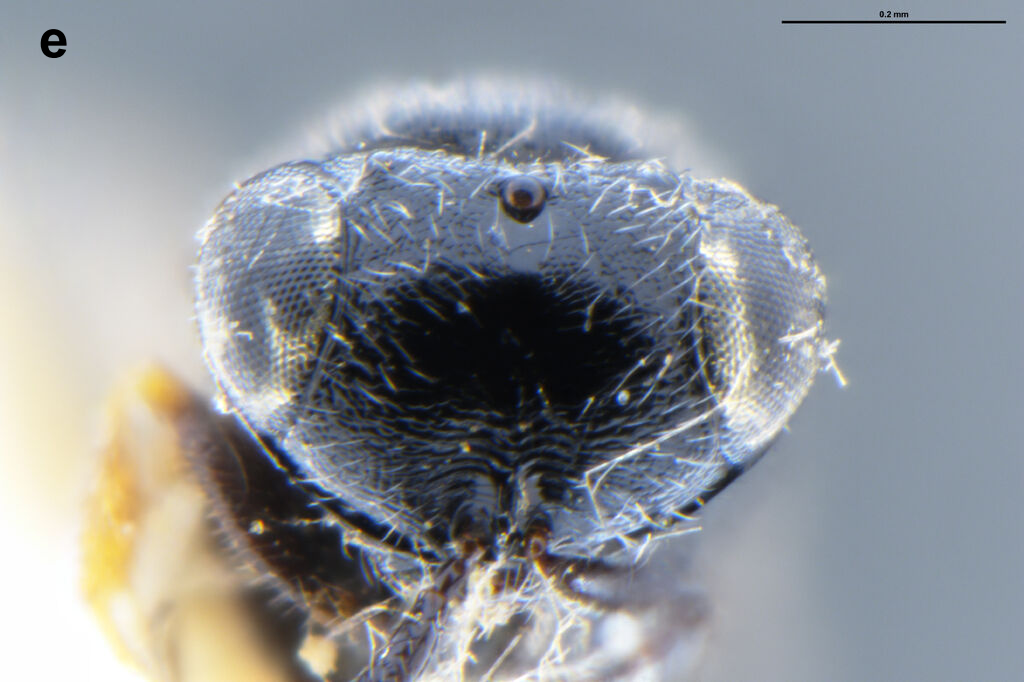
head, anterior view

**Figure 7f.**
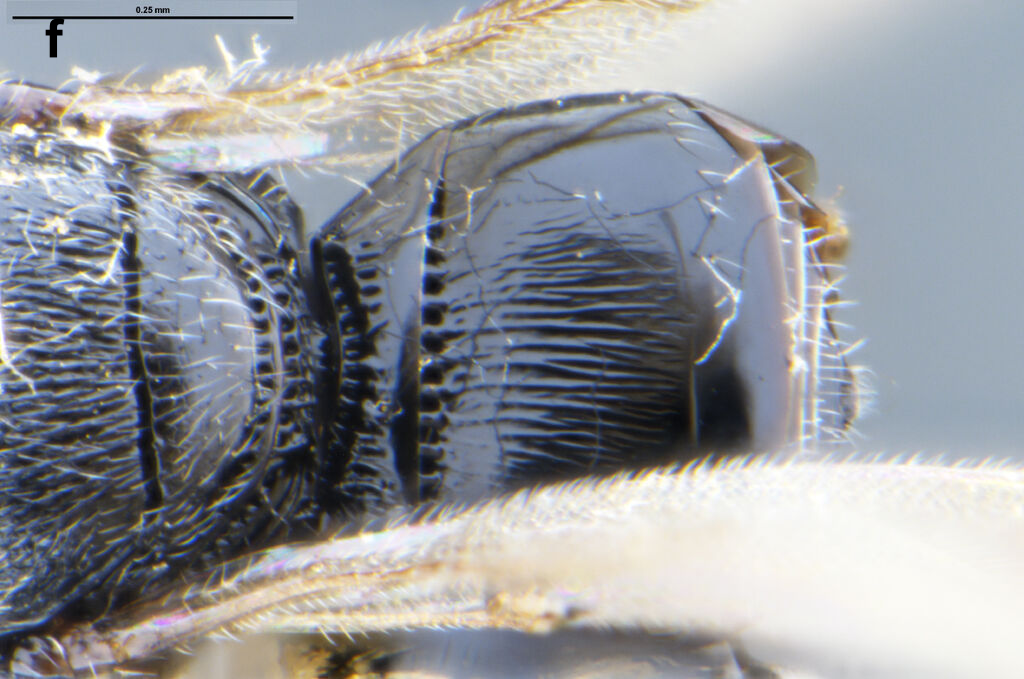
metasoma, dorsal view

**Figure 8a.**
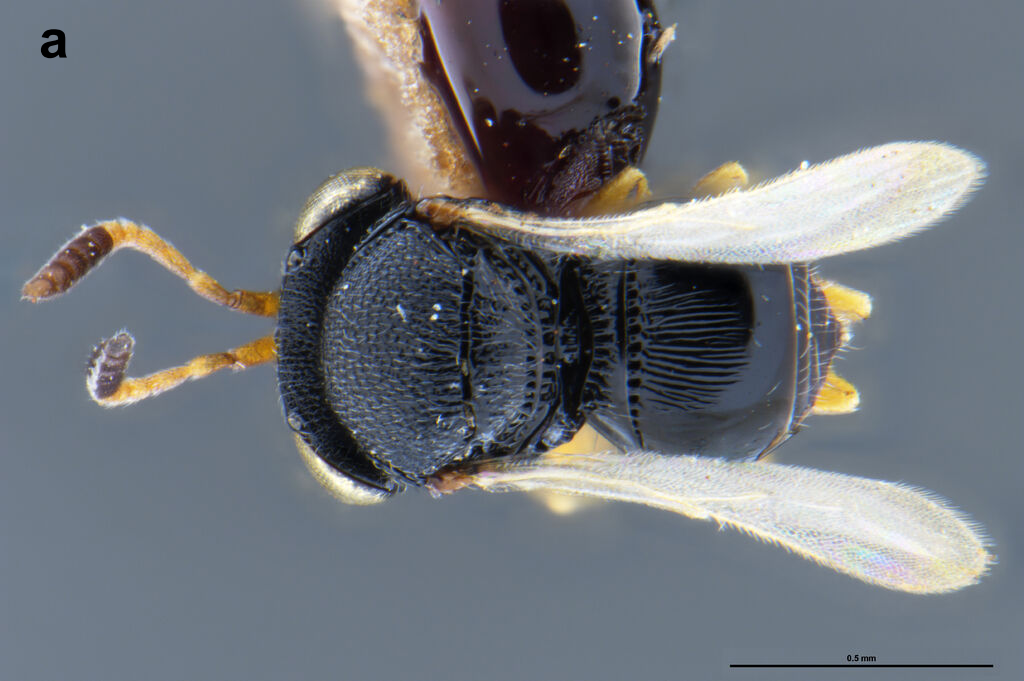
dorsal habitus

**Figure 8b.**
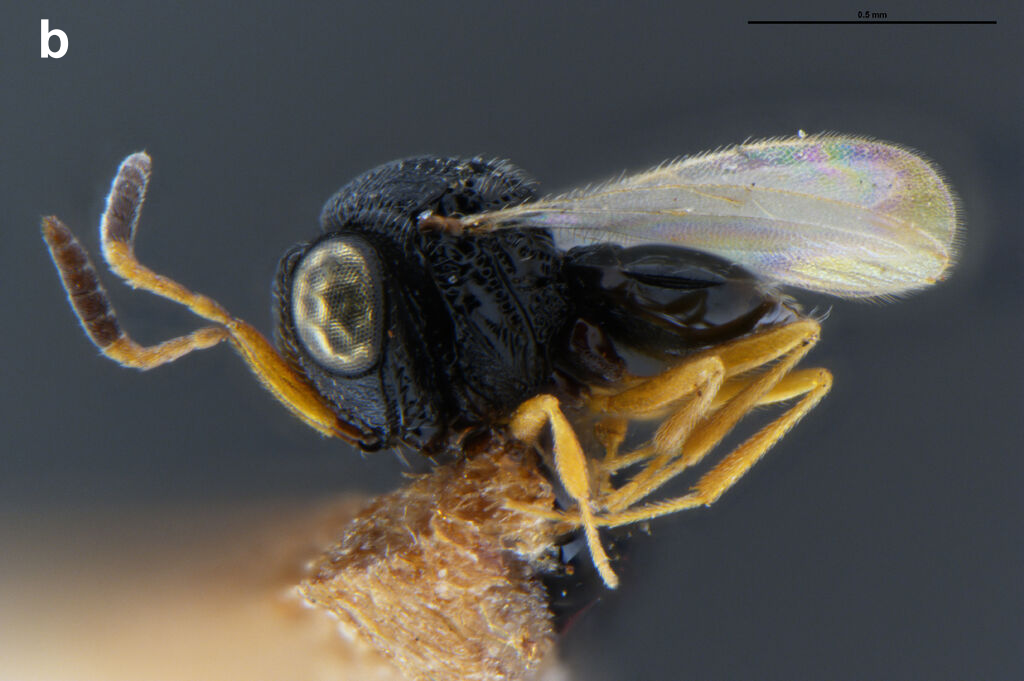
lateral habitus

**Figure 8c.**
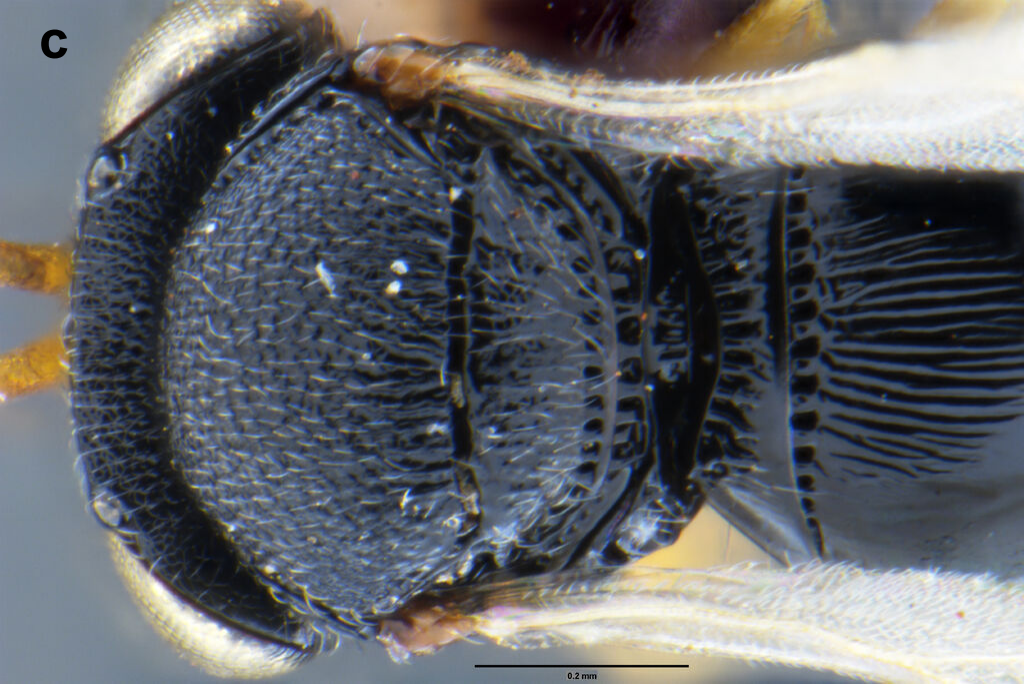
head and mesosoma, dorsal view

**Figure 8d.**
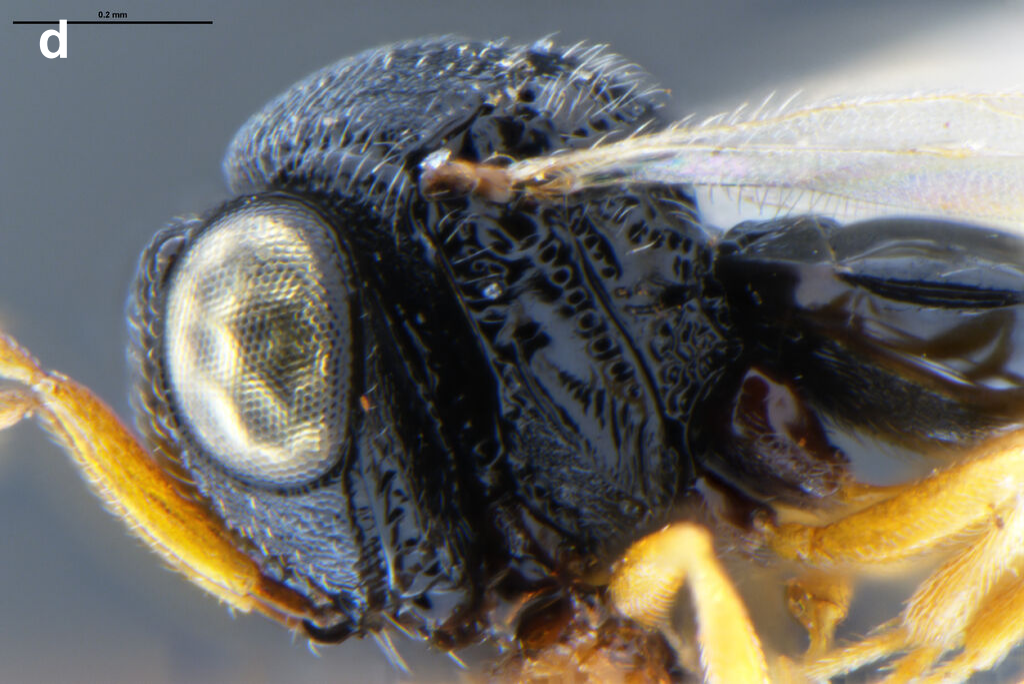
head and mesosoma, lateral view

**Figure 8e.**
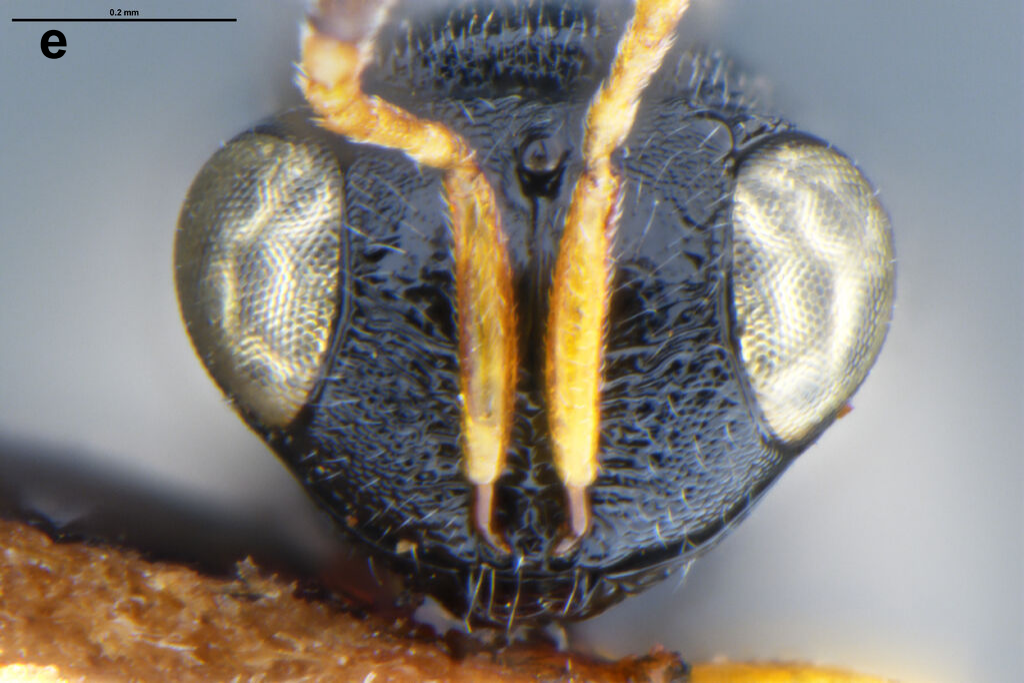
head, anterior view

**Figure 8f.**
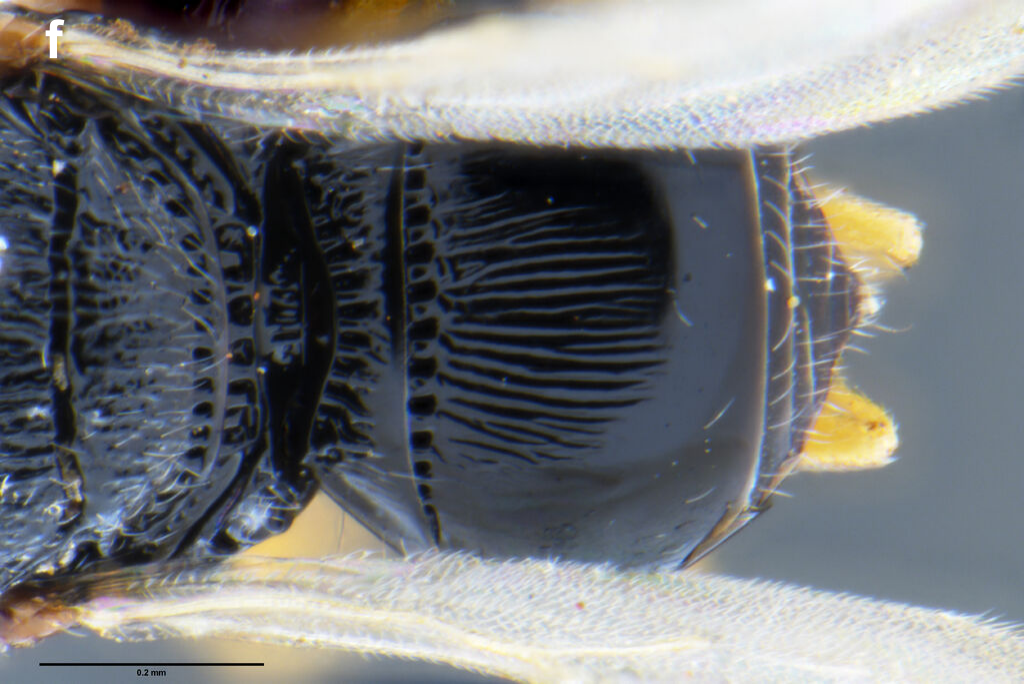
metasoma, dorsal view
